# The complete genome sequence of *Xanthomonas albilineans *provides new insights into the reductive genome evolution of the xylem-limited *Xanthomonadaceae*

**DOI:** 10.1186/1471-2164-10-616

**Published:** 2009-12-17

**Authors:** Isabelle Pieretti, Monique Royer, Valérie Barbe, Sébastien Carrere, Ralf Koebnik, Stéphane Cociancich, Arnaud Couloux, Armelle Darrasse, Jérôme Gouzy, Marie-Agnès Jacques, Emmanuelle Lauber, Charles Manceau, Sophie Mangenot, Stéphane Poussier, Béatrice Segurens, Boris Szurek, Valérie Verdier, Matthieu Arlat, Philippe Rott

**Affiliations:** 1 CIRAD, UMR 385 BGPI, Campus international de Baillarguet, TA A-54K, F-34398 Montpellier Cedex 5, France; 2 Génoscope, Centre national de séquençage, CEA/DSV/IG/Genoscope, 2 rue Gaston Cremieux, F-91057 Evry Cedex, France; 3 Laboratoire des Interactions Plantes Micro-organismes (LIPM), UMR CNRS-INRA 2594/441, F-31320 Castanet-Tolosan, France; 4 Laboratoire Génome et Développement des Plantes, Université de Perpignan via Domitia - CNRS - IRD, UMR 5096, 911 Avenue Agropolis, BP 64501, F-34394 Montpellier cedex 5, France; 5 INRA, UMR 077 PaVé, F-49071 Beaucouzé, France; 6 Agrocampus Ouest centre d'Angers, UMR 077 PaVé, F-49071 Beaucouzé, France; 7 Université de Toulouse, UPS, 118 Route de Narbonne, F-31062 Toulouse, France

## Abstract

**Background:**

The *Xanthomonadaceae *family contains two xylem-limited plant pathogenic bacterial species, *Xanthomonas albilineans *and *Xylella fastidiosa*. *X. fastidiosa *was the first completely sequenced plant pathogen. It is insect-vectored, has a reduced genome and does not possess *hrp *genes which encode a Type III secretion system found in most plant pathogenic bacteria. *X. fastidiosa *was excluded from the *Xanthomonas *group based on phylogenetic analyses with rRNA sequences.

**Results:**

The complete genome of *X. albilineans *was sequenced and annotated. *X. albilineans*, which is not known to be insect-vectored, also has a reduced genome and does not possess *hrp *genes. Phylogenetic analysis using *X. albilineans *genomic sequences showed that *X. fastidiosa *belongs to the *Xanthomonas *group. Order of divergence of the *Xanthomonadaceae *revealed that *X. albilineans *and *X. fastidiosa *experienced a convergent reductive genome evolution during their descent from the progenitor of the *Xanthomonas *genus. Reductive genome evolutions of the two xylem-limited *Xanthomonadaceae *were compared in light of their genome characteristics and those of obligate animal symbionts and pathogens.

**Conclusion:**

The two xylem-limited *Xanthomonadaceae*, during their descent from a common ancestral parent, experienced a convergent reductive genome evolution. Adaptation to the nutrient-poor xylem elements and to the cloistered environmental niche of xylem vessels probably favoured this convergent evolution. However, genome characteristics of *X. albilineans *differ from those of *X. fastidiosa *and obligate animal symbionts and pathogens, indicating that a distinctive process was responsible for the reductive genome evolution in this pathogen. The possible role in genome reduction of the unique toxin albicidin, produced by *X. albilineans*, is discussed.

## Background

The *Xanthomonadaceae *are a family of Gram negative bacteria belonging to the order *Xanthomonadales *in the gamma subdivision of the *Proteobacteria *[1]. Members of this family are typically characterized as environmental organisms and occupy diverse ecological niches, such as soil and water, as well as plant tissues. Many *Xanthomonadaceae*, especially species from the genera *Xanthomonas *and *Xylella*, cause plant diseases and only one, *Stenotrophomonas maltophilia*, is known to be an opportunistic human pathogen.

Complete genome sequences of several *Xanthomonas *species and *Xylella fastidiosa *strains have been determined, making those bacteria attractive models for study of plant-pathogen interactions [2]. *X. fastidiosa *was the first completely sequenced plant pathogen. Sequence analysis showed that this xylem-limited bacterium, which is insect-vectored to a variety of diverse hosts, had a reduced genome and did not possess *hrp *genes, which encode a Type III secretion system (T3SS) found in most Gram negative plant pathogenic bacteria [3]. Phylogenetic analysis with rRNA sequences showed that the two major genera of *Xanthomonadaceae*, *Xanthomonas *and *Stenotrophomonas*, form a coherent group excluding *X. fastidiosa *[4-6]. These characteristics suggested the hypothesis that this species evolved from an ancestor shared with *Xanthomonas *and *Stenotrophomonas *by genome reduction during adaptation to life within its hosts [2].

*Xanthomonas albilineans *is a systemic, xylem-limited pathogen that causes leaf scald, one of the major diseases of sugarcane (interspecific hybrids of *Saccharum *spp.) [7]. Leaf scald symptoms vary from a single, white, narrow, sharply defined stripe to complete wilting and necrosis of infected leaves, leading to plant death. *X. albilineans *produces the toxin albicidin that has phytotoxic and antibiotic properties [8]. Albicidin is a potent DNA gyrase inhibitor that targets the chloroplastic DNA gyrase A, inhibits chloroplast DNA replication and blocks chloroplast differentiation, resulting in the white foliar stripe symptoms [8,9]. All attempts to identify *hrp *genes in *X. albilineans *failed so far [10,11]. A phylogenetic study with the housekeeping genes *ihfA *and *efp*, which did not include *S. maltophilia *sequences, suggested that *X. albilineans *was an evolutionary intermediate between several *Xanthomonas *species and *X. fastidiosa *[11].

Unlike other xylem-invading xanthomonads that interact with living plant tissues, such as *X. campestris *pv. *campestris *or *X. oryzae *pv. *oryzae*, *X. fastidiosa *and *X. albilineans *appear to be strictly xylem-limited, living only in dead xylem cells or tracheary elements. In order to better understand the evolution of these two xylem-limited *Xanthomonadaceae*, we sequenced the genome of *X. albilineans *strain GPE PC73 from Guadeloupe [11]. This sequence was compared to complete genome sequences of other closely related members of the *Xanthomonadaceae*. This comparative analysis revealed that *X. albilineans *and *X. fastidiosa *experienced a convergent reductive genome evolution from a common ancestral parent of the *Xanthomonas *genus.

## Results

### General genomic features of *X. albilineans*

**Figure 1:**
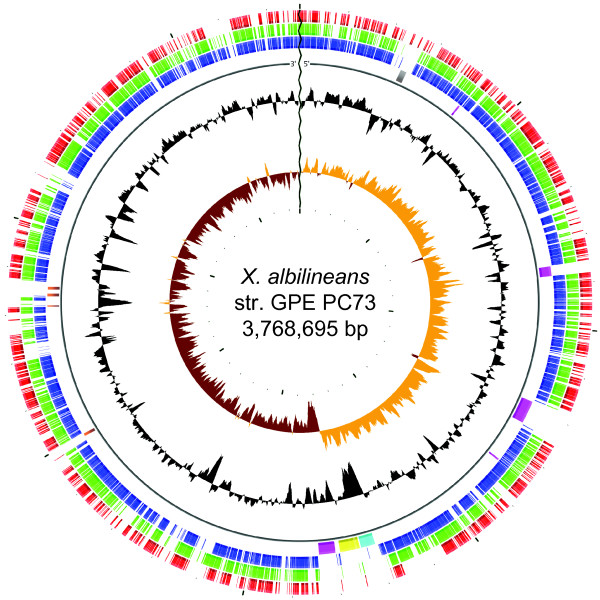
**Circular representation of the *X. albilineans *chromosome (strain GPE PC73)**. The scale is shown in megabases around the periphery. Moving inward, the first three circles show CDSs conserved in *X. fastidiosa *strain 9a5c (in red), *S. maltophilia *strain R551-3 (in green) and *X. axonopodis *pv. *vesicatoria *strain 85-10 (in blue), respectively (forward and reverse-strand conserved CDSs are shown in the same circle). In the next circle, the NRPS gene clusters (except the albicidin biosynthesis gene cluster) are shown in pink, the albicidin biosynthesis gene cluster is shown in yellow, the T3SS SPI-1 gene cluster is shown in blue, genes encoding proteins that contain repeated Rhs elements are shown in brown, and a large phage-related sequence is shown in grey. The black circle shows the G+C content using a 100 base window. The brown and orange circle shows the GC skew (G-C)/(G+C) using a 100 base window.

**Figure 2:**
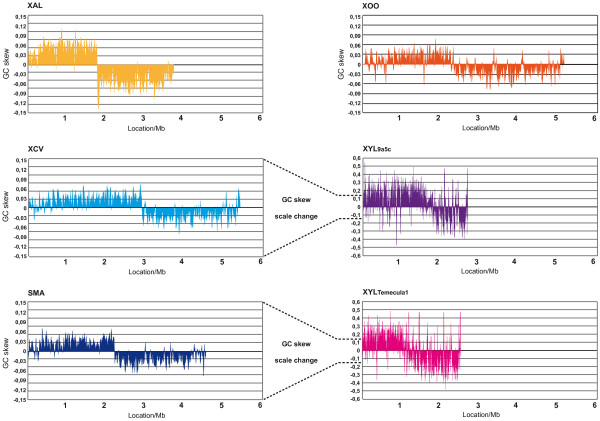
**Linear representation of the GC skew (G-C)/(G+C) of six strains of *Xanthomonadaceae *using a 1000 base window**. XCV = *X. axonopodis *pv.* vesicatoria *strain 85-10; XAL = *X. albilineans *strain GPE PC73; XOO = *X. oryzae *pv. *oryzae *strain MAFF 311018; SMA = *S. maltophilia *strain R551-3; XYL9a5c = *X. fastidiosa *strain 9a5c; XYLTemecula1 = *X. fastidiosa *strain Temecula1. A GC skew pattern very similar to that of *X. axonopodis *pv.* vesicatoria *strain 85-10 was observed for *X. campestris *pv. *campestris *strain ATCC 33913 and *X. axonopodis *pv. *citri *strain 306 (data not shown). A GC skew pattern very similar to that of *S. maltophilia *strain R551-3 was observed for *S. maltophilia *strain K279a (data not shown).

The genome of *X. albilineans *strain GPE PC73 consists of one circular chromosome of 3,768,695 bp and three extrachromosomal plasmids of 32, 27 and 25 Kbp, respectively. The chromosome exhibits a GC skew pattern typical of prokaryotic genomes that have two major shifts, one near the origin and one near the terminus of replication, with *dnaA *assigned as base pair 1 of the chromosome (Figure 1). The GC skew pattern of *X. albilineans *contains a lower number of diagram distortions and has much lower amplitude than the GC skew pattern of *X. fastidiosa *(Figure 2). However, the amplitude of the GC skew pattern of *X. albilineans *is significantly higher than the one of other xanthomonads and *S. maltophilia *(Figure 2). Of the 3115 putative protein-coding sequences (CDSs) manually annotated on the chromosome of *X. albilineans *strain GPE PC73, 2014 (64%) were assigned putative functions based on homology to other known proteins and domain analyses.

The general features of the *X. albilineans *chromosome were compared to those of the chromosomes of the following eight *Xanthomonadaceae *strains: *X. oryzae *pv. *oryzae *strain MAFF 311018 (isolated from rice; [12]), *X. campestris *pv. *campestris *strain ATCC 33913 (isolated from cabbage; [13]), *X. axonopodis *pv. *vesicatoria *strain 85-10 (isolated from pepper; [14]), *X. axonopodis *pv. *citri *strain 306 (isolated from citrus; [13]), *S. maltophilia *strain K279a (isolated from the blood of an infected patient; [15]), *S. maltophilia *strain R551-3 (isolated from poplar; [16]), *X. fastidiosa *strain 9a5c (isolated from citrus; [3]) and *X. fastidiosa *strain Temecula1 (isolated from grapevine; [17]). The chromosome of *X. albilineans *is 1.4 Mb smaller than the chromosomes of *X. axonopodis *pv. *vesicatoria *strain 85-10 and *X. axonopodis *pv. *citri *strain 306, but it is 1.2 Mb bigger than the chromosome of *X. fastidiosa *strain Temecula1 (Table 1). The G+C content of the chromosome of *X. albilineans *averages 63%. This value is similar to those of other *Xanthomonas *strains, but it is 12% higher than the G+C content of the chromosome of *X. fastidiosa *strain Temecula1. The chromosome of *X. albilineans *shows an average coding density of 84%, which is also similar to other xanthomonads.

**Table 1:** General features of nine *Xanthomonadaceae *chromosomes

Features	*X. oryzae *pv. *oryzae *strain MAFF 311018	*X. campestris *pv. *campestris *strain ATCC 33913	*X. axonopodis *pv. *vesicatoria *strain 85-10	*X. axonopodis *pv. *citri *strain 306	*S. maltophilia *strain K279a	*S. maltophilia *strain R551-3	*X. fastidiosa *strain 9a5c	*X. fastidiosa *strain Temecula1	*X. albilineans *strain GPE PC73
Size (bp)	4,940,217	5,076,187	5,178,466	5,175,554	4,851,126	4,573,969	2,679,306	2,519,802	3,768,695
G+C content (%)	63	65	65	64	66	66	52	51	63
Coding density (%)	83	84	87	84	88	89	83	79	84
Protein-coding sequences (CDS)	4372	4181	4487	4312	4386	4039	2766	2123	3115
Average length of all CDS (bp)	948	1027	1005	1032	980	1010	805	964	1059
Average length of the^a ^core genome CDS (bp)	1055	1058	1060	1056	1051	1048	1048	1044	1050
CDS < 300 bp	346	318	428	299	294	261	738	194	283
rRNA operons	2	2	2	2	4	4	2	2	2
tRNA	53	54	54	54	74	71	49	49	51

^a^core genome CDS correspond to the 1256 orthologs shared by the nine *Xanthomonadaceae*

### Comparative genomic analyses

**Figure 3:**
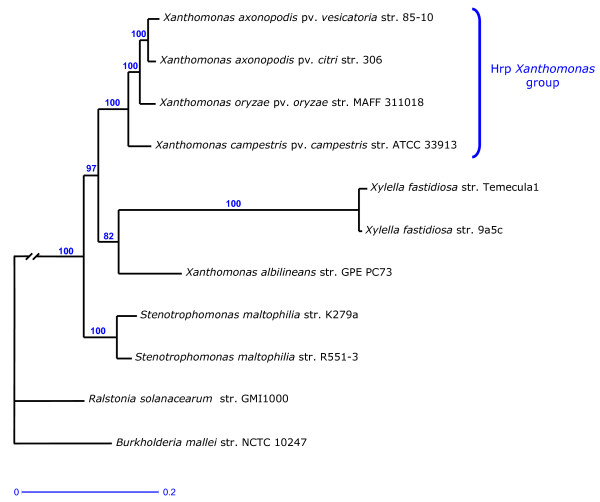
**Tree of the concatenated nucleotide sequences of seven housekeeping genes (*gyrB*, *atpD*, *dnaK*, *efp*, *groEL*, *glnA *and *recA*) using the maximum likelihood method and GTR as substitution model**. The tree was constructed with *Burkholderia pseudomallei *strain NCTC 10247 as outgroup. The total length of the concatenated nucleotide sequences was between 10417-10686 bp. Bootstrap percentages retrieved in 500 replications are shown at the nodes. The scale bar (0.2) indicates the number of nucleotide substitutions per site. The long branch separating the *Xanthomonadaceae *from the two other distant taxa (*B. pseudomallei *strain NCTC 10247 and *Ralstonia solanacearum *strain GMI1000) has been shortened.

In order to assess phylogenetic relationships among the nine *Xanthomonadaceae *mentioned above (Table 1), we performed multilocus sequence analysis (MLSA). The phylogenetic tree obtained with the concatenated data set of seven housekeeping genes showed that *X. albilineans *belongs to the same clade as *X. fastidiosa *(Figure 3). The clade containing *X. albilineans *and *X. fastidiosa *is clearly distinct from the clade containing the four xanthomonads of the Hrp *Xanthomonas *group (*X. campestris *pv. *campestris*, *X. axonopodis *pv. *vesicatoria*, *X. axonopodis *pv. *citri *and *X. oryzae *pv. *oryzae*). On the basis of this phylogenetic tree, we conclude that *X. albilineans *and *X. fastidiosa *derived from the progenitor of the *Xanthomonas *genus (Figure 3).

In order to identify orthologs shared by the nine *Xanthomonadaceae *strains (Table 1), we performed OrthoMCL comparative analyses [18]. The *X. albilineans *CDSs shared with *X. fastidiosa *strain 9a5c, *S. maltophilia *strain K279a or *X. axonopodis *pv. *vesicatoria *strain 85-10 are represented on the circular chromosome (Figure 1). Interestingly, the chromosome of *X. albilineans *harbours several large regions that do not contain any genes present in the other eight *Xanthomonadaceae *strains. These regions correspond either to phage related sequences or to large genes encoding proteins that contain repeated *Rhs *elements that are known to be frequently transferred by horizontal genetic transfer [19]. They also contain several gene clusters specific to *X. albilineans*: (i) the albicidin biosynthesis gene cluster XALB1 which was previously sequenced from *X. albilineans *strain Xa23R1 [20] and which contains three nonribosomal peptide synthetases (NRPSs) genes; (ii) three additional NRPS gene clusters to which cannot be ascribed a precise function as they have not been previously identified and no strictly orthologous genes were found in other bacteria; and (iii) a gene cluster encoding a T3SS of the SPI-1 (*Salmonella *Pathogenicity Island -1) family that is mainly found in free-living animal pathogens [21]. The OrthoMCL analyses identified a total of 522 CDSs of the genome of *X. albilineans *strain GPE PC73 that are not shared with any of the other eight *Xanthomonadaceae *complete genome sequences compared in this study. They also identified 1256 CDSs shared by the nine *Xanthomonadaceae*, which represent a *Xanthomonadaceae *core gene set which was likely inherited from a common ancestor.

### Evidence of convergent genome reductive evolution of *X. albilineans *and *X. fastidiosa*

**Figure 4:**
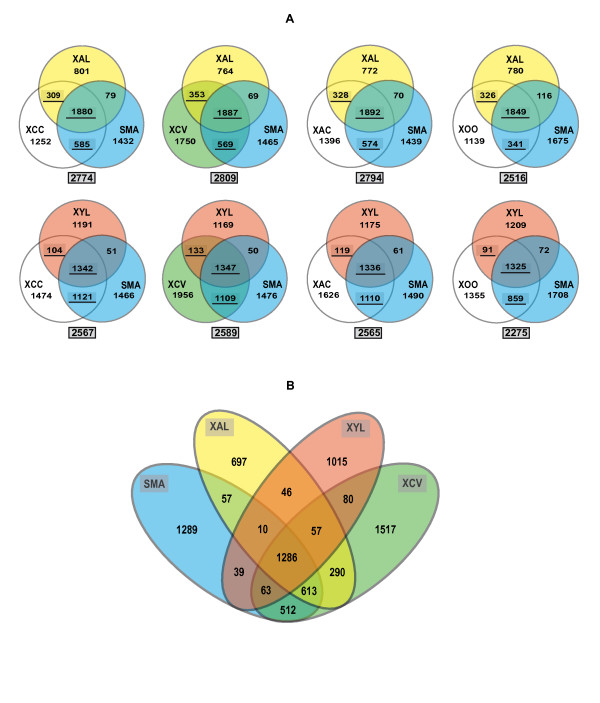
**Venn diagrams showing the number of orthologous CDSs as determined by OrthoMCL analyses among strains of *Xanthomonadaceae***. (A) Venn diagrams showing the number of orthologous CDSs among (i) *X. albilineans *strain GPE PC73 (XAL), *S. maltophilia *strain R551-3 (SMA) and one of the four following Hrp *Xanthomonas *strains: *X. campestris *pv. *campestris *strain ATCC 33913 (XCC), *X. axonopodis *pv. *vesicatoria *strain 85-10 (XCV), *X. axonopodis *pv. *citri *strain 306 (XAC) or *X. oryzae *pv. *oryzae *strain MAFF 311018 (XOO), and (ii) *X. fastidiosa *strain 9a5c (XYL), *S. maltophilia *strain R551-3 (SMA) and one of the four following Hrp *Xanthomonas *strains: *X. campestris *pv. *campestris *strain ATCC 33913 (XCC), *X. axonopodis *pv. *vesicatoria *strain 85-10 (XCV), *X. axonopodis *pv. *citri *strain 306 (XAC) or *X. oryzae *pv. *oryzae *strain MAFF 311018 (XOO). The number of predicted ancestral CDSs of respectively XCC, XCV, XAC and XOO (CDSs conserved in SMA, XAL or XYL) are underlined and the total number of these predicted ancestral CDSs is indicated below each Venn diagram. (B) Venn diagram showing the number of orthologous CDSs among (i) *X. albilineans *strain GPE PC73 (XAL), *S. maltophilia *strain R551-3 (SMA), *X. axonopodis *pv. *vesicatoria *strain 85-10 (XCV) and *X. fastidiosa *strain 9a5c (XYL). Numbers do not include paralogous CDSs.

The phylogenetic tree presented in Figure 3 suggests that *X. albilineans, X. fastidiosa *and the Hrp *Xanthomonas *group derived from a common ancestor shared with *Stenotrophomonas*. The chromosome sizes of *X. albilineans *and *X. fastidiosa *are smaller than those of any other xanthomonad (Table 1), suggesting that both species evolved from the progenitor of xanthomonads by genome reduction. To examine how the chromosomes of *X. albilineans *and *X. fastidiosa *have evolved to result in these different sizes, we determined the order of divergence of *X. albilineans*, *X. fastidiosa*, the four xanthomonads of the Hrp *Xanthomonas *group and *S. maltophilia *(Figure 3). Orthologous genes shared by *S. maltophilia *and a species of the Hrp *Xanthomonas *group, but missing in *X. albilineans *or *X. fastidiosa*, may be assumed to have been lost by *X. albilineans *or *X. fastidiosa*, respectively. Inversely, genes of a species of the Hrp *Xanthomonas *group that are shared with *S. maltophilia*, *X. albilineans *or *X. fastidiosa *may be assumed to have been inherited from the progenitor of the *Xanthomonas *genus. On the basis of OrthoMCL analysis, the numbers of genes lost by *X. albilineans *strain GPE PC73 and *X. fastidiosa *strain 9a5c are at least 585 and 1121, respectively (numbers obtained by comparison with *X. campestris *pv. *campestris*; 585 genes lost by *X. albilineans *= the number of CDSs conserved in both *X. campestris *pv. *campestris *and *S. maltophilia *and missing in *X. albilineans*; 1121 genes lost by *X. fastidiosa *= the number of CDSs conserved in both *X. campestris *pv. *campestris *and *S. maltophilia *and missing in *X. fastidiosa*; Figure 4A). The number of ancestral genes inherited from the progenitor of the *Xanthomonas *genus is higher in *X. axonopodis *pv. *vesicatoria *than in any other species of the Hrp *Xanthomonas *group (2809 ancestral genes present in *X. axonopodis *pv. *vesicatoria *= the number of CDSs of *X. axonopodis *pv. *vesicatoria *conserved in *X. albilineans *or *S. maltophilia*; Figure 4A). Interestingly, *X. oryzae *pv. *oryzae *strain MAFF 311018 contains the smallest number of genes lost by *X. albilineans *or *X. fastidiosa*, indicating that a significant number of genes lost by *X. albilineans *or *X. fastidiosa *were also lost by the xylem-invading pathogen *X. oryzae *pv. *oryzae *(Figure 4A). On the basis of OrthoMCL analysis including both *X. albilineans *strain GPE PC73 and *X. fastidiosa *strain 9a5c, 512 ancestral genes were lost by both *X. albilineans *and *X. fastidiosa *(512 = number of orthologs shared only by *X. axonopodis *pv. *vesicatoria *strain 85-10 and *S. maltophilia *strain R551-3, Figure 4B), 960 ancestral genes were lost only by *X. fastidiosa *(960 = 613 + 290 + 57 = number of CDSs of *X. albilineans *strain GPE PC73 conserved in *X. axonopodis *pv. *vesicatoria *strain 85-10 or *S. maltophilia *strain R551-3 and missing in *X. fastidiosa *strain 9a5c, Figure 4B), and 182 ancestral genes were lost only by *X. albilineans *(182 = 63 + 39 + 80 = number of CDSs of *X. fastidiosa *strain 9a5c conserved in *X. axonopodis *pv. *vesicatoria *strain 85-10 or *S. maltophilia *strain R551-3 and missing in *X. albilineans *strain GPE PC73, Figure 4B).

### Comparison of the reductive evolutions of *X. albilineans *and *X. fastidiosa*

**Figure 5:**
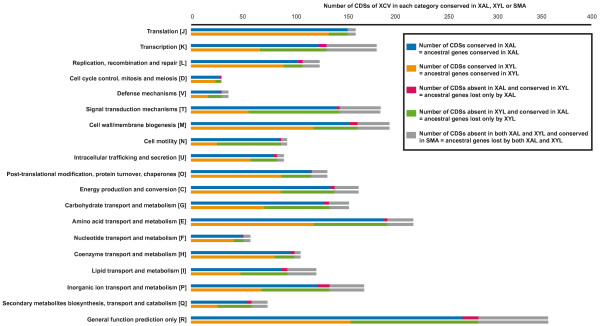
**Distribution in each functional COG category of the putative ancestral genes of *X. axonopodis *pv.*vesicatoria *that were conserved or lost by *X. albilineans *or *X. fastidiosa***. These putative ancestral genes correspond to the 2816 CDSs of the *X. axonopodis *pv.*vesicatoria *strain 85-10 chromosome shared with *S. maltophilia *strain R551-3, *S. maltophilia *strain K279a, *X. albilineans *strain GPE PC73 or *X. fastidiosa *strain 9a5c. They are listed and individually analysed in Additional file 1. XCV = *X. axonopodis *pv.*vesicatoria *strain 85-10; XAL = *X. albilineans *strain GPE PC73; SMA = *S. maltophilia *strain R551-3 or *S. maltophilia *strain K279a; XYL = *X. fastidiosa *strain 9a5c.

Further comparative analyses were performed to compare genome erosion in *X. albilineans *and *X. fastidiosa*. For these analyses, we selected the genome of *X. axonopodis *pv. *vesicatoria*, which contains the highest number of genes inherited from the progenitor of the *Xanthomonas *genus (Figure 4A). OrthoMCL analysis identified 3004 CDSs of *X. axonopodis *pv. *vesicatoria *that do not include any transposase genes and that are shared either by *X. albilineans *strain GPE PC73, *X. fastidiosa *strain 9a5c or one of the two *S. maltophilia *strains K279a and R551-3. For each of the 3004 CDSs, we looked for the best BLAST hit within a database that included: (i) all annotated CDSs from the genome sequence of *X. albilineans *strain GPE PC73 and (ii) all available sequences from public databases excluding all sequences from the xanthomonads. On the basis of these analyses and among the 3004 CDSs, we selected the genes for which the best BLAST hit belonged to *X. albilineans*, *X. fastidiosa *or *S. maltophilia*. We made the hypothesis that these genes were inherited by *X. axonopodis *pv. *vesicatoria *from the ancestor of the xanthomonads. These best BLAST hit analyses confirmed that 2864 of these 3004 CDSs have the same ancestor as genes present either in *X. albilineans*, *X. fastidiosa *or *S. maltophilia *and were therefore inherited by *X. axonopodis *pv.* vesicatoria *from the progenitor of the *Xanthomonas *genus. The elimination of paralogs present in at least two copies in *X. axonopodis *pv.* vesicatoria *generated a list of 2816 CDSs representing one copy of each gene inherited by *X. axonopodis *pv. *vesicatoria *from the progenitor of the *Xanthomonas *genus. These 2816 CDSs are listed and individually analysed in additional file 1. Among them, 1334 CDSs are shared by both *X. fastidiosa *and *X. albilineans *(these represent the ancestral genes conserved by *X. fastidiosa *and *X. albilineans*), 480 CDSs are shared only with one of the two *S. maltophilia *strains (they represent the ancestral genes lost by both *X. fastidiosa *and *X. albilineans*), 112 CDSs are shared with *X. fastidiosa *but not with *X. albilineans *(they represent the genes lost only by *X. albilineans*) and 890 CDSs are shared with *X. albilineans *but not with *X. fastidiosa *(they represent the genes lost only by *X. fastidiosa*). The distribution in functional COG categories of these 2816 CDSs is illustrated in Figure 5.

Analyses of the arrangement of these 2816 ancestral genes on the chromosome of *X. axonopodis *pv. *vesicatoria *strain 85-10 led to the identification of DNA regions constituting contiguous ancestral genes that are missing in *X. albilineans *or *X. fastidiosa *(Additional file 1). During the speciation of *X. fastidiosa *or *X. albilineans*, the loss of these DNA regions was due either to a single event of deletion or to the cumulative effect of multiple events (pseudogenization and short deletions). For example, the loss in *X. fastidiosa *of the large DNA region constituted by ancestral genes from XCV1928 to XCV2044 seems to be due to a single event of deletion since all these ancestral genes are missing in *X. fastidiosa *(Additional file 1). This large DNA region lost by *X. fastidiosa *encodes all flagellar proteins and several chemotaxis proteins. The sum of the length of the ancestral genes present in this DNA region is 106,626 bp, strongly suggesting that *X. fastidiosa *experienced a single deletion of a DNA fragment of a larger size. The other DNA regions constituting contiguous ancestral genes that are missing in *X. albilineans *or *X. fastidiosa *are shorter and, for this reason, their loss may result either from a single event of deletion or from multiple mutational events. Analysis of the arrangement on the chromosome of *X. axonopodis *pv. *vesicatoria *strain 85-10 of ancestral genes absent in *X. albilineans *or *X. fastidiosa *did not allow us to determine if the genes absent in both xylem-limited *Xanthomonadaceae *were lost by their common ancestor or were lost independently after their divergence.

The loss of genes by pseudogenization and short deletions should not affect the position on the chromosome of the genes that precede or follow the lost genes. In order to identify ancestral genes putatively lost by pseudogenization and short deletions, we looked for ancestral lost genes that are present on the chromosome of *X. axonopodis *pv. *vesicatoria *between the orthologs of two ancestral genes that are contiguous and conserved in *X. fastidiosa *or *X. albilineans*. For example, the *rpf *(for regulation of pathogenicity factors) gene cluster contains in *X. axonopodis *pv. *vesicatoria *two ancestral genes (XCV1913 and XCV1914 which are conserved in *S. maltophilia*) that are missing in both *X. albilineans *and *X. fastidiosa*. The ancestral genes XCV1912 and XCV1915 that precede and follow respectively these two lost genes are orthologs of either Xalc_1342 and Xalc_1343 or XF1110 and XF1111 that are contiguous in *X. albilineans *and *X. fastidiosa*, respectively. Using the same strategy we identified 147 and 131 ancestral genes potentially lost by pseudogenization by *X. fastidiosa *and *X. albilineans*, respectively (Additional files 2 and 3).

### Common genomic features of *X. fastidiosa *and *X. albilineans*

The close relationship between *X. albilineans *and *X. fastidiosa *is illustrated by the common unique characteristics of their enzymes involved in cellulose degradation. In these two xylem-limited *Xanthomonadaceae*, endoglucanase EngXCA and 1,4-beta cellobiosidase CbhA possess a cellulose binding domain (CBD) and a long polyserine linker (PSL) at their C termini (Table 2). The endoglucanase EngXCA is conserved in all other *Xanthomonas *species and also has a CBD, but the linker is much shorter and its serine content is much lower (Table 2). The 1,4-beta cellobiosidase CbhA is conserved in the xylem-invading xanthomonads *X. campestris *pv. *campestris *and *X. oryzae *pv. *oryzae *but, in these two species, CbhA does not possess any linker nor any CBD (Table 2). The presence of a CBD is known to increase catalytic activity by reducing the "substrate accessibility problem" [22]. The long flexible PSL was proposed to enhance substrate accessibility [23]. The presence of genes encoding enzymes harbouring a PSL and a CBD provides evidence that both *X. fastidiosa *and *X. albilineans *are adapted to use plant cell breakdown products as carbon sources.

**Table 2:** Comparative analysis of endoglucanase EngXCA and cellobiosidase CbhA encoded by *Xanthomonadaceae *species

Enzyme	*Xanthomonadaceae *species	Accessions	^a^**PSL size**	^a^**PSL composition**	^b^**CBD**
Endoglucanase EngXCA	*X. campestris *pv. *campestris *strain ATCC 33913	XCC3521	29 Aa	15T, 1G, 1S and 12P	present
	*X. axonopodis *pv. *vesicatoria *strain 85-10	XCV0670	21 Aa	9P, 9T, 1S, 1A and 1G	present
	*X. axonopodis *pv. *citri *strain 306	XAC0612	19 Aa	8P, 8T, 1S, 1A and 1G	present
	*X. oryzae *pv. *oryzae *strain MAFF 311018	XOO_3789	33 Aa	15P, 15T, 2S and 1A	present
	*X. albilineans *strain GPE PC73	XALc_2969	96 Aa	62G, 18S, 15T, 1P	present
	*X. albilineans *strain GPE PC73	XALc_2967	26 Aa	19G, 2S and 5N	present
	*X. fastidiosa *strain 9a5c	XF0818	132 Aa	93G, 38S and 1T	present
	*X. fastidiosa *strain Temecula1	PD1851	157 Aa	84G, 43S, 3T, 26A and 1R	present

Cellobiosidase CbhA = GuxA	*X. campestris *pv. *campestris *strain ATCC 33913	XCC3534	no linker	/	absent
	*X. campestris *pv. *campestris *strain ATCC 33913	XCC3160	no linker	/	absent
	*X. oryzae *pv. *oryzae *strain MAFF 311018	XOO_3805	no linker	/	absent
	*X. albilineans *strain GPE PC73	XALc_0484	152 Aa	99G, 27S and 26T	present
	*X. fastidiosa *strain 9a5c	XF1267	146 Aa	31G, 99S, 4P, 4A, 4F and 4N	present
	*X. fastidiosa *strain Temecula1	PD0529	106 Aa	15G, 78S, 2P, 5T, 2A, 2F and 2N	present

^a^PSL = Polyserine linker; ^b^CBD = Cellulose binding domain

The OrthoMCL analyses identified 18 orthologs shared only by *X. fastidiosa *strain 9a5c, *X. fastidiosa *strain Temecula1 and *X. albilineans *GPE PC73 (corresponding to the CDSs conserved in these three strains that are missing in all the other six *Xanthomonadaceae *genome sequences analysed herein). BLAST analyses confirmed that 11 of these 18 CDSs are unique to *X. albilineans *and *X. fastidiosa *(Additional file 4). Interestingly, they include the gene *metE *which encodes the 5-methyltetrahydropteroyltriglutamate-homocysteine methyltransferase. This enzyme is absolutely required for the biosynthesis of methionine and is therefore present in all *Xanthomonadaceae*. However, the *metE *gene present in the two xylem-limited *Xanthomonadaceae *is closer to the gene *metE *of *Mesorhizobium *sp. (amino acid identity = 466/764 = 60%) than the *metE *present in the other *Xanthomonas *species and in *S. maltophilia *(amino acid identity 89/346 = 25%). This strongly suggests that the progenitor of the two xylem-limited *Xanthomonadaceae *lost the ancestral *metE *gene (which was conserved in other *Xanthomonas *species and *S. maltophilia*) and acquired another *metE *by horizontal genetic transfer. The 11 genes unique to *X. albilineans *and *X. fastidiosa *also include one cysteine protease gene, one ABC transporter gene, one polysaccharide deacetylase gene, one glycosyl transferase gene, one hydrolase gene, one cell filamentation protein gene and four hypothetical protein genes (Additional file 4).

BLAST analyses confirmed that *X. albilineans*, like *X. fastidiosa*, lacks the Hrp T3SS that is present in other *Xanthomonas *species and does not possess any of the known Hrp type III effectors. The Hrp T3SS, which plays a major role in suppressing host plant defense responses in most other pathogenic *Xanthomonas *strains [24], was therefore probably acquired after the divergence of the Hrp *Xanthomonas *group and xylem-limited *Xanthomonadaceae *lineages. No remains of the Hrp gene cluster were found in the complete genome sequence of *X. albilineans *strain GPE PC73 nor in the available complete genome sequences of *X. fastidiosa*.

## Discussion

In their rather cloistered environmental niche inside xylem vessels, *X. albilineans *and *X. fastidiosa *may have largely avoided surveillance by general and specific plant defense systems. Their lack of a T3SS of the Hrp1 or Hrp2 families may be explained by the fact that *X. albilineans *and *X. fastidiosa *live and multiply essentially in a dead-cell environment. However, like other bacterial vascular pathogens, they may interact with living xylem parenchyma cells through pit membranes [25]. If they do, they do not use a Hrp TTSS but another system that remains to be identified. The adaptation of *X. albilineans *and *X. fastidiosa *to a xylem-limited lifestyle is also illustrated by their enzymes adapted to the use of plant cell breakdown products as carbon sources. The low number of genes unique to both *X. albilineans *and *X. fastidiosa *(11, see Additional file 4) may be explained by a very early divergence of the two xylem-limited *Xanthomonadaceae *lineages, possibly followed by strong selective pressure to adapt to their different biological niches and lifestyles. *X. fastidiosa *is vector-transmitted by various xylem sap-feeding insects and is able to colonize many plant species (citrus, wine grape, coffee, alfalfa, peach, plum, almond, elm, maple, pear, etc) (reviewed in [26]). On the other hand, *X. albilineans *is mainly transmitted by mechanical means and is not known to be insect-transmitted, and is able to colonize only sugarcane and few other monocots in the *Poaceae *family (reviewed in [8]).

The genome of *X. albilineans *encodes a T3SS that displays similarities with the *Burkholderia pseudomallei *bsa T3SS which belongs to the injectisome family SPI-1 (*Salmonella *Pathogenicity Island -1) and which is required for the virulence of this human pathogen. The SPI-1 injectisome family mainly includes T3SSs from human and insect bacterial pathogens or symbionts [21]. Interestingly, the genomes of *Erwinia amylovora *strain Ea273 and *Erwinia tasmaniensis *strain Et1/99 both contain two copies of a SPI-1 T3SS [27,28]. The role of these SPI-1 T3SSs in these plant-invading *Erwinia *spp. remains unknown. *E. amylovora *is insect-disseminated, although the interactions between this pathogen and its insect hosts remain poorly understood. It was suggested that the presence of a SPI-1 T3SS in *Erwinia *spp. indicates a common ancestry and close phylogenetic relationship between *Erwinia *spp. and insect-related enteric bacteria, raising the possibility that an insect host might be serving as a mixing vessel for the exchange of genes between *Erwinia *strains and other enteric bacteria [27]. Similarly, the presence of a SPI-1 T3SS in the genome of *X. albilineans *could indicate an insect-associated life style of this plant pathogen.

The MLSA performed herein resulted in a phylogenetic tree that included *X. fastidiosa *into the *Xanthomonas *group. This phylogenetic tree is in accordance with the presence of the unique *gum *genes in both *X. fastidiosa *and *Xanthomonas *species of the Hrp *Xanthomonas *group. The *gum *genes, which are involved in the biosynthesis of extracellular polysaccharides and the formation of biofilms, play a key role in pathogenicity of these *Xanthomonadaceae*. These genes, which were probably acquired by the progenitor of the *Xanthomonas *genus, were most likely lost by *X. albilineans *and conserved by *X. fastidiosa *during their speciation. Our MLSA phylogenetic tree is also in accordance with i/the presence of 11 unique genes, including *metE*, in *X. albilineans *and *X. fastidiosa*, and ii/the alignment of the 5' end of the 16S RNA of *Xanthomonadaceae *(Additional file 5).

Additionally, based on this MLSA, the same 480 ancestral genes appeared to be lost by both *X. albilineans *and *X. fastidiosa*. Interestingly, 209 of the 480 ancestral genes lost by both *X. albilineans *and *X. fastidiosa *are also absent in *X. oryzae *pv. *oryzae *(a xylem invading pathogen belonging to another phylogenetic clade), indicating that independent but convergent evolution events were involved in genome erosion of *X. oryzae *pv.* oryzae *and the xylem-limited *Xanthomonadaceae*. Some of these genes lost by three xylem-invading pathogens are orthologous of genes with assigned functions and are organized into clusters. The five following ancestral gene clusters were lost by *X. albilineans*, *X. fastidiosa *and *X. oryzae *pv. *oryzae*: i/ the ancestral genes XCV0258 to XCV265 encoding enzymes involved in the glyoxylate cycle; ii/ the ancestral genes XCV0592 to XCV0602 encoding enzymes involved in malonate metabolism; iii/ the ancestral genes XCV1316 to XCV1334 including one TonB-dependant receptor gene, a two component signal transduction system (TCSTS) and chemotaxis genes, iv/ the ancestral genes XCV2187 to XCV2196 including one TCSTS and a type I secretion system and v/ the ancestral genes XCV2796 to XCV2803 encoding enzymes involved in catabolism of polysaccharides (Additional file 1). These examples support the hypothesis of a link between the convergent erosion of three xylem-invading *Xanthomonadaceae *and the adaptation to a same restricted environment (the xylem) in which these lost functions are useless. However, only 38 of the 480 ancestral genes lost by both *X. albilineans *and *X. fastidiosa *are also absent in another xylem invading pathogen, *X. campestris *pv. *campestris*, indicating that adaptation to xylem lifestyle favoured or allowed genome erosion, but did not necessarily induce it. Alternatively, the convergent genome erosion of the two xylem-limited *Xanthomonadaceae *may be linked to similar insect-associated lifestyles that may have favoured genome erosion because most of the genes required for a plant-associated life style are most likely not required for an insect-associated life style.

Similar striking convergence in fundamental genomic features associated with a restricted lifestyle is very well documented for obligate animal symbionts and pathogens, especially for *Buchnera *(reviewed in [29]). In these bacteria, gene losses are non-random but can affect all functional categories. The most dramatic losses affect genes that are involved in metabolism but are not required for survival. Another general feature is the loss of most DNA repair systems and transcriptional regulatory mechanisms, indicating that there is reduced need for transcriptional regulation in a stable environment [29]. In *X. fastidiosa*, and to a lesser extent in *X. albilineans*, losses also affected genes involved in metabolism and transcriptional regulatory mechanisms (Figure 5). Metabolic capabilities essential for other habitats may have been lost in the genome reduction process coincidently with the adaptation of *X. fastidiosa *and *X. albilineans *to the nutrient-poor xylem environment. For *X. fastidiosa*, genome erosion has been extreme. For example, *X. fastidiosa *retained only one transcriptional sigma factor gene and one outer membrane efflux protein *tolC *gene, and it lost all genes involved in synthesis of the flagellar apparatus. This extreme erosion allowed *X. fastidiosa *to save energy (synthesis and operation of the flagella confer a growth disadvantage of about 2%; [30]).

In obligate animal symbionts and pathogens, the process of genome shrinkage might have taken place in two separate stages [29]. A massive gene loss must have occurred soon after the establishment of the obligate symbiosis, probably by means of large deletions that eliminated a series of contiguous genes. The large DNA region containing the flagellar genes was probably lost by *X. fastidiosa *during a similar stage. The accumulation of mobile elements, representing a source of chromosomal rearrangements and gene inactivation, seems to have an important role in this first stage. A similar process is likely responsible for the limited genome erosion of *X. oryzae *pv. *oryzae*, which possesses a very high number of insertion sequences (IS) covering 20% of the genome [31]. During the second stage of genome reduction in obligate animal symbionts and pathogens, genome shrinkage seems to have mostly occurred through a process of gradual gene loss, scattered along the genome. Such losses seem to follow a pattern that starts with the inactivation of a gene (pseudogenization) by single-nucleotide mutations, and continues with a rapid reduction in length until the original gene is completely eroded [29,32]. A similar process is likely responsible for the genome erosions of *X. fastidiosa *and *X. albilineans *(Additional files 2 and 3). Furthermore, the coding density of *X. fastidiosa *strain Temecula1 is significantly smaller than that of xanthomonads probably because of the degradation of ancestral genes. In *X. fastidiosa *strain 9a5c, the number of short annotated CDSs is considerably higher than in other *Xanthomonadaceae *(Table 1), although the functionality of these shortened CDSs, which may result from the degradation of ancestral genes, is questionable.

Obligate animal symbionts and pathogens display rapid evolution and have highly biased nucleotide base compositions with elevated frequencies of adenine and thymine (A+T) (reviewed in [29]). *X. fastidiosa *also displays rapid evolution (note that the length of the branch separating *X. fastidiosa *from the ancestor common to *X. albilineans *and *X. fastidiosa *is much longer, Figure 3) and has a high A+T content in comparison with other *Xanthomonadaceae *(Table 1). Furthermore, the GC skew pattern of the chromosome of *X. fastidiosa *has very high amplitude and contains a high number of diagram distortions (Figure 2). A similar atypical GC skew pattern was observed for the chromosome of a *Buchnera aphidicola *strain [33]. This latter atypical GC skew coincides with the loss of genes involved in the replication restart process (*recA *and *priA*) and may be explained by a higher frequency of cytosine deaminations [34]. The loss of DNA repair genes *recX*, *dinG *and *dinP *may explain, similarly, the very high GC skew of *X. fastidiosa*. It may also explain the more extensive genome erosion of *X. fastidiosa*, compared to *X. albilineans *and *X. oryzae *pv. *oryzae*. Alternatively, the most important factor affecting genome erosion of *X. fastidiosa *may reflect the insect-associated lifestyle specific to this *Xanthomonadaceae *[26].

The GC skew pattern of the *X. albilineans *chromosome contains a lower number of distortions and has a significantly higher amplitude than the GC skew pattern of other *Xanthomonas *species (Figure 2), indicating that no recent events of recombination have occurred in *X. albilineans*. Furthermore, the synteny between the chromosomes of *X. albilineans *strain GPE PC73 and *X. axonopodis *pv. *vesicatoria *strain 85-10 also indicated that recombination events were limited during the speciation of *X. albilineans *(Additional file 1). The limited recombination of the chromosome of *X. albilineans*, its limited erosion, its high G+C content and its low number of IS elements may indicate that a distinctive process was responsible for the reductive genome evolution of this pathogen.

We propose a unique mechanism of genome erosion involving the unique toxin albicidin produced by *X. albilineans*. Albicidin is a potent DNA gyrase inhibitor with 50% inhibitory concentrations (40 to 50 nM) lower than those of most quinolones [9]. DNA gyrase inhibitors block the religation of cleaved DNA intermediate during gyrase catalysis, resulting in lethal double-stranded DNA breaks [9,35]. In the presence of subinhibitory doses of DNA gyrase inhibitors, the SOS response mediates survival of the bacteria by allowing DNA replication to continue past breaks that would normally block it. In exchange for this survival advantage, there is an increased mutation rate because the polymerases that perform the repair are prone to error [36,37]. Several studies showed that subinhibitory doses of quinolones result in an increased mutation rate in *Escherichia coli*, *Staphylococcus aureus*, *Pseudomonas aeruginosa *and *Mycobacterium tuberculosis *[35,38-40]. *X. albilineans *has two genes conferring resistance to albicidin: an albicidin efflux pump gene that is present in the albicidin biosynthesis gene cluster XALB1 [20,41] and an albicidin-resistant DNA gyrase A gene elsewhere on the chromosome. This albicidin-resistant DNA gyrase A is unique to *X. albilineans *[42]. It contains a unique insertion of 43 amino-acids length close to the albicidin binding site. Production of albicidin in ancestral bacteria that possessed both the albicidin biosynthesis gene cluster and a DNA gyrase A sensitive to albicidin may have induced genome erosion. In these ancestral bacteria, most of the albicidin molecules were secreted by the albicidin efflux pump. Occasionally, molecules of albicidin that were not secreted most likely had the same effect as subinhibitory doses of quinolones: the SOS response was induced, thus resulting in DNA repair, recombination and mutagenesis. Successive and cumulative effect of albicidin at each replication cycle eventually resulted in genome erosion. The genome erosion induced by albicidin was likely arrested by evolution of the albicidin-resistant DNA gyrase A.

Acquisition of the albicidin biosynthesis genes by the ancestor of *X. albilineans *conferred a selective advantage because of the potent antibiotic activity of albicidin. The DNA damage caused by albicidin may rapidly have induced the mutation of DNA gyrase A gene and thus stopped the process of genome erosion, possibly explaining the distinctive genomic characteristics of *X. albilineans*. Albicidin inhibits the growth of *X. axonopodis *pv.* vesicatoria *(data not shown), suggesting that the DNA gyrase A of the ancestral *Xanthomonas *was sensitive to albicidin. Transfer of the albicidin biosynthesis gene cluster to *X. axonopodis *pv. *vesicatoria *led to production of functional albicidin [43], demonstrating that the production of albicidin per se is not lethal for a producer that possesses an albicidin-sensitive DNA gyrase A. No remains of the albicidin biosynthesis genes were found in the complete genome sequences of *X. fastidiosa*. Therefore, albicidin is most likely not responsible for genome erosion of *X. fastidiosa*. However, we cannot exclude the hypothesis that albicidin biosynthesis genes were lost during evolution of *X. fastidiosa*. For example, cluster XALB1 could have been lost concurrently with the flagellar biosynthesis gene cluster because these two gene clusters are close on the chromosome of *X. albilineans*.

## Conclusions

During their descent from a common ancestral parent, the two xylem-limited *Xanthomonadaceae *experienced a convergent reductive evolution. Adaptation to the nutrient-poor xylem elements and to the cloistered environmental niche of xylem vessels probably favoured this convergent evolution. Alternatively, the most important factor affecting genome erosion of *X. fastidiosa *and *X. albilineans *may reflect insect-associated lifestyles specific to these *Xanthomonadaceae. X. albilineans *and *X. fastidiosa *evolved differently: genome erosion has occurred to different extents and specific genes have been acquired independently by *X. albilineans *and *X. fastidiosa*. For example, *X. albilineans *has acquired a T3SS of the SPI-1 family that is mainly found in free-living animal pathogens and four NRPS gene clusters that are involved in the biosynthesis of albicidin and probably other unknown small molecules. The toxin albicidin may be responsible for the distinctive genome erosion of *X. albilineans*. Much progress has been recently made in understanding how *X. fastidiosa *spreads within the xylem vessels as well as the traits that contribute to its acquisition and transmission by sharpshooter vectors (For review, [26]). A similar in-depth functional analysis will be necessary to identify the genes that are required for *X. albilineans *to spread and succeed within sugarcane xylem vessels.

## Methods

### Bacterial strain

*X. albilineans *strain GPE PC73 was isolated from a diseased stalk of sugarcane cv. H63-1418 in Guadeloupe (France, [11]). Sequenced strain GPE PC73 is referred to as CFBP 7063 in the French Collection of Plant Pathogenic Bacteria ([44]http://www.angers.inra.fr/cfbp/).

### Genome sequencing, assembly and finishing

The complete genome sequence of *X. albilineans *was determined using the whole-genome shotgun method. Three libraries (A, B, and C) were constructed; two of them were obtained after mechanical shearing of genomic DNA and cloning of generated 3 Kbp and 10 Kbp inserts into plasmids pcdna2,1 (Invitrogen) (A) and pCNS (B) (pSU18 derived), respectively. Larger DNA fragments of about 25 Kbp (generated after partial digestion with *Sau*3A) were introduced into plasmid pBeloBac11 to generate a BAC library (C). Plasmid DNAs were purified and end-sequenced (33792 clones for A, 10752 for B and 4800 for C) by dye-terminator chemistry with ABI3730 sequencers (Applied Biosystems, Foster City, USA) leading to an approximately 17-fold coverage. The Phred/Phrap/Consed software package ([45]http://www.phrap.com) was used for sequence assembly and quality assessment. A total of 2151 additional sequence reactions were necessary for gap closure and sequence polishing that consisted of random sequencing of subclones (for 1625 sequence reactions) supplemented with 145 sequences of PCR-products and 381 sequences of oligonucleotide-targeted regions. Final error estimation rate as computed by phred/phrap/consed was less than 0.04 errors per 10 Kbp. The sequences reported here have been deposited in the EMBL GenBank database, and accession numbers are FP565176, FP340279, FP340278 and FP340277 for the chromosome and for plasmids plasmI, plasmII and plasmIII, respectively.

### Gene prediction and annotation

Sequence analysis and annotation were performed using iANT (integrated ANnotation Tool; [46]) as described for *R. solanacearum *[47]. The probabilistic Markov model for coding regions used by the gene prediction software FrameD [48] was constructed with a set of CDS sequences obtained from the public databank Swiss-Prot as revealed by BLASTX analysis. The alternative matrices were built using genes first identified in ACURs (Alternative Codon Usage Regions) based on homology and taken from the *R. solanacearum *annotation process [47]. Predicted CDSs were reviewed individually by gene annotators for start codon assignment. The corresponding products were automatically annotated using a protocol based on HAMAP scan [49], InterPro domain annotation and BLASTP analysis. Results were individually expertized to generate the proposed annotations. Proteins were classified according to MultiFun classification [50]. The complete annotated genetic map, search tools (SRS, BLAST), annotation and process classification are available at http://iant.toulouse.inra.fr/X.albilineans[51].

### Phylogenetic analysis

A phylogenetic tree was constructed from MLSA, with the maximum likelihood method and GTR as substitution model (with I: 0.01 and G: 0.52). The seven loci chosen, *gyrB*, *groEL, recA, dnaK*, *efp*, *atpD *and *glnA*, are typically selected housekeeping genes located at the following positions of the *X. albilineans *chromosome: 0.004, 0.348, 1.369, 1.983, 2.245, 3.442, and 3.655 Mb from the origin of replication, respectively. The total length of the concatenated group of full length CDSs nucleotide sequences was 10417 bp-10686 bp. The tree obtained with the concatenated data set of the seven housekeeping genes was constructed with *B. pseudomallei *strain NCTC 10247 as outgroup. Multiple alignments of the nucleotide sequences of the 7 housekeeping genes (*gyrB, atpD, dnaK, efp, groEL, glnA, recA*) for the 11 taxons were performed using ClustalW (The nucleotide alignment is provided in Additional file 6). The phylogenetic tree was calculated with PHYML ([52,53]; http://atgc.lirmm.fr/phyml/; version 2.4.4).

### OrthoMCL analysis

OrthoMCL clustering analyses were performed using the following parameters: P-value Cut-off = 1 × 10^-5^; Percent Identity Cut-off = 0; Percent Match Cut-off = 80; MCL Inflation = 1.5; Maximum Weight = 316. We modified OrthoMCL analysis by inactivating the filter query sequence during the BLASTP pre-process. All CDSs of *X. axonopodis *pv. *vesicatoria *strain 80-15 listed in Additional file 1 were assessed as having a best BLASTP hit within sequences belonging to *X. albilineans*, *X. fastidiosa *or *S. maltophilia*. Best BLASTP hit analyses were performed with database UniProt by excluding all accessions from the xanthomonads using expectation value lower than 1 × 10^-5^.

## Abbreviations

ACURs: alternative codon usage regions; CBD: cellulose binding domain; CDSs: protein-coding sequences; Hrp: hypersensitive response and pathogenicity; IS: insertion sequences; MLSA: multilocus sequence analysing; NRPSs: nonribosomal peptide synthetases; PSL: long polyserine linker; rpf: regulation of pathogenicity factors; SPI-1: *Salmonella *pathogenicity island -1; T3SS: Type III secretion system; TCSTS: two component signal transduction system.

## Authors' contributions

IP and MR contributed to manual annotation of the genome, analysed the data, drafted part of the manuscript and coordinated the project. VB, AC, SM, BS (Segurens) performed sequencing of the genome. SC (Carrere) and JG performed automatic annotation of the genome and OrthoMCL analysis. RK and SC (Cociancich) contributed to manual annotation of the genome and drafted part of the manuscript. CM, VV and MA conceived the study and revised the manuscript. AD, M-A J, EL, SP, BS (Szurek) contributed to manual annotation of the genome and revised the manuscript. PR conceived the study, contributed to manual annotation of the genome and drafted part of the manuscript. All authors read and approved the final manuscript.

## Supplementary Material

Additional file 1 **List and individual analysis of the 2816 ancestral genes identified in the genome of *X. axonopodis *pv. *vesicatoria *strain 85-10**. List and individual analysis of the 2816 CDSs of *X. axonopodis *pv. *vesicatoria *strain 85-10 identified by OrthoMCL and Best hit BLAST analyses as conserved in *X. albilineans *strain GPE PC73, *X. fastidiosa *strain 9a5c, *S. maltophilia *strain R551-3 or *S. maltophilia *strain K279a. Click here for file

Additional file 2 **List of ancestral genes potentially lost by pseudogenization and short deletions in *X. fastidiosa***. Analysis of lost ancestral genes that are present on the chromosome of *X. axonopodis *pv. *vesicatoria *between the orthologs of two ancestral genes that are contiguous and conserved in *X. fastidiosa*. Click here for file

Additional file 3 **List of ancestral genes potentially lost by pseudogenization and short deletions in *X. albilineans***. Analysis of lost ancestral genes that are present on the chromosome of *X. axonopodis *pv. *vesicatoria *between the orthologs of two ancestral genes that are contiguous and conserved in *X. albilineans*. Click here for file

Additional file 4 **List and description of the 11 genes unique to *X. albilineans *and *X. fastidiosa***. Summary of BLAST analyses results of the 11 genes unique to *X. albilineans *and *X. fastidiosa*. Click here for file

Additional file 5 **Comparison of the 5' end of 16S RNA of eight *Xanthomonadaceae***. Alignment of the 5' end of 16S RNA of the following strains: XAC = *Xanthomonas axonopodis *pv. *citri *str. 306, XOO = *Xanthomonas oryzae *pv. *oryzae *str. MAFF 311018, XCV = *Xanthomonas axonopodis *pv. *vesicatoria *str. 85-10, XCC = *Xanthomonas campestris *pv. *campestris *str. ATCC 33913, SMA = *Stenotrophomonas maltophilia *str. R551-3, XAL = *Xanthomonas albilineans *str. GPE PC73, XYL_9a5c = *Xylella fastidiosa *str. 9a5c and XYL_Tem = *Xylella fastidiosa *str. Temecula1. The yellow-highlighted region is specific to *X. albilineans *and *X. fastidiosa*. Click here for file

Additional file 6 **Alignment obtained with ClustalW of the concatenated sequences of the housekeeping genes *gyrB*, *atpD*, *dnaK*, *efp*, *groEL*, *glnA*, and *recA *of nine *Xanthomonadaceae***. Alignment obtained with ClustalW of the concatenated sequences of the housekeeping genes *gyrB*, *atpD*, *dnaK*, *efp*, *groEL*, *glnA*, and *recA *of X. albiline (*X. albilineans *str. GPE PC73), StenoK279a (*S. maltophilia *str. K279a), StenoR551 (*S. maltophilia *str. R551-3), Vesicatori (*X. axonopodis *pv. *vesicatoria *str. 85-10), Citri (*X. axonopodis *pv. *citri *str. 306), Oryzae (*X. oryzae *pv. *oryzae *str. MAFF 311018), Campestris (*X. campestris *pv. *campestris *str. ATCC 33913), Xyl9a5C (*X. fastidiosa *str. 9a5c), XylTemecul (*X. fastidiosa *str. Temecula1), Burkholder (*B. pseudomallei *str. NCTC 10247) and Ralstonia (*R. solanacearum *str. GMI1000). This alignment was not modified manually. Click here for file
