# Supplementary material for: The complete genome sequence of Xanthomonas albilineans provides new insights into the reductive genome evolution of the xylem-limited Xanthomonadaceae
Source: BMC Genomics. 2009 Dec 17;10:616. doi: 10.1186/1471-2164-10-616 (PMC2810307; doi:10.1186/1471-2164-10-616)
Supplement: Additional file 4 — List and description of the 11 genes unique to X. albilineans and X. fastidiosa. Summary of BLAST analyses results of the 11 genes unique to X. albilineans and X. fastidiosa. [file 1471-2164-10-616-S4.doc]

**Summary of blast analyses results of the 11 genes unique to *X. albilineans* and *X. fastidiosa***

| Accession in  *X. albilineans*  strain  GPE PC73 | Orthologs in  *X. fastidiosa* strain 9a5c | Orthologs in *X. fastidiosa* strain Temecula1 | Annotation in *X. albilineans* strain GPE PC73 | Closest ortholog (excluding *X. fastidiosa*) |
| --- | --- | --- | --- | --- |
| XALc_0169 | XF2272  I= 585/757 (77%)  E = 0 | PD1308  I= 585/757 (77%)  E = 0 | 5-methyltetrahydropteroyl  triglutamate--homocysteine methyltransferase (*metE*) | *Mesorhizobium* sp. BNC1  Meso_2701  I= 466/764 (60%)  E = 0  *Stenotrophomonas maltophilia* R551-3  [Smal_2042](http://www.ncbi.nlm.nih.gov/sites/entrez?db=gene&cmd=search&term=6476220&RID=3YHP5X0R01S&log$=geneexplicitprot&blast_rank=2)  I= 89/346 (25%)  E = 1e-24  *Xanthomonas axonopodis* pv. *citri* str. 306  XAC0336  I= 95/346 (27%)  E = 7e-25 |
| XALc_1080 | XF0156  I= 170/267 (63%)  E = 4e-98 | PD0125  I= 171/267 (64%)  E = 8e-100 | Cystein protease | *Pseudomonas syringae* pv. *oryzae* str. 1_6  I= 131/269 (48%)  E = 1e-68 |
| XALc_1235 | XF2110  I = 65/108 (60%)  E = 2e-34  XF1710  I= 64/108 (59%)  E = 5e-34 | PD0954  I = 66/108 (61%)  E = 1e-35  PD1087  I= 64/108 (59%)  E = 2e-34 | Hypothetical protein | *Acinetobacter* sp. ATCC 27244  HMPREF0023_2498  I= 64/139 (46%)  E = 2e-29 |
| XALc_1437 | XF0411  I= 428/564 (75%)  E = 0.0 | PD1663  I = 425/564 (75%)  E = 0.0 | ABC transporter | *Agrobacterium radiobacter* K84  [Arad_8260](http://www.ncbi.nlm.nih.gov/sites/entrez?db=gene&cmd=search&term=7365901&RID=3YKYRHC1013&log$=geneexplicitprot&blast_rank=5)  I= 408/590 (69%)  E = 0.0 |
| XALc_2293 | XF0878  I= 145/235 (61%)  E = 1e-81 | PD1802  I= 146/236 (61%)  E = 2e-82 | Polysaccharide deacetylase | *Acidithiobacillus ferrooxidans* ATCC 53993  Lferr_0405  I=140/234 (59%)  E = 7e-72 |
| XALc_2294 | XF0879  I= 182/357 (50%)  E = 2e-91 | PD1801  I= 180/357 (50%)  E = 1e-90 | Glycosyl transferase | *Acidithiobacillus ferrooxidans* ATCC 53993  [Lferr_0406](http://www.ncbi.nlm.nih.gov/sites/entrez?db=gene&cmd=search&term=6876358&RID=3YTMSWE1013&log$=geneexplicitprot&blast_rank=5)  I = 141/355 (39%)  E = 1e-62 |
| XALc_2937 | XF1854  I= 171/537 (31%)  E = 2e-60 | PD0949  I= 172/534 (32%)  E = 3e-64  PD0948  I= 170/532 (31%)  E = 7e-62 | Hypothetical protein | Not present in any other species |
| XALc_2981 | XF0813  I= 119/225 (52%)  E = 8e-42 | PD1854  I = 119/225 (52%)  E = 7e-42 | Hypothetical protein | *Ralstonia solanacearum* GMI1000  [RSp0031](http://www.ncbi.nlm.nih.gov/sites/entrez?db=gene&cmd=search&term=1222579&RID=3YUZTZ7H01S&log$=geneexplicitprot&blast_rank=5)  I= 92/200 (46%)  E = 6e-26 |
| XALc_2987 | XF2392  I = 133/205 (64%)  E = 2e-76 | PD1408  I= 77/135 (57%)  E= 6e-41 | Hydrolase | *Pseudomonas syringae* pv. *tomato* str. DC3000  [PSPTOA0030](http://www.ncbi.nlm.nih.gov/sites/entrez?db=gene&cmd=search&term=1187320&RID=3YV74CPN013&log$=geneexplicitprot&blast_rank=3)  I= 115/201 (57%)  E = 3e-65 |
| XALc_3169 | XF2747  I= 230/316 (72%)  E = 5e-136 | PD2085  I= 233/316 (73%)  E = 6e-138 | Hypothetical protein | *Pseudomonas aeruginosa* PAO1  PA2730  I= 279/313 (89%)  E = 6e-162 |
| XALq_3207 | XF1657  I= 97/189 (51%)  E = 1e-49 | PD1006  I= 98/189 (51%)  E = 3e-50 | Cell filamentation protein | *Bartonella grahamii* as4aup  Bgr_p00180  I= 111/209 (53%)  E = 1e-63 |

I: amino acid identity with the corresponding *X. albilineans* accession; E: expected value obtained for the Blast analyses against the corresponding *X. albilineans* accession.
