# Supplementary material for: The complete genome sequence of Xanthomonas albilineans provides new insights into the reductive genome evolution of the xylem-limited Xanthomonadaceae
Source: BMC Genomics. 2009 Dec 17;10:616. doi: 10.1186/1471-2164-10-616 (PMC2810307; doi:10.1186/1471-2164-10-616)
Supplement: Additional file 5 — Comparison of the 5' end of 16S RNA of eight Xanthomonadaceae. Alignment of the 5' end of 16S RNA of the following strains: XAC = Xanthomonas axonopodis pv. citri str. 306, XOO = Xanthomonas oryzae pv. oryzae str. MAFF 311018, XCV = Xanthomonas axonopodis pv. vesicatoria str. 85-10, XCC = Xanthomonas campestris pv. campestris str. ATCC 33913, SMA = Stenotrophomonas maltophilia str. R551-3, XAL = Xanthomonas albilineans str. GPE PC73, XYL_9a5c = Xylella fastidiosa str. 9a5c and XYL_Tem = Xylella fastidiosa str. Temecula1. The yellow-highlighted region is specific to X. albilineans and X. fastidiosa. [file 1471-2164-10-616-S5.doc]

XAC ---TAAGTGAAGAGTTTGATCCTGGCTCAGAGTGAACGCTGGCGGCAGGCCTAACACATG

XOO TTTTAAGTGAAGAGTTTGATCCTGGCTCAGAGTGAACGCTGGCGGCAGGCCTAACACATG

XCV -----AGTGAAGAGTTTGATCCTGGCTCAGAGTGAACGCTGGCGGCAGGCCTAACACATG

XCC ---TAAGTGAAGAGTTTGATCCTGGCTCAGAGTGAACGCTGGCGGCAGGCCTAACACATG

SMA ----------AGAGTTTGATCCTGGCTCAGAGTGAACGCTGGCGGTAGGCCTAACACATG

XAL ------------------------------AGTGAACGCTGGCGGCAGGCCTAACACATG

XYL_9a5c ---TAAGTGAAGAGTTTGATCCTGGCTCAGAGTGAACGCTGGCGGCAGGCCTAACACATG

XYL_Tem ---TAAGTGAAGAGTTTGATCCTGGCTCAGAGTGAACGCTGGCGGCAGGCCTAACACATG

*************** **************

XAC CAAGTCGAACGGCAGCACAGTAAGAGCTTGCTCTTATGGGTGGCGAGTGGCGGACGGGTG

XOO CAAGTCGAACGGCAGCACAGTAAGAGCTTGCTCTTATGGGTGGCGAGTGGCGGACGGGTG

XCV CAAGTCGAACGGCAGCACAGTAAGAGCTTGCTCTTATGGGTGGCGAGTGGCGGACGGGTG

XCC CAAGTCGAACGGCAGCACAGTAAGAGCTTGCTCTTATGGGTGGCGAGTGGCGGACGGGTG

SMA CAAGTCGAACGGCAGCACAGTAAGAGCTTGCTCTTACGGGTGGCGAGTGGCGGACGGGTG

XAL CAAGTCGAACGGCAGCACAGTGGTAGC--AATACCATGGGTGGCGAGTGGCGGACGGGTG

XYL_9a5c CAAGTCGGACGGCAGCACGTTGGTAGT--AATACCATGGGTGGCGAGTGGCGGACGGGTG

XYL_Tem CAAGTCGGACGGCAGCACATTGGTAGT--AATACCATGGGTGGCGAGTGGCGGACGGGTG

******* ********** * ** * * ***********************

XAC AGGAATACATCGGAATCTACTCTTTCGTGGGGGATAACGTAGGGAAACTTACGCTAATAC

XOO AGGAATACATCGGAATCTACTCTTTCGTGGGGGATAACGTAGGGAAACTTACGCTAATAC

XCV AGGAATACATCGGAATCTACTCTTTCGTGGGGGATAACGTAGGGAAACTTACGCTAATAC

XCC AGGAATACATCGGAATCTACTCTTTCGTGGGGGATAACGTAGGGAAACTTACGCTAATAC

SMA AGGAATACATCGGAATCTACTTTTTCGTGGGGGATAACGTAGGGAAACTTACGCTAATAC

XAL AGGAATACATCGGAATCTACCTTTTCGTGGGGGATAACGTAGGGAAACTTACGCTAATAC

XYL_9a5c AGGAATACATCGGAATCTACCTTATCGTGGGGGATAACGTAGGGAAACTTACGCTAATAC

XYL_Tem AGGAATACATCGGAATCTACCTTATCGTGGGGGACAACGTAGGGAAACTTACGCTAATAC

******************** * ********** *************************

**Additionnal file 5:** Alignment of the 5’ end of 16S RNA of the following strains: XAC = *Xanthomonas axonopodis* pv*. citri* str. 306, XOO = *Xanthomonas oryzae* pv. *oryzae* str. MAFF 311018, XCV = *Xanthomonas axonopodis* pv. *vesicatoria* str. 85-10, XCC = *Xanthomonas campestris* pv. *campestris* str. ATCC 33913, SMA = *Stenotrophomonas maltophilia* str. R551-3, XAL = *Xanthomonas albilineans* str. GPE PC73, XYL_9a5c = *Xylella fastidiosa* str. 9a5c and XYL_Tem = *Xylella fastidiosa* str. Temecula1. The yellow-highlighted region is specific to *X. albilineans* and *X. fastidiosa*.
