# Supplementary material for: The complete genome sequence of Xanthomonas albilineans provides new insights into the reductive genome evolution of the xylem-limited Xanthomonadaceae
Source: BMC Genomics. 2009 Dec 17;10:616. doi: 10.1186/1471-2164-10-616 (PMC2810307; doi:10.1186/1471-2164-10-616)
Supplement: Additional file 6 — Alignment obtained with ClustalW of the concatenated sequences of the housekeeping genes gyrB, atpD, dnaK, efp, groEL, glnA, and recA of nine Xanthomonadaceae. Alignment obtained with ClustalW of the concatenated sequences of the housekeeping genes gyrB, atpD, dnaK, efp, groEL, glnA, and recA of X. albiline (X. albilineans str. GPE PC73), StenoK279a (S. maltophilia str. K279a), StenoR551 (S. maltophilia str. R551-3), Vesicatori (X. axonopodis pv. vesicatoria str. 85-10), Citri (X. axonopodis pv. citri str. 306), Oryzae (X. oryzae pv. oryzae str. MAFF 311018), Campestris (X. campestris pv. campestris str. ATCC 33913), Xyl9a5C (X. fastidiosa str. 9a5c), XylTemecul (X. fastidiosa str. Temecula1), Burkholder (B. pseudomallei str. NCTC 10247) and Ralstonia (R. solanacearum str. GMI1000). This alignment was not modified manually. [file 1471-2164-10-616-S6.doc]

**Additional file 6**: Alignment obtained with ClustalW of the concatenated sequences of the housekeeping genes *gyrB*, *atpD*, *dnaK*, *efp*, *groEL*, *glnA*, and *recA* of X.albiline (*X. albilineans* str. GPE PC73), StenoK279a (*S. maltophilia* str. K279a), StenoR551 (*S. maltophilia* str. R551-3), Vesicatori (*X. axonopodis* pv. *vesicatoria* str. 85-10), Citri (*X. axonopodis* pv. *citri* str. 306), Oryzae (*X. oryzae* pv. *oryzae* str. MAFF 311018), Campestris (*X. campestris* pv. *campestris* str. ATCC 33913), Xyl9a5C (*X. fastidiosa* str. 9a5c), XylTemecul (*X. fastidiosa* str. Temecula1), Burkholder (*B. pseudomallei* str. NCTC 10247) and Ralstonia (*R. solanacearum* str. GMI1000*)*.

This alignment was not modified manually.

....|....| ....|....| ....|....| ....|....| ....|....| ....|....|

5 15 25 35 45 55

X.albiline ATGACCGACG AACAGAACAC CCACGCA--- ---AA---CA ACGGCAATTA CGACGCGAAT

StenoK279a ATGAGCGACG AACAGAACAC CCCGGCA--- ---AA---CA ACGGCAATTA CGACGCCAAC

StenoR551 ATGAGCGACG AACAGAACAC CCCGGCA--- ---AA---CA ACGGCAACTA CGACGCCAAC

Vesicatori ATGACCGACG AACAAAACAT CCCGCCA--- ---ACACCCA ACGGCACCTA CGACTCCAGC

Citri ATGACCGACG AACAAAACAC CCCGCCA--- ---ACACCCA ACGGCACCTA CGACTCCAGC

Oryzae ATGACCGACG AACAAAACAC CCCGCCA--- ---ACACCCA ACGGCACTTA CGACTCCAGC

Campestris ATGACCGACG AACAAACCAC ACCGCCA--- ---ACACCCA ACGGCACCTA CGACTCCAGC

Xyl9a5C ATGACCGAAA AGCAGAATAC CTCGCTGCTC ---AGTAATG GCGGCATATA TGACTCAAGT

XylTemecul ATGACCGAAA AGCAGAATAC ATCGCTGCTC ---AGTAATG GCGGCATATA TGACTCAAGT

Burkholder ATGACTGAAC AGCATAATTC GCAGCCCGAA ---------- --AATAGCTA CGGCGCGTCG

Ralstonia ATGACCGAAC AGCAGAAACC GCAATCCACA CCCGCCGAAA GCAGCAGCTA CGGCGCCGCC

....|....| ....|....| ....|....| ....|....| ....|....| ....|....|

65 75 85 95 105 115

X.albiline AGCATCACGG CCCTGGAAGG GTTGGAAGCG GTCCGCAAGC GTCCAGGGAT GTACATCGGT

StenoK279a AGCATTACCG CCCTGGAAGG CCTGGAGGCT GTCCGCAAGC GTCCCGGCAT GTACATCGGT

StenoR551 AGCATTACGG CCCTGGAAGG CCTGGAGGCT GTCCGCAAGC GCCCAGGCAT GTACATCGGT

Vesicatori AAGATCACCG TGCTGCGTGG CCTGGAGGCC GTCCGCAAGC GCCCCGGCAT GTATATCGGC

Citri AAGATCACCG TGCTGCGTGG CCTGGAAGCC GTCCGCAAGC GTCCCGGCAT GTATATCGGC

Oryzae AAGATCACCG TGCTGCGTGG CCTGGAAGCC GTCCGCAAGC GCCCCGGCAT GTACATCGGC

Campestris AAGATCACCG TGCTGCGTGG CCTCGAAGCC GTCCGCAAGC GCCCCGGAAT GTACATCGGC

Xyl9a5C AAAATCACAG TACTACGTGG ACTGGATGCT GTTCGCAAGC GTCCAGGGAT GTATATCGGA

XylTemecul AAAATCACAG TACTACGTGG ACTGGATGCT GTTCGCAAGC GTCCAGGGAT GTATATCGGA

Burkholder TCGATCCAGA TCCTCGAAGG CCTGGAAGCG GTGCGCAAGC GACCCGGGAT GTACATCGGC

Ralstonia TCGATCCAGA TCCTGGAAGG CCTGGAGGCG GTGCGCAAGC GGCCGGGCAT GTACATCGGC

....|....| ....|....| ....|....| ....|....| ....|....| ....|....|

125 135 145 155 165 175

X.albiline GACGTCCATG ACGGCACTGG CCTGCATCAC ATGGTGTTCG AGGTTGTCGA TAACTCCATC

StenoK279a GACGTGCATG ACGGCACCGG TCTGCACCAC ATGGTGTTCG AGGTCGTCGA CAACTCGATC

StenoR551 GACGTCCATG ACGGCACCGG CCTGCACCAC ATGGTGTTCG AGGTGGTCGA CAACTCCATC

Vesicatori GACGTCCATG ACGGCACCGG CCTGCATCAC ATGGTGTTCG AGGTGGTCGA CAACTCGGTC

Citri GACGTCCATG ACGGCACCGG CCTGCATCAC ATGGTGTTCG AGGTGGTCGA CAACTCGGTC

Oryzae GATGTCCATG ACGGCACCGG CCTGCATCAC ATGGTGTTCG AGGTGGTCGA CAACTCGGTC

Campestris GATGTGCACG ACGGCACCGG CCTGCACCAC ATGGTGTTCG AGGTGGTCGA CAATTCGGTC

Xyl9a5C GATGTCCATG ACGGTACAGG TTTGCACCAC ATGGTGTTTG AAGTTGTCGA TAACTCGGTG

XylTemecul GATGTCCATG ACGGTACAGG TTTGCACCAC ATGGTGTTTG AAGTTGTCGA TAACTCGGTG

Burkholder GACACGTCGG ACGGCACCGG TCTGCATCAC CTCGTGTTCG AGGTGCTCGA CAACTCGATC

Ralstonia GATACGTCGG ACGGCACCGG CCTGCACCAC CTCGTGTTCG AGGTGCTGGA CAACTCCATC

....|....| ....|....| ....|....| ....|....| ....|....| ....|....|

185 195 205 215 225 235

X.albiline GACGAGGCGC TGGCCGGTCA TGCGGACCAT GTCGCGGTTA CGATCCATGC CGATGGTTCG

StenoK279a GACGAAGCGC TGGCCGGCCA CGCCGACCAC GTTGCGGTGA CGATCCACGC CGACGGTTCG

StenoR551 GACGAAGCCC TCGCCGGCCA TGCCGACCAC GTGGCGGTGA CGATCCACGC CGACGGCTCG

Vesicatori GACGAAGCCC TGGCCGGGCA TGCGGACGAC ATCGTGGTGA AGATCCTGGC CGATGGCTCG

Citri GACGAAGCCC TTGCCGGGCA TGCCGACGAC ATCGTGGTAA AAATCCTGGC CGATGGCTCG

Oryzae GACGAAGCCC TGGCCGGGCA TGCCGACGAC ATCGTGGCGA AAATCCTGGT CGATGGCTCG

Campestris GACGAGGCCC TGGCGGGCCA CGCCGATGAC ATCGTGGTCA AGATCCACGT GGATGGCTCG

Xyl9a5C GATGAGGCAT TAGCTGGTCA TGCCGATAGC ATTCTTGTCA AGATCCATGT TGATGGGTCT

XylTemecul GATGAGGCAT TAGCTGGTCA TGCCGACAGC ATTCTTGTCA AGATCCATAT TGATGGGTCT

Burkholder GACGAGGCGC TCGCCGGCTA CTGCAACGAC ATCCATGTGA CCATACACGC GGACAATTCG

Ralstonia GACGAGGCGC TGGCGGGGCA TTGCACCGAG ATCCACGTCA CCATCCACAC CGACAACTCG

....|....| ....|....| ....|....| ....|....| ....|....| ....|....|

245 255 265 275 285 295

X.albiline GTGTCGGTAT CGGATAACGG ACGTGGCATT CCGGTCGGCA AGCA-TGCGC AGATGAGCAA

StenoK279a GTCTCGGTGT CCGACAACGG CCGTGGTATC CCGACTGGCA AGCA-CGAGC AGATGAGTGC

StenoR551 GTTTCGGTGT CCGACAACGG TCGCGGCATT CCGACGGGCA AGCA-CGAGC AGATGAGCGC

Vesicatori GTGGCGGTCT CCGACAACGG GCGCGGCGTA CCGGTCGACA TCCA-CAAG- -GAAGAAGGC

Citri GTGGCGGTCT CCGACAACGG GCGCGGCGTG CCGGTCGACA TCCA-CAAG- -GAAGAAGGC

Oryzae GTGGCGGTGT CAGACAACGG ACGCGGCGTG CCGGTCGACA TTCA-CAAG- -GAAGAAGGC

Campestris GTAGCGGTGT CGGACAACGG CCGCGGCGTT CCGGTGGACA TCCA-CAAG- -GAAGAAGGC

Xyl9a5C GTCTCTGTTT CGGATAACGG TCGAGGTATC CCAGTAGATA TTCA-TAAA- -GAAGAGGGT

XylTemecul GTCTCTGTTT CGGATAACGG TCGAGGTATC CCTGTAGATA TTCA-TAAA- -GAAGAGGGT

Burkholder ATCTCCGTGA CCGACAACGG CCGCGGCATC CCGACCGACG TCAAGCTCAA CGACAAGCAC

Ralstonia ATCTCCGTGA TCGACAACGG CCGCGGCATC CCGACCGGCA TCAAGTTCGA TGACAAGCAC

....|....| ....|....| ....|....| ....|....| ....|....| ....|....|

305 315 325 335 345 355

X.albiline GAAGCTCGAT CGCGAGGTCT CGGCGGCCGA AGTCGTGATG ACCGTCCTGC ATGCAGGCGG

StenoK279a CAAGCTCGGT CGCGAAGTGT CTGCAGCCGA AGTCGTCATG ACCGTCCTGC ACGCAGGCGG

StenoR551 CAAGCTCGGC CGCGAAGTGT CCGCTGCCGA AGTGGTGATG ACCGTCCTGC ACGCAGGCGG

Vesicatori ---------- ------GTCT CGGCGGCCGA GGTGATCCTC ACCGTGCTGC ACGCCGGCGG

Citri ---------- ------GTGT CGGCGGCCGA GGTGATCCTC ACCGTGCTCC ACGCCGGCGG

Oryzae ---------- ------GTGT CCGCGGCCGA GGTGATCCTC ACCGTGCTGC ATGCCGGCGG

Campestris ---------- ------GTTT CCGCAGCCGA GGTGATCCTC ACCGTGCTGC ACGCCGGCGG

Xyl9a5C ---------- ------GTTT CTGCCGCTGA AGTGATCCTG ACAGTTTTAC ATGCTGGTGG

XylTemecul ---------- ------GTTT CTGCCGCTGA AGTGATCCTG ACAGTTTTAC ATGCTGGTGG

Burkholder GAGCCG---- ---AAGCGCT CGGCCGCCGA AATCGTGATG ACCGAGCTGC ATGCGGGCGG

Ralstonia GAGCCG---- ---AAGCGCA GCGCGGCCGA GATCGTCATG ACCGAGCTGC ACGCCGGCGG

....|....| ....|....| ....|....| ....|....| ....|....| ....|....|

365 375 385 395 405 415

X.albiline TAAGTTCGAC GACAACAGCT ACAAGGTTTC TGGTGGCCTG CATGGCGTTG GTGTCAGTGT

StenoK279a CAAGTTCGAC GACAACAGCT ACAAGGTTTC GGGCGGCCTG CACGGCGTCG GCGTCAGCGT

StenoR551 CAAGTTCGAC GACAACAGCT ACAAGGTTTC CGGTGGCCTG CATGGCGTCG GCGTCAGCGT

Vesicatori CAAGTTCGAC GACAACAGCT ACAAGGTCTC CGGCGGCCTG CATGGCGTAG GCGTCTCGGT

Citri CAAGTTCGAC GACAACAGCT ACAAGGTCTC CGGCGGCCTG CACGGCGTTG GCGTCTCGGT

Oryzae CAAGTTCGAC GACAACAGCT ACAAGGTCTC CGGCGGCCTG CATGGCGTGG GCGTTTCGGT

Campestris CAAGTTCGAC GACAACAGCT ACAAAGTGTC CGGCGGCCTG CACGGCGTGG GCGTCTCGGT

Xyl9a5C TAAGTTCGAC GATAACAGCT ATAAGGTTTC TGGTGGTTTG CATGGGGTAG GAGTTTCAGT

XylTemecul TAAGTTCGAC GATAATAGCT ATAAGGTTTC TGGTGGTTTG CATGGGGTAG GAGTTTCAGT

Burkholder CAAGTTCGAC CAGAACAGCT ACAAGGTGTC GGGCGGCCTG CACGGCGTCG GCGTGTCGTG

Ralstonia CAAGTTCGAC CAGAACAGCT ACAAGGTGTC GGGCGGCCTG CACGGCGTGG GTGTGTCGTG

....|....| ....|....| ....|....| ....|....| ....|....| ....|....|

425 435 445 455 465 475

X.albiline GGTCAACGCG CTGTCGGAAA AGCTGCTGCT GGATATTTTC CAGGGCGGCT TCCATTACCA

StenoK279a GGTCAACGCG CTGTCGCAGA AGCTGCTGGT GGACGTGTTC CAGAACGGCT TCCACTACCA

StenoR551 GGTCAACGCG CTCTCGCAGA AGCTGCTGGT GGACGTGTTC CAGGGCGGTT TCCACTACCA

Vesicatori GGTCAACGCA TTGTCCGAGC ACTTGTGGCT GGATATCTGG CGCGACGGCT TCCACTACCA

Citri GGTCAACGCG TTGTCAGAGC ACCTGTGGCT GGATATCTGG CGCGACGGCT TCCACTACCA

Oryzae GGTCAACGCG CTGTCCGAGC ACCTGTGGCT GGACATCTGG CGCGATGGTT TCCACTACCA

Campestris GGTCAACGCG CTGTCCGAGC ACCTGTGGCT GGATATCTGG CGCGACGGCT TCCACTACCA

Xyl9a5C GGTCAACGCC CTTTCTGAGC GATTATGGCT TGATATTTGG CGTGATGGTT ACCACTACCA

XylTemecul GGTCAACGCC CTTTCTGAGC GATTATGGCT TGATATTTGG CGTGATGGTT ACCACTACCA

Burkholder CGTGAACGCG CTCTCGAGCT GGCTGCGCCT CATCGTGCGC CGCGATGGCA AGAAGCACTT

Ralstonia CGTGAACGGC CTGTCGAAGT GGCTCAAGCT GACGGTGCGC CGCGACGGCC GCGTGCACCA

....|....| ....|....| ....|....| ....|....| ....|....| ....|....|

485 495 505 515 525 535

X.albiline GCAGGAATAT AGCCACGGCG --CCGCCTTG GCGCCG---- --------C- ----------

StenoK279a GCAGGAATTC AGCAACGGCG --CCGCAGTG ACCGCG---- --------C- ----------

StenoR551 GCAGGAATTC AGCAACGGCG --CCGCGGTC ACCCCG---- --------C- ----------

Vesicatori GCAGGAATAC GCGCTGGGCG AACCGCAGTA CCCGCT---- --------C- ----------

Citri GCAGGAATAC GCGCTGGGCG AGCCGCAGTA CCCGCT---- --------C- ----------

Oryzae GCAGGAATAT GCGCTGGGCG AGCCGCAGTA CCCGCT---- --------C- ----------

Campestris GCAGGAATAT GCGCTGGGTG AGCCGCAGTA CCCGCT---- --------C- ----------

Xyl9a5C ACAAGAATAT GTTTTGGGTG AGCCGCAATA TCCTCT---- --------T- ----------

XylTemecul ACAAGAATAT GTTTTGGGTG AGCCGCAATA TCCTCT---- --------T- ----------

Burkholder CATGGAATTC CATCGCGGCA TCGCGCAGAA CCGCGTGCT- --------CG AGGAGCGCGA

Ralstonia CATGGAATTC GCGCGCGGCA TCCCGCAGAA CCGCCTGCTG GAGCCGGCCG AGGCGCCGGA

....|....| ....|....| ....|....| ....|....| ....|....| ....|....|

545 555 565 575 585 595

X.albiline ----TGAAGC A--------- ---------- ---GCTGGAA ACCAGCGCCA AGCGTGGTAC

StenoK279a ----TGGCCA A--------- ---------- ---GCTGGAA ACCACCACCA AGCGCGGCAC

StenoR551 ----TGGCAA A--------- ---------- ---GCTGGAG AGCACCACCA AGCGCGGTAC

Vesicatori ----AAGC-- A--------- ---------- ---GCTGGAA GCCTCGACCA AGCGCGGCAC

Citri ----AAGC-- A--------- ---------- ---GCTGGAA GCCTCGACCA AGCGCGGTAC

Oryzae ----AAGC-- A--------- ---------- ---GCTGGAA GCATCGACCA AGCGCGGCAC

Campestris ----AAGC-- A--------- ---------- ---GCTGGAA GCCTCGACCA AGCGCGGCAC

Xyl9a5C ----AAGC-- A--------- ---------- ---GTTAGGG GTGTCAGCGA AACGCGGTAC

XylTemecul ----AAGC-- A--------- ---------- ---GTTAGAG GTGTCAGCGA AACGCGGTAC

Burkholder CGGCGAGCAG GTC------T CGCCGATGCA GGTGATCGGC GAGACCGAGA ACCGCGGCAC

Ralstonia CGGCAAGACG GTCGAAGTGT CGCCGCTGCG CGTGTCGGGC ACCACCGACA AGCGCGGCAC

....|....| ....|....| ....|....| ....|....| ....|....| ....|....|

605 615 625 635 645 655

X.albiline CACGTTACGC TTCT-GGCCG TCGGTGAAGG CGTTCAATAA CAACGTCGAG TTCCACTACG

StenoK279a TACCGTGCGC TTCT-GGCCG TCCACCGTCG CCTTCCACGA CAACGTGGAG TTCCACTACG

StenoR551 CACGGTGCGC TTCT-GGCCG TCGACCGTGG CGTTCCACGA CAACGTGGAA TACCACTACG

Vesicatori CACGCTGCGC TTCA-AGCCG TC---CGTGG CCATCTTCAG CGATGTCGAG TTCCATTACG

Citri CACGCTGCGC TTCA-AGCCG TC---CGTGG CCATCTTCAG CGACGTCGAG TTCCATTACG

Oryzae CACGCTGCGC TTCA-AGCCG TC---CGCGG CCATCTTCAG TGACGTCGAG TTCCACTACG

Campestris CACGCTGCGC TTCA-AGCCG GC---GGTGG AGATCTTCAG CGACGTCGAG TTCCATTACG

Xyl9a5C TACATTACGT TTTA-AACCG GC---AAAAG AGATCTTCAG TGATGTAGAG TTTCATTACG

XylTemecul TACATTACGT TTTA-AACCG GC---AAAAG AGATCTTCAG TGATGTGGAG TTTCATTACG

Burkholder CGAAGTGCAC TTCATGGCCG AT----CCGA CGATCTTCGG CACCGTCGAA TACCACTACG

Ralstonia CGAAGTGCAC TTCCTGGCCG AC----GAAG AGATCTTCAC CAACGTCGAG TACCACTACG

....|....| ....|....| ....|....| ....|....| ....|....| ....|....|

665 675 685 695 705 715

X.albiline ACATCCTGGC CCGCCGCCTG CGTGAGCTGT CCTTCCTCAA CTCCGGGGTC AAGATCGTCC

StenoK279a ACATCCTGGC CCGCCGCCTG CGCGAACTGT CGTTCCTGAA CTCCGGCGTC AAGATCGTCC

StenoR551 ACATCCTGGC CCGTCGCCTG CGTGAGCTGT CGTTCCTGAA TTCCGGCGTC AAGATCGTGC

Vesicatori ACATCCTGGC GCGGCGCCTG CGCGAGCTGT CCTTCCTCAA TTCCGGCGTC AAGATCACCT

Citri ACATCCTGGC GCGGCGCCTG CGCGAGCTGT CCTTCCTCAA TTCTGGCGTC AAGATCACCT

Oryzae ACATCCTGGC GCGGCGCCTG CGTGAGCTGT CGTTCCTCAA TTCCGGCGTC AAGATCACCC

Campestris ACATCCTGGC GCGCCGCCTA CGCGAGCTTT CGTTCCTCAA CTCCGGCGTC AAGATTGCGC

Xyl9a5C AAAATCTTGC AAAGCGCCTC CGTGAGTTAT CCTTCCTCAA TTCCGGCCTT CAAGTTAGTT

XylTemecul AAAATCTTGC AAAGCGCCTC CGTGAGTTAT CCTTCCTCAA TTCCGGCCTT AAAGTTAGTT

Burkholder ACATTCTCGC GAAGCGCATC CGCGAGCTCT CGTTCCTGAA CAACGGCGTG CGCATCCGGC

Ralstonia AGATCCTCTC CAAGCGCATC CGCGAGCTCT CGTTCCTGAA CAACGGTGTG CACATCAAGC

....|....| ....|....| ....|....| ....|....| ....|....| ....|....|

725 735 745 755 765 775

X.albiline TAGCCGACGA ACGTGGGGAA GGCCGTCGCG ACGATTTCCA CTACGAAGGC GGCATCCGCA

StenoK279a TTGCCGACGA GCGTGGCGAC GGCCGTCGCG ATGACTTCCA CTACGAGGGT GGCATCCGCA

StenoR551 TGGCCGACGA GCGTGGTGAT GGCCGCCGCG ATGACTTCCA CTACGAAGGC GGTATTCGCA

Vesicatori TGATCGACGA GCGCGGCGAA GGCCGTCGCG ACGATTTCCA TTACGAAGGC GGCATCCGCA

Citri TGATCGACGA GCGCGGCGAA GGCCGTCGCG ACGATTTCCA TTACGAAGGC GGCATCCGCA

Oryzae TGATCGACGA GCGTGGCGAA GGCCGTCGCG ACGATTTCCA TTACGAAGGC GGCATCCGCA

Campestris TGATCGACGA GCGCGGCGAA GGCCGGCGCG ACGATTTCCA CTACGAAGGC GGCATTCGCA

Xyl9a5C TGATTGATGA ACGTGGTGAG GGTCGGCGTG ACGATTTTCA CTATGAAGGT GGTATTCGTA

XylTemecul TGATTGATGA ACGTGGTGAG GGTCGGCGTG ACGATTTTCA CTATGAAGGT GGTATTCGTA

Burkholder TGACCGACCT GCGCTCCG-- -GCAAGGAAG ACGATTTCGC GTTCGCGGGC GGCGTGAAGG

Ralstonia TGACCGACCA GCGCACCG-- -GCAAGGAAG AAGACTTCGC CTTCTCGGGC GGCGTGAAGG

....|....| ....|....| ....|....| ....|....| ....|....| ....|....|

785 795 805 815 825 835

X.albiline GCTTCGTCGA GCACTTGGCG CACCTGAAAA CCCCACTGCA TCCGAACGTG ATTTCGGTGA

StenoK279a GCTTCGTGGA GCATCTGGCC CAGCTGAAGA CGCCGCTGCA CCCGAACGTC ATTTCAGTCA

StenoR551 GCTTCGTCGA GCATCTGGCC CAGCTGAAGA CCCCGCTGCA CCCGAACGTC ATCTCGGTCA

Vesicatori GCTTCGTGGA GCATCTGGCG CAGTTGAAGT CGCCGCTGCA CCCGAATGTG ATCTCGGTGA

Citri GCTTCGTGGA GCATCTGGCG CAGCTGAAGT CGCCGCTGCA CCCGAATGTG ATCTCGGTGA

Oryzae GCTTCGTGGA GCATCTTGCC CAGCTGAAGT CGCCGCTGCA CCCGAACGTG ATCTCGGTGA

Campestris GCTTCGTGGA GCATCTGGCC CAGCTCAAGA CCCCGCTGCA CCCGAACGTG ATTTCGGTGA

Xyl9a5C GCTTTGTAGA ACATTTGGCT CAATTGAAGA CCCCGTTGCA TTCGAATGTA ATTTCGGTTA

XylTemecul GCTTTGTAGA ACATTTAGCT CAATTGAAGA CCCCATTGCA TCCGAATGTA ATTTCGGTCA

Burkholder GCTTCGTCGA GTACATCAAC AAGACGAAGA GCGTGCTGCA CCCGACGATC TTCCACATCA

Ralstonia GCTTCGTCGA GTACATCAAC AAGAACAAGA CCGTGCTGCA CCCGACCGTC TTCAACGCCA

....|....| ....|....| ....|....| ....|....| ....|....| ....|....|

845 855 865 875 885 895

X.albiline GCGGCGAACA TAACGGCATC ACCGTGGAAG TGGCGCTGCA GTGGACCGAC TCCTATCAGG

StenoK279a CCGGCGAGCA CAACGGCATC GTCGTGGATG TGGCGCTGCA GTGGACCGAC TCCTATCAGG

StenoR551 CCGGCGAGCA CAACAACATC GTGGTGGATG TGGCGCTGCA GTGGACCGAC TCCTATCAGG

Vesicatori CCGGCGAGCA CAACGGCATC ATGGTGGACG TGGCCCTGCA ATGGACCGAC GCCTACCAGG

Citri CCGGCGAGCA CAACGGCATC ATGGTGGACG TGGCCCTGCA ATGGACCGAC GCCTACCAGG

Oryzae CCGGCGAGCA CAATGGCATT GTGGTGGACG TGGCCCTGCA GTGGACCGAC GCCTACCAGG

Campestris CCGGCGAGCA CAACGGCATC GTGGTGGACG TGGCCCTGCA ATGGACCGAC GCCTACCAGG

Xyl9a5C CTGGTGAGCA CAACGGCATT GTTGTGGATG TTGCTTTACA GTGGACTGAT GCCTACCAAG

XylTemecul CAGGGGAGCA CAACGGCATT GTTGTGGATG CTGCTTTACA GTGGACTGAT GCCTACCAAG

Burkholder ACGGCGAGAA GGATAGCGTG GGCGTGGAAG TAGCGATGCA GTGGAACGAC AGCTACAACG

Ralstonia CGGGCGAGAA GGACGGCGTG GGCGTGGAAG TGTCGATGCA GTGGAACGAC GGCTTCAACG

....|....| ....|....| ....|....| ....|....| ....|....| ....|....|

905 915 925 935 945 955

X.albiline AGACGATGTA CTGTTTCACC AACAATATCC CGCAAAAAGA CGGTGGCACC CACCTGGCAG

StenoK279a AGACGATGTA CTGCTTCACC AACAACATTC CGCAGAAGGA CGGCGGTACC CACCTGGCCG

StenoR551 AGACGATGTA CTGCTTCACC AACAACATCC CGCAGAAGGA CGGTGGTACC CACCTCGCCG

Vesicatori AAACCATGTA CTGCTTCACC AACAATATTC CGCAGAAGGA CGGCGGCACC CACCTGGCCG

Citri AAACCATGTA CTGCTTCACC AACAACATCC CGCAGAAGGA CGGCGGCACC CACCTGGCCG

Oryzae AAACCATGTA CTGCTTCACC AACAACATCC CGCAGAAGGA TGGCGGCACC CACTTGGCCG

Campestris AAACCATGTA TTGCTTCACC AATAACATCC CGCAGAAGGA TGGCGGCACG CATCTGGCCG

Xyl9a5C AAACAATGTA TTGTTTTACA AATAACATTC CACAAAAAGA TGGTGGTACC CACCTTGCTG

XylTemecul AAACAATGTA TTGTTTTACA AATAACATTC CACAAAAAGA TGGCGGTACC CACCTTGCTG

Burkholder AAAACGTGCT GTGCTTCACG AACAACATCC CGCAGCGCGA CGGCGGCACG CACTTGACCG

Ralstonia AGCAGGTGCT CTGCTTCACC AACAACATCC CGCAGCGCGA CGGCGGCACC CACCTGACCG

....|....| ....|....| ....|....| ....|....| ....|....| ....|....|

965 975 985 995 1005 1015

X.albiline GTTTCCGCGG TGCCCTGACC CGCGTGCTCA ACAACTACAT CGAGCAGAAT GGCATTGCCA

StenoK279a GTTTCCGCGG TGCGCTGACC CGTGTGCTCA ACAACTACAT CGAGCAGAAC GGCATCGCCA

StenoR551 GCTTCCGCGG CGCGCTGACC CGCGTGCTCA ACAACTACAT CGAGCAGAAC GGCATCGCCA

Vesicatori GTTTCCGTGC GGCGCTGACG CGCGTGCTCA GCACCTACAT CGAACAGAAC GGCATCGCCA

Citri GCTTCCGTGC GGCGCTGACG CGCGTGCTCA GCACCTACAT CGAACAGAAC GGCATCGCCA

Oryzae GCTTCCGCGC CGCGCTGACG CGCGTGCTGG GCACCTACAT CGAGCAGAAC GGCATCGCCA

Campestris GCTTCCGCGG CGCGTTGACG CGCGTGCTGA GCAACTACAT CGAGCAGAAC GGCATCGCCA

Xyl9a5C GCTTCCGTGC TGCATTGACT CGTACCTTGG GTAATTACAT TGAGCAGAAT GGGATTGCTA

XylTemecul GCTTCCGTGC TGCATTGACT CGTACGTTGG GTAATTACAT TGAGCAGAAT GGGGTTGCTA

Burkholder GCCTGCGCGC GGCGATGACG CGCGTGATCA ACAAGTACAT CGCCGACAAC GAGATCGCGA

Ralstonia GCCTGCGCGC CGCGATGACG CGCGTCATCA ACAAGTACAT CGCCGACAAC GAGATCGCCA

....|....| ....|....| ....|....| ....|....| ....|....| ....|....|

1025 1035 1045 1055 1065 1075

X.albiline AGCAGGCCAA GATCAACCTG ACCGGCGATG ACATGCGCGA AGGCATGATT GCGGTGCTCT

StenoK279a AGCAGGCCAA GATCAACCTG ACCGGCGACG ACATGCGAGA AGGCATGATC GCGGTGCTGT

StenoR551 AGCAGGCCAA GATCAACCTG ACCGGCGACG ACATGCGTGA AGGCATGATC GCGGTGCTGT

Vesicatori AGCAGGCCAA GGTCGCGCTG ACCGGCGATG ACATGCGCGA AGGCATGATC GCGGTGCTTT

Citri AGCAGGCCAA GGTCGCGCTG ACCGGCGACG ACATGCGCGA AGGCATGATC GCGGTGCTTT

Oryzae AGCAGGCCAA GGTTGCGCTG ACCGGTGACG ACATGCGCGA AGGCATGATC GCAGTGCTGT

Campestris AGCAGGCAAA GATCACCCTG ACCGGCGACG ACATGCGCGA AGGCATGATC GCAGTGCTGT

Xyl9a5C GGCAAGCGAA GATCACTTTT TCTGGTGATG ATATGCGTGA AGGTATGATT GCCGTGTTAT

XylTemecul GGCAAGCGAA GATCACTTTT TCTGGTGATG ATATGCGTGA AGGTATGATT GCCGTGTTAT

Burkholder AGAAGGCGAA GGTCGAGACG ACCGGCGACG ACATGCGCGA AGGGCTTTCG TGCGTGCTCT

Ralstonia AGAAGGCCAA GGTCGAAACC TCCGGCGATG ACATGCGCGA AGGCCTGACC TGCGTGCTGT

....|....| ....|....| ....|....| ....|....| ....|....| ....|....|

1085 1095 1105 1115 1125 1135

X.albiline CGGTGAAGGT GCCGGACCCG AGTTTTTCCA GCCAGACCAA GGAAAAGCTG GTCAGTTCCG

StenoK279a CGGTGAAGGT GCCGGACCCG AGCTTCTCCA GCCAGACCAA GGAAAAGCTG GTCAGCTCCG

StenoR551 CGGTGAAGGT GCCCGATCCC AGTTTCTCCA GCCAGACCAA GGAAAAGCTG GTCAGCTCCG

Vesicatori CGGTCAAGGT GCCCGACCCC AGCTTCTCTT CGCAGACCAA GGAAAAGCTG GTCAGCTCGG

Citri CGGTCAAGGT GCCCGACCCC AGCTTCTCTT CGCAGACCAA GGAAAAGCTG GTCAGTTCGG

Oryzae CGGTCAAAGT GCCCGACCCC AGCTTCTCCT CGCAGACCAA GGAAAAGCTG GTCAGCTCGG

Campestris CGGTGAAGGT GCCCGACCCC AGCTTCTCCT CGCAGACCAA GGAAAAACTG GTCAGCTCGG

Xyl9a5C CGGTAAAAGT CCCTGAACCT AGTTTTTCTT CACAAACCAA GGAAAAGTTG GTGAGCTCGG

XylTemecul CGGTAAAAGT CCCTGAACCT AGTTTTTCTT CCCAAACCAA GGAAAAGTTG GTGAGCTCCG

Burkholder CCGTGAAGGT GCCGGAGCCG AAGTTCAGCT CGCAGACGAA GGACAAGCTG GTTTCGTCCG

Ralstonia CGGTGAAGGT GCCCGAGCCC AAGTTCAGCT CGCAGACCAA GGACAAGCTC GTTTCGTCCG

....|....| ....|....| ....|....| ....|....| ....|....| ....|....|

1145 1155 1165 1175 1185 1195

X.albiline ACGTGCGCCC GGCGGTGGAA AATGCGTTCG GTGCCCGTCT GGAAGAGTTC CTGCAGGAGA

StenoK279a ACGTGCGTCC GGCGGTGGAA AACGCCTTCG GTGCGCGCCT GGAAGAGTTC CTGCAGGAGA

StenoR551 ACGTGCGCCC CGCCGTGGAA AACGCCTTCG GTGCGCGGCT GGAAGAGTTC CTGCAGGAAA

Vesicatori ATGTGCGCCC GGCGGTGGAA AACGCCTTCG GTGCGCGCCT GCAGGAGTTC CTGCAGGAGA

Citri ATGTGCGCCC GGCGGTGGAA AACGCCTTCG GTGCGCGCCT GCAGGAGTTC CTGCAGGAGA

Oryzae ACGTGCGCCC GGCGGTGGAA AACGCCTTCG GTGCGCGCTT GCAGGAGTTC CTGCAAGAGA

Campestris ACGTGCGCCC GGCGGTGGAA AATGCCTTCG GTGCGCGCCT GCAGGAATTC CTGCAGGAAA

Xyl9a5C ATGTCAAGCC GGCAGTGGAA GCTACCTTTG GCTTGCGTTT GGAGGAATTC TTGCAAGAGA

XylTemecul ATGTCAAGCC GGCAGTGGAA GCTACCTTTG GCTTGCGTTT GGAGGAATTC TTACAAGAGA

Burkholder AAGTGCGCGC GCCGGTGGAG GAAGTGGTGG CGAAGGCGCT CGAGGAGTTT CTGCTCGAAA

Ralstonia AAGTGCGCCT GCCGGTGGAA GAAGTCGTGG CCAAGGCGCT GGCGGACTTC CTGCTGGAAA

....|....| ....|....| ....|....| ....|....| ....|....| ....|....|

1205 1215 1225 1235 1245 1255

X.albiline ACCCCAACGA GGCCAAGGCC ATCGCCGGCA AGATCGTCGA TGCCGCGCGC GCACGCGAGG

StenoK279a ACCCGAACGA AGCCAAGGCG ATCGCCGGCA AGATCGTCGA CGCCGCGCGC GCACGTGAAG

StenoR551 ACCCGAACGA AGCCAAGGCG ATCGCCGGCA AGATCGTCGA CGCCGCGCGT GCCCGTGAAG

Vesicatori ACCCGAACGA AGCCAAGGCC ATCACCGGCA AGATCGTCGA TGCAGCGCGC GCGCGCGAAG

Citri ACCCGAACGA AGCCAAGGCC ATCACCGGCA AGATCGTCGA TGCCGCGCGT GCGCGCGAAG

Oryzae ACCCGAACGA AGCCAAGGCC ATCACTGGCA AGATCGTCGA TGCCGCGCGT GCGCGCGAAG

Campestris ATCCGAACGA AGCCAAGGCC ATCACCGGCA AGATCGTCGA TGCCGCCCGT GCCCGTGAAG

Xyl9a5C ATCCTAACGA AGCACGTGCA ATTGCTGGAA AGATTGTCGA TGCTGCTCGT GCTCGCGAGG

XylTemecul ATCCTAACGA AGCACGTGCA ATTGCTGGAA AGATTGTCGA TGCTGCTCGT GCTCGCGAGG

Burkholder CGCCGAACGA CGCGAAGATC ATCTGCGGCA AGATCGTCGA AGCGGCGCGC GCGCGCGATG

Ralstonia CGCCCAACGA CGCCAAGATC ATCTGCGGCA AGATCGTTGA AGCCGCCCGC GCCCGCGAAG

....|....| ....|....| ....|....| ....|....| ....|....| ....|....|

1265 1275 1285 1295 1305 1315

X.albiline CCGCGCGCAA GGCGCGCGAC CTGACCCGCC GCAAGGGCGC CTTGGATATC GCGGGCCTGC

StenoK279a CGGCGCGAAA GGCCCGCGAC CTGACCCGCC GCAAGGGCGC GCTGGATATC GCCGGCCTGC

StenoR551 CCGCGCGCAA GGCCCGCGAC CTGACCCGCC GCAAGGGTGC GCTGGACATC GCCGGCCTGC

Vesicatori CCGCCCGCAA GGCGCGCGAC CTGACCCGCC GCAAGGGCGC GTTGGACATC GCCGGCCTGC

Citri CGGCGCGCAA GGCGCGCGAC CTCACCCGCC GCAAGGGTGC GCTGGACATC GCAGGCCTGC

Oryzae CCGCGCGCAA GGCGCGCGAC CTCACCCGCC GCAAGGGCGC GCTGGATATC GCCGGCCTGC

Campestris CCGCCCGCAA GGCGCGCGAT CTGACCCGCC GCAAGGGCGC GCTGGATATC GCCGGCCTGC

Xyl9a5C CTGCTCGAAA AGCCCGTGAT CTAACCCGTC GAAAAGGTGT GCTTGATATC GCCGGTTTAC

XylTemecul CTGCTCGCAA AGCCCGCGAT TTAACCCGTC GAAAAGGTGT GCTTGATATC GCCGGTTTGC

Burkholder CCGCGCGCAA GGCGCGCGAG ATGACGCGCC GCAAGGGCGT GCTCGACGGC GTGGGCCTGC

Ralstonia CCGCCCGCAA GGCCCGCGAG ATGACGCGCC GCAAGGGCGT GCTCGACGGC ATGGGCCTGC

....|....| ....|....| ....|....| ....|....| ....|....| ....|....|

1325 1335 1345 1355 1365 1375

X.albiline CGGGCAAGCT CGCCGACTGC CAGGAAAAAG ATCCAGCACT ATCTGAATTG TTCATTGTTG

StenoK279a CGGGCAAGCT GGCCGACTGC CAGGAAAAGG ATCCGGCGCT GTCCGAACTG TTCATCGTCG

StenoR551 CGGGCAAGCT GGCCGACTGC CAGGAAAAGG ATCCGGCGCT GTCCGAACTG TTCATCGTCG

Vesicatori CCGGCAAGCT CGCCGATTGC CAGGAAAAGG ACCCGGCGCT GTCGGAACTG TTCATCGTCG

Citri CCGGCAAGCT GGCCGACTGC CAGGAAAAGG ATCCGGCACT GTCGGAACTG TTCATCGTCG

Oryzae CGGGCAAGCT GGCCGACTGC CAGGAAAAAG ATCCGGCGCT GTCCGAACTC TTCATTGTCG

Campestris CGGGCAAGCT GGCCGATTGC CAGGAAAAGG ATCCGGCGCT GTCCGAGCTG TTCATCGTCG

Xyl9a5C CGGGTAAGTT GGCAGATTGC CAAGAGAAAG ACCCAGCGAT GTCAGAATTG TTTATCGTCG

XylTemecul CAGGTAAGTT GGCAGATTGC CAAGAGAAAG ACCCAGCGAT GTCAGAATTG TTTATCGTCG

Burkholder CGGGCAAGCT CGCGGACTGC CAGGAGAAGG ACCCGGCGAA GTCGGAAATC TACATTGTCG

Ralstonia CCGGCAAGCT GGCCGACTGC CAGGAGAAAG ACCCGGCGCT GTCCGAACTG TTCATCGTCG

....|....| ....|....| ....|....| ....|....| ....|....| ....|....|

1385 1395 1405 1415 1425 1435

X.albiline AGGGTGATTC AGCGGGCGGC TCGGCCAAGC AGGGTCGGAA TCGCAGGAAT CAGGCGGTAC

StenoK279a AGGGTGACTC GGCAGGTGGC TCGGCCAAGC AGGGCCGCAA CCGCAAGAAC CAGGCGGTGC

StenoR551 AGGGTGACTC GGCAGGTGGC TCGGCCAAGC AGGGCCGCAA CCGCAAGAAC CAGGCGGTGC

Vesicatori AGGGTGACTC GGCGGGCGGT TCGGCCAAGC AGGGACGCAA CCGCAAGAAC CAGGCCGTGC

Citri AGGGTGACTC GGCAGGTGGC TCGGCCAAGC AGGGCCGCAA CCGCAAGAAC CAGGCGGTGC

Oryzae AGGGTGACTC GGCAGGTGGC TCGGCCAAGC AGGGACGCAA CCGCAAGAAC CAGGCGGTGC

Campestris AGGGTGACTC GGCAGGTGGC TCGGCCAAGC AGGGCCGCAA CCGCAAGAAC CAGGCGGTGC

Xyl9a5C AAGGGGATTC TGCTGGTGGT TCTGCAAAGC AGGGACGTAA CCGAAAAAAT CAGGCAGTAT

XylTemecul AAGGGGATTC TGCTGGTGGT TCTGCAAAGC AGGGGCGTAA CCGAAAAAAT CAGGCAGTAT

Burkholder AGGGCGACTC GGCGGGCGGC TCAGCCAAGC AGGGGCGCGA CCGCAAGTTC CAGGCGATCC

Ralstonia AGGGTGACTC CGCAGGCGGC TCGGCCAAGC AGGGCCGCGA CCGCAAGTTC CAGGCGATCC

....|....| ....|....| ....|....| ....|....| ....|....| ....|....|

1445 1455 1465 1475 1485 1495

X.albiline TGCCCTTGCG CGGCAAGATC CTCAACGTCG AGCGTGCCCG TTTCGATCGC ATGCTGGCCT

StenoK279a TGCCGCTGCG CGGCAAGATC CTCAACGTGG AACGCGCGCG CTTCGACCGC ATGCTGTCCT

StenoR551 TGCCGCTGCG CGGCAAGATC CTCAACGTGG AACGCGCGCG CTTTGACCGC ATGCTGGCCT

Vesicatori TGCCGCTGCG CGGCAAGATC CTCAATGTCG AACGTGCCCG CTTCGACCGC ATGCTGGCCT

Citri TGCCGCTGCG CGGCAAGATC CTCAACGTGG AACGCGCCCG CTTCGACCGC ATGCTGGCCT

Oryzae TGCCGTTGCG CGGCAAGATC CTCAACGTGG AACGTGCGCG CTTCGACCGC ATGCTGGCGT

Campestris TGCCGCTGCG CGGCAAGATC CTCAACGTCG AACGCGCGCG CTTCGATCGC ATGCTGGCCT

Xyl9a5C TGCCGCTCAG GGGTAAGATT CTTAATGTTG AACGTGCACG CTTCGACCGT ATGCTCTCTA

XylTemecul TGCCGCTCAG GGGCAAGATT CTTAACGTTG AACGTGCACG ATTCGACCGT ATGCTCTCTA

Burkholder TGCCGCTGCG CGGCAAGGTG CTGAACGTCG AGAAGGCGCG CTACGACAAG CTGCTGTCGT

Ralstonia TGCCGCTCAA GGGCAAGATC CTGAACGTGG AACGCGCGCG CTTCGACAAG ATGCTCTCCA

....|....| ....|....| ....|....| ....|....| ....|....| ....|....|

1505 1515 1525 1535 1545 1555

X.albiline CCGACCAGGT CGGGACGCTG ATCACCGCAT TGGGTACCGG CATCGGTCGC GACGAGTACA

StenoK279a CCGACCAGGT CGGCACGCTG ATCACGGCGC TGGGCACCGG CATCGGCCGA GACGAGTACA

StenoR551 CTGACCAGGT CGGTACGCTG ATCACCGCGC TGGGCACCGG CATTGGCCGC GACGAGTACA

Vesicatori CCGACCAGGT CGGCACGCTG ATCACTGCGC TGGGCACCGG CATCGGTCGC GACGAGTACA

Citri CCGACCAGGT CGGCACGCTG ATCACCGCGC TCGGTACCGG CATCGGCCGC GACGAGTACA

Oryzae CGGACCAGGT CGGCACGCTG ATCACCGCAC TGGGCACCGG CATCGGCCGC GACGAGTACA

Campestris CCGACCAGGT GGGCACGCTG ATCACCGCGC TGGGCACCGG CATCGGCCGC GACGAGTACA

Xyl9a5C GTGCTGAGGT TGGCACATTG ATCACGGCCC TTGGGACAGG CATCGGTAAG GACGAATATA

XylTemecul GTGCTGAGGT TGGCACATTG ATCACGGCCC TTGGGACAGG CATCGGTAAG GACGAATATA

Burkholder CCGAGCAGAT CGTCACGCTC GTGACCGCGC TCGGCTGCGG GATCGGCAAG GACGACTACA

Ralstonia GCCAGGAAGT GCTCACGCTC ATCACCGCCA TGGGCACCGG CATCGGCAAG GACGACTACA

....|....| ....|....| ....|....| ....|....| ....|....| ....|....|

1565 1575 1585 1595 1605 1615

X.albiline ACCCGGACAA ACTGCGTTAC CACCGCATCA TCATCATGAC CGACGCCGAC GTCGACGGCG

StenoK279a ACCCGGACAA GCTGCGTTAC CACAAGATCA TCATCATGAC CGACGCCGAC GTCGACGGCG

StenoR551 ACCCGGACAA GCTGCGCTAC CACAAGATCA TCATCATGAC CGACGCCGAC GTCGACGGCG

Vesicatori ACCCGGACAA GCTGCGCTAC CACCGCATCA TCCTGATGAC CGACGCCGAC GTGGACGGCT

Citri ACCCGGACAA GCTGCGCTAC CACCGCATCA TCCTGATGAC CGACGCCGAC GTCGACGGCT

Oryzae ACCCGGACAA ACTGCGCTAC CACCGCATCA TCCTGATGAC CGACGCCGAC GTCGACGGCT

Campestris ACCCGGACAA GCTGCGCTAC CACCGCATCA TCCTGATGAC CGACGCCGAC GTGGACGGCT

Xyl9a5C ACCCAGATAA GCTACGTTAC CATCGCATCA TCATTATGAC CGATGCTGAT GTGGATGGTT

XylTemecul ACCCAGATAA GCTACGTTAC CATCGCGTCA TCATTATGAC CGATGCGGAT GTGGATGGTT

Burkholder ACCTCGACAA GCTGCGCTAC CACCGGATCA TCATCATGAC CGATGCGGAC GTGGACGGCG

Ralstonia ACCTCGACAA GCTGCGCTAC CACCGCATCA TCATCATGAC CGACGCGGAC GTGGACGGCT

....|....| ....|....| ....|....| ....|....| ....|....| ....|....|

1625 1635 1645 1655 1665 1675

X.albiline CCCACATCCG CACCTTGCTG CTGACCTTCT TCTACCGGCA GATGCCTGAG CTGATCGAAC

StenoK279a CCCACATCCG CACCCTGCTG CTGACGTTCT TCTACCGTCA GATGCCGGAG CTGATCGAGC

StenoR551 CCCACATCCG CACGCTGCTG CTGACGTTCT TCTACCGTCA GATGCCGGAG CTGATCGAGC

Vesicatori CGCACATCCG CACCCTGCTG CTGACCTTCT TCTACCGGCA GATGCCGGAG TTGATCGAGC

Citri CGCACATCCG CACCCTGCTG CTGACCTTCT TCTACCGGCA GATGCCGGAG TTGATCGAGC

Oryzae CGCACATCCG TACCCTGTTG CTGACCTTCT TCTACCGGCA GATGCCGGAG CTGATCGAGC

Campestris CGCACATCCG CACGCTGCTG CTCACGTTCT TCTACCGGCA GATGCCGGAG CTGATCGAGC

Xyl9a5C CGCATATCCG TACTTTATTG CTGACGTTTT TCTACAGGCA AATGCCAGAA TTGATTGAAC

XylTemecul CGCATATCCG TACTTTATTG CTGACGTTTT TTTACAGGCA AATGCCAGAA TTGATTGAAC

Burkholder CGCACATCCG CACGCTGCTG CTGACGTTCT TCTACCGGCA GATGCCGGAG ATGATCGAGC

Ralstonia CGCACATCCG CACGCTGCTG CTGACGTTCT TCTACCGCCA GATGCCCGAG ATCATCGAGC

....|....| ....|....| ....|....| ....|....| ....|....| ....|....|

1685 1695 1705 1715 1725 1735

X.albiline GCGGCTACGT CTACATCGGC TTGCCCCCGC TCTACCGGCT CAAGCAAGGT AAGCAGGAGC

StenoK279a GCGGTTACGT CTACATCGGC CTGCCGCCGC TGTACAAGAT CAAGCAGGGC AAGCAGGAGC

StenoR551 GCGGTTACGT CTACATCGGC CTGCCGCCGC TGTACAAGAT CAAGCAGGGC AAGCAGGAGC

Vesicatori GCGGCTACAT CTACATCGGC CTGCCGCCGC TGTACAAGCT CAAGCAGGGC AAGAGCGAGC

Citri GCGGCTACAT CTACATCGGC CTGCCGCCGC TGTACAAGCT CAAGCAGGGC AAGAGCGAGC

Oryzae GGGGCTACAT CTACATCGGC CTGCCGCCGC TGTACAAGCT CAAGCAGGGC AAGAGCGAGC

Campestris GCGGCTACAT CTACATCGGC CTGCCGCCGC TGTACAAGCT CAAGCAGGGC AAGAGCGAGC

Xyl9a5C GTGGCCATAT CTATATTGGC TTGCCGCCCT TGTACAAGCT AAAGCAGGGT AAGAGTGAGC

XylTemecul GTGGCCATAT CTATATTGGC TTGCCGCCCT TGTACAAGCT AAAGCAGGGT AAGAGTGAGC

Burkholder GCGGCTACGT GTACATCGCG CAGCCGCCGC TTTACAAGGT GAAGGCGGGC CGCGACGAGC

Ralstonia GCGGCCACGT GTACATCGCC CAGCCGCCGC TGTACAAGAT CAAGCACGGC AAGGAAGAGC

....|....| ....|....| ....|....| ....|....| ....|....| ....|....|

1745 1755 1765 1775 1785 1795

X.albiline TTTACCTGAA GGACGACAAC GCGCTCAACG TCTATCTGGC CAGCAGCGCG GTCGAGGGTG

StenoK279a TGTACCTGAA GGACGACCCG GCGCTGGACA GCTACCTGGC CAGCAGCGCG GTGGAAAACG

StenoR551 TGTACCTGAA GGACGACCCG GCGCTGGACA GCTACCTGGC CAGCAGTGCG GTGGAAAACG

Vesicatori TGTATCTGAA GGACGACGCG GCGCTCAACG CCTACCTGGC TAGCAATGCG GTCGAGGGCG

Citri TGTATCTGAA GGACGACGCG GCGCTCAACG CCTACCTGGC CAGCAATGCG GTCGAAGGTG

Oryzae TGTATCTGAA GGACGATGCA GCGCTCAACG CCTACCTGGC CAGCAATGCG GTCGAAGGTG

Campestris TGTATCTCAA GGACGACGCC GCGCTCAACG CCTACCTGGC CAGCAGCGCG GTCGAAGGTG

Xyl9a5C TGTACTTAAA GGACGATATC GCATTAAATG TTTATTTAGC GAATAGCGCG GTTGAAGGAG

XylTemecul TGTACTTAAA GGACGATATC GCATTGAATG TTTATTTGGC GAATAGCGCG GTTGAAGGAG

Burkholder GCTACCTGAA GGACGATACC GAGCTGAACG CGCACATGCT GCGCCTCGCG CTGCAGGGCT

Ralstonia GCTACATCAA GGACGACGTC GAGATGGCCG CGTACCTGAT GCGCCAGGCC CTCGACACCG

....|....| ....|....| ....|....| ....|....| ....|....| ....|....|

1805 1815 1825 1835 1845 1855

X.albiline CGGCGTTGAT CCCGGCGAGC GGCGAGCCGC CGATCACCGG CGCGGCACTG GAGAAGTTGT

StenoK279a CTGCGCTGGT GCCGGCCACC GGCGAGCCCG GCATCGAAGG CCTTGCGCTG GAGAAGCTGC

StenoR551 CTGCGCTGGT GCCGGCCACC GGTGAACCGG GCATCGAAGG CCTGGCGCTG GAAAAGCTGC

Vesicatori CGGCGCTGAT TCCGGCCACC GACGAACCGC CGATCACCGG CGAAGCGCTG GAGAAATTGT

Citri CGGCGCTGAT TCCGGCCACC GACGAGCCAC CGATCACCGG CGAAGCGCTG GAGAAATTGT

Oryzae CGGCGCTGAT TCCGGCCACC GACGAGCCGC CGATCACCGG CGAAGCGCTG GAAAAACTGC

Campestris CAGCGCTGAT CCCGGCCAGC GACGAGCCGC CGATCACCGG CGAGGCGCTG GAAAAACTGC

Xyl9a5C CGCAATTACT CCCCGCTGAA GGTGAACCGC CCATTGAGGG TCTGGCATTG GAGAAATTGC

XylTemecul CGCAATTACT CCCCGCTGAA GGTGAGCCGC CCATTGAGGG TCTTGCATTG GAGAAATTGC

Burkholder CGGAGCTCGT GCCGACCGAG AACGGCACGC CGATCTCGGG CGACGCGCTC GGCGAGCTCG

Ralstonia CCATCCTGGT GCGCGCCG-- -ACGGCACCG AGATCGCCAG CGACGCGCTG GCCGAGCTGG

....|....| ....|....| ....|....| ....|....| ....|....| ....|....|

1865 1875 1885 1895 1905 1915

X.albiline TGCTGCTGTT CGCCAGCGCC AATGAAACCG TGGTACGCAA CGCCCATCGC TACGATCCTG

StenoK279a TGCTGGCCTA TGCAGCAGCG CTGGATTCGA TCGAGCGCAA CGCACATCGC TACGACCGCA

StenoR551 TGCTGACCTA CGCCGCCGCG CAGGATTCGA TCGAGCGCAA CGCACACCGC TACGACCGCA

Vesicatori TGATGCTGTT CACCAGCGCC AACGAAGCGA TCGCGCGCAA CGCACACCGC TACGACCCGG

Citri TGATGCTGTT CACCAGCGCC AACGAAGCGA TCGCGCGCAA CGCCCACCGC TACGACCCGG

Oryzae TGCTGCTGTT CACCAGCGCC AACGAAGCGA TTGCGCGCAC CGCGCACCGC TACGACCCGG

Campestris TGCTGTTGTT CGCTGGCGCC AAGGAGGCCA TTGCCCGCAA TGCCCACCGC TACGACCCGG

Xyl9a5C TGATTACATA TATCGCTGCC AAAGATACTA TCGCGCGTCA TTCGCATCGC TATGATTCTC

XylTemecul TGATTACATA TATCGCCGCA AAAGATACTA TCGTGCGTCA TTCGCATCGC TATGATTCTC

Burkholder CGCGCTCGTA TCTGCTCGCG CGGGGCGTGG TGGAGCGGTT GAGCCGCCTG TACGACCCGG

Ralstonia CGCGGCAGTA CCAGTTCTCG CGCGCCGTGA TCGAGCGACT GTCGCGCGTG ATCGATGCGG

....|....| ....|....| ....|....| ....|....| ....|....| ....|....|

1925 1935 1945 1955 1965 1975

X.albiline CACTGCTGAC CGCATTGATC GACTTGCCGC CACTGGACGT GGCCCAGCTC GAAGCGGAGG

StenoK279a ACCTGCTTGA AGCGCTGGTC GATTTCGTGC CGATGGACCT GGAAAGCCTG CGCGCTGCCG

StenoR551 ACGTGCTCGA AGCGCTGGTC GACTTCGTCC CGATGGACAT GGACAGCCTG CGCAATGCCG

Vesicatori CCCTGCTCAC CGCGCTGATC GACCTGCCGC CACTGGATGT GGAAAAACTG CAGGCCGAAG

Citri CCCTGCTCAC CGCGCTGATC GACCTGCCGC CACTGGATGT GGAAAAACTG CAGGCCGAAG

Oryzae CTTTGCTGAC CGCATTGATC GACCTGCCGC CGCTGGATGT GGAAACACTG CAGGCCGAAG

Campestris CGCTGTTGAC CGCGTTGATC GATCTGCCGC CGCTGGACGT GGTGCAGCTG CAGGCCGAAG

Xyl9a5C GCCTCCTGGA AGCACTGCTT GAATTTACCC CCTTGGATTC GACTTGCTTT CAATCCTACA

XylTemecul GCCTCCTGGA AGCACTGCTT GAATTTACTC CCTTGGATTC GACTTGCTTT CAATCCTACA

Burkholder CCGCGCTCGA GGCGGTCATG GACGGTGTCG CGATCGATCT GTCGAGCGAG GCATCGACGG

Ralstonia ATGCGCTGCG CGCCATTGCC GAAGGCGTGG CGCTGGACCT GTCCAGCGAA GCGGGCGCCG

....|....| ....|....| ....|....| ....|....| ....|....| ....|....|

1985 1995 2005 2015 2025 2035

X.albiline GCGAACGGCA TCCAAGCCTG GATGCGCTGC AAATGGTTCT CAATCGTGGT AACCTCGGTT

StenoK279a GCGAAGGCGA ---GGGCCTG GATGCACTGG CCAAGCGCCT CAACCAGGGC AGCCTGGGCA

StenoR551 GCGAAGGCGA ---AGGCCTG GATGCATTGG CCAAGCGCCT CAACCAGGGC AGCCTGGGCA

Vesicatori GCGACCAGCA TCCGACCCTG GATGCACTGC AGGCAGTGCT CAACCGCGGC ACCCTGGGTA

Citri GCGACCAGCA TCCGACTTTG GATGCGCTGC AGGCCGTGCT CAATCGCGGC ACCCTGGGCA

Oryzae GCAATCAGCA TCCGGCCCTG GATGCGCTGC AGGCAGTGCT CAATCGCGGT ACCCTGGGCA

Campestris GCGACGTGCA TCCCACGCTG GATGCGCTGC AGGCAGTGCT CAATCGCGGC ACCCTGGGCA

Xyl9a5C GTGATAGCGA ---ACATCTA CAGTCGCTGA TGGCATTGCT GAACCAGTCC AGCTTGGGTG

XylTemecul GTGATAGCGA ---ACATCTA CAATCGCTGA TGGCATTGCT GAACCAGTCT AGCTTGGGTG

Burkholder AGGCGTCGGC GAAGGCGCTG GCCGCCATGC TATCCGA--- -CGATTCGAA GACCGAAGTG

Ralstonia AAGCCAGCGC CAAGGCGCTC AAGGCGCGGC TGCTGGAAAT GCAGGGCAAC GCCAGCAGCG

....|....| ....|....| ....|....| ....|....| ....|....| ....|....|

2045 2055 2065 2075 2085 2095

X.albiline C-----GGCC CGCTACACCT TGCGCTTTCA GCCCGC-CAA CGAGCAGCGT TCGGC---CA

StenoK279a G-----CCCG CGTTTCACGC TGGAACTGCA GGAGGC-CAA CGACGAGCGC CCGGC---CG

StenoR551 G-----CCCG CGCTTCACCC TGGAGCTGCA GGAAGC-CAA CGAGCAGCGC CCGGC---AG

Vesicatori C-----CGCG CGCTACCAGT TGCGCTTCGA CCCCGG-CAG CGAGAACGCG CCTGC---CA

Citri C-----CGCG CGCTATCAGT TGCGCTTCGA CCCGGG-CAG CGACAACGCG CCTGC---CA

Oryzae C-----AGCG CGCTACCAGT TGCGCTTCGA CCCGGC-CAC CGAGAACGCG CCTGC---CA

Campestris C-----GGCG CGCTATCACC TGCGCTTCGA CCCGGC-CAC CGACAGTGCC GCCGC---CT

Xyl9a5C C-----TCCG CGTTATATTC TGAAGATGCA GTTCCC-TGA TGAGCAACAT CCTGC---CG

XylTemecul C-----TCCG CGTTATATTC TTAAGATGCA GTTTCC-TGA TGAACAACAT TCTGC---CG

Burkholder C-----GCGT CGTGCCGGCT TACG-----A TGCGGTACG- CGAGCAGCGC TCG-----CT

Ralstonia CCAACGGTGG CGCGACGGCG GATGCGTTCA TGCAGTACGA CGAGAAGCAC GAGAAGTACC

....|....| ....|....| ....|....| ....|....| ....|....| ....|....|

2105 2115 2125 2135 2145 2155

X.albiline CCTTGCTGTT GGTGCGTCGC CACATGGGCG AGGAAATGAC CCAGGTGGTG CCGATGGCGG

StenoK279a CTGTGCTGGT GACCCGTCGC CACATGGGCG AACAGCACAT CCAGGTGCTG CCGATGTCGG

StenoR551 CCGTGCTGGT GACCCGCCGC CACATGGGCG AAGAGCTGAT CCAGGTGCTG CCGCTGTCGG

Vesicatori CCCTGGTGGC CATCCGCCGC CACATGGGCG AAGAGTTCAC CCAGGTGCTG CCGATGGGTG

Citri CGTTGGTGGC CATCCGCCGC CACATGGGCG AAGAGTTCAC CCAGGTGCTG CCGATGGGGG

Oryzae CGCTGGTGGC GATCCGCCGT CATATGGGCG AAGAATTCAC CCAGGTGCTG CCGATGGGGG

Campestris CGCTGGTGTC GGTGCGCAAG CACATGGGCG AGGAATTCAC CCAGGTGCTG CCGATGGGCG

Xyl9a5C CCTTGTTGGC GACCCGCTAT CACATGGGTG AGGAATTGAC TCAGGTGTTG CAATTATCAG

XylTemecul CCTTGTTGGC GACCCGCTAT CACATGGGTG AGGAATTGAC TCAGGTGTTG CAATTATCAG

Burkholder GCGCGTGGAG CGCACGC--- -ATCACGGCA ACGTGCGCGT ATCGGTGATC GACGAGGAAT

Ralstonia GCGTGATGGT CGTGCGCCGC CAGCACGGCA ACCAACGCCT ATCGCACATC GACGCCGACT

....|....| ....|....| ....|....| ....|....| ....|....| ....|....|

2165 2175 2185 2195 2205 2215

X.albiline TGTTCGAAAG CGGCGAACTG CGGGCGTTGC GCGAGGTCGC GCTGGCGCTG CACGGTCTGG

StenoK279a CCTTCGAAAG CGGCGAACTG CGCGCAATCC ACCAGGCATC GAAGCTGCTG CACGGTCTGG

StenoR551 CCTTCGAAAG CGGTGAACTG CGTGCGATCC ACCAGGCGTC CTCGCTGCTG CATGGCTTGG

Vesicatori CGTTCGAAAG CGGCGAGCTG CGTCCGCTGC GTGAGGTCTC GCTGGCCTTG CACGACCTGG

Citri CCTTCGAAAG CGGCGAGCTG CGCCCGCTGC GTGAGGTGTC GCTGGCCCTG CACGACCTGG

Oryzae CGTTCGAAAG CGGCGAGCTG CGTCCGCTGC GTGAGGTATC GCTGGCCCTG CATGATCTGG

Campestris CGTTCGAAAG TGGTGAGCTG CGTCCGCTGC GCGAGGTTGC GCTGGCCCTG CATGGCTTGG

Xyl9a5C CATTAGAGAC TGGCGAGTTA CGTCCACTCT ATGAAGCGGC CAAGTTGCTG CACGGCTTAA

XylTemecul CATTAGAGAC TGGTGAGTTA CGTCCACTCT ATGAAGCGGC CAAGTTGCTA CATGGCTTAA

Burkholder TCCAGCACAC GGCCGATTAT CAGCAGCTCG TGAACACCGC GAATACGTTC AAGGGCTTGA

Ralstonia TCGTGGCCGG CGCCGACTAC GCCACGCTGT CGCAGACTGC GCAGACCTTC CAGGGGTTGA

....|....| ....|....| ....|....| ....|....| ....|....| ....|....|

2225 2235 2245 2255 2265 2275

X.albiline TCCGCGAGGG CGCGCAGATC GTGCGCGG-- -------CAA CAAGACC--- CAAGCCATCT

StenoK279a TCCGCGAAGG CGCGACCATT TCCCGCGG-- -------TGC CAAGTCG--- ATCGAAGTCG

StenoR551 TCCGCGAAGG CGCGATCATT TCACGTGG-- -------CGC CAAGTCG--- ATCGAAGTGG

Vesicatori TGCGCGAAGG TGCCCAGATC GTGCGCGG-- -------CAA CAAGAGC--- CACCCGATCA

Citri TGCGCGAAGG CGCCCAGATC GTCCGCGG-- -------CAA CAAGAGC--- CACCCGATCA

Oryzae TGCGCGAGGG CGCGCAGATC GTGCGCGG-- -------CAA CAAGAGC--- CACTCGATCA

Campestris TGCGCGAAGG CGCGCAGATC CTGCGTGG-- -------CAA CAAGAGC--- CACCCGATCA

Xyl9a5C TTCGTAACGG CGCTAAAATT GTTCGTGG-- -------CAC TAAATCC--- CAATCAATTG

XylTemecul TTCGTAACGG CGCTAAAATT GTTCGTGG-- -------CAC TAAATCG--- CAATCAATAG

Burkholder TCGGCGCGGG GGCGGCGATC AAGCGCGG-- -------CGA GCGGAGC--- TCGAACGTGA

Ralstonia TCGGGGAGGG GGCCAAGGTG CGGCGTGGGA CTGGGGATAA GCAGCGCGAG CAGGGTGTGA

....|....| ....|....| ....|....| ....|....| ....|....| ....|....|

2285 2295 2305 2315 2325 2335

X.albiline CCAGCTTTGC CCAGGCGCAT GCCTGGCTGT TCGAAGAAGC CAAAAAAGGC CGTCAGATCC

StenoK279a ACAGCTTTGC CAAGGCACAG AACTGGCTGC TCGAGGAGGC CAAGCGCGGC CGCCAGATCC

StenoR551 ACACGTTCGC CAAGGCGCAG AACTGGCTGC TCGAGGAGGC CAAGCGCGGC CGCCAGATCC

Vesicatori CCAGCTTCGC GCAGGCCCAT GCCTGGTTGC TGGACGAGGC CAAGAAGGGC CGCCAGGTGC

Citri CCAGCTTCGC GCAGGCCCAT GCCTGGTTGC TGGACGAGGC CAAGAAGGGC CGCCAGGTGC

Oryzae CCAGCTTCGC GCAGGCGCAC GCCTGGCTGC TGGACGAGGC CAAGAAGGGT CGCCAGGTCC

Campestris CCAGCTTTGC GCAGGCGCAG GCCTGGCTGC TGGAAGAAGC TAAGCGGGGC CGCCAGGTGC

Xyl9a5C AGTGTTTTGG TGATGCGCAA GCTTGGTTGC TTGAAGATGC GAAGAAAGGT CGCCAAATCC

XylTemecul AGTGTTTTGG TGATGCGCAA GCTTGGTTGC TTGAAGAGGC GAAGAAAGGT CGCCAAATCC

Burkholder CGGACTTCAA GGAAGCGATG AAGTGGCTGA TGGCCGACGC TGAGCGGAAC CTGTCTAAGC

Ralstonia CGGACTTCCA TGCGGCGATT ACTTGGCTGC TGGGTGAGGC TGAGCGTGGC ATCAGCCGGC

....|....| ....|....| ....|....| ....|....| ....|....| ....|....|

2345 2355 2365 2375 2385 2395

X.albiline AGCGCTTCAA GGGTCTGGGC GAAATGAATG CCGAACAGCT GTGGGAAACC ACGGTCAACC

StenoK279a AGCGATTCAA GGGTCTGGGT GAAATGAATC CGGAGCAGCT GTGGGACACC ACGGTGAACC

StenoR551 AGCGATTCAA GGGTCTGGGT GAAATGAATC CGGAGCAGTT GTGGGACACC ACGGTGAACC

Vesicatori AGCGCTTCAA GGGCCTGGGC GAAATGAATG CCGAGCAGCT GTGGGAAACC ACGGTCAACC

Citri AGCGCTTCAA GGGCCTGGGC GAAATGAATG CCGAGCAGCT ATGGGAAACC ACGGTCAACC

Oryzae AGCGCTTCAA GGGCCTGGGC GAAATGAACG CCGAGCAGCT CTGGGAAACC ACCGTCAACC

Campestris AGCGCTTCAA GGGCCTGGGC GAAATGAACG CCGAGCAGTT GTGGGAAACC ACGGTCAATC

Xyl9a5C AGCGCTTTAA GGGTTTGGGT GAAATGAATC CAGAGCAGCT ATGGGATACT ACGGTGAATC

XylTemecul AGCGCTTTAA GGGGTTGGGT GAAATGAATC CGGAGCAGTT ATGGGATACT ACGGTGAATC

Burkholder AGCGGTATAA GGGGTTGGGC GAGATGAACC CCGAGCAGCT GTGGGAAACG ACGATGGACC

Ralstonia AACGTTATAA GGGGCTGGGG GAGATGAACC CCTCGCAGCT GTGGGAGACC ACTATGGATG

....|....| ....|....| ....|....| ....|....| ....|....| ....|....|

2405 2415 2425 2435 2445 2455

X.albiline CCGACACCCG CCGCTTGTTG CAGGTGCGCA TCGAGGACGC CGTGGCAGCC GATCAGATTT

StenoK279a CGGAAACCCG CCGACTGCTG CAGGTGCGTA TCGAGGACGC TGTCGCAGCC GACCAGATCT

StenoR551 CGGAGACCCG CCGACTGCTG CAGGTGCGTA TCGAAGATGC CGTCGCAGCT GACCAGATCT

Vesicatori CCGACACGCG TCGCCTGTTG CAGGTGCGCA TCGAGGACGC GGTGGCCGCC GACCAGATCT

Citri CCGACACGCG TCGCCTGTTG CAGGTGCGCA TCGAGGACGC GGTGGCCGCC GACCAGATCT

Oryzae CCGACACCCG TCGCCTGTTG CAGGTGCGGA TCGAGGATGC GGTGGCCGCC GACCAGATCT

Campestris CGGACACGCG GCGCCTGCTG CAGGTGCGCA TTGAAGACGC CGTGGCGGCC GACCAGATCT

Xyl9a5C CGGACACTCG CCGTCTTTTG CAAGTTTGTA TTGAAGATGG GATTGCTGCT GACCAAATCT

XylTemecul CGGACACTCG CCGTCTTTTG CAAGTTTGTA TTGAAGATGG GATTGCTGCT GACCAAATCT

Burkholder CGGCGGTGCG GCGGTTGCTG CGCGTGCAGA TCGAGGATGC GATTGCGGCC GATGGGATCT

Ralstonia TTACGCAGCG GCGACTGCTC AAGGTGCAGA TTGAAGACGC TATTGCGGCG GATCAGATCT

....|....| ....|....| ....|....| ....|....| ....|....| ....|....|

2465 2475 2485 2495 2505 2515

X.albiline TCAGCACCTT GATGGGCGAT GTGGTCGAGC CGCGTCGTGA TTTCATCGAG GAAAACGCGC

StenoK279a TCAGCACCCT GATGGGCGAT GTCGTCGAAC CGCGTCGCGA TTTCATCGAA GACAACGCGC

StenoR551 TCAGCACCTT GATGGGTGAT GTCGTCGAAC CGCGTCGCGA TTTCATCGAA GACAACGCGC

Vesicatori TCAGCACCTT GATGGGCGAT GTGGTCGAGC CACGCCGCGA CTTCATCGAG GACAACGCGC

Citri TCAGCACCTT GATGGGCGAT GTGGTCGAAC CACGCCGCGA CTTCATCGAG GACAACGCGC

Oryzae TCAGCACCCT GATGGGCGAT GTGGTCGAAC CACGCCGCGA CTTCATCGAG GACAACGCGC

Campestris TCAGCACCTT GATGGGCGAT GTGGTGGAAC CGCGCCGCGA CTTCATCGAG GACAACGCGC

Xyl9a5C TCAGCACACT GATGGGTGAT GTGGTAGAGC CACGCCGTGG CTTTATTGAG AACAATGCAT

XylTemecul TCAGCACACT GATGGGTGAT GTGGTAGAGC CACGCCGTGG CTTTATTGAG AACAATGCAT

Burkholder TTACCACCCT CATGGGGGAT GAGGTCGAGC CGCGTAGGGC GTTTATCGAG AATAATGCGC

Ralstonia TTACTACGCT GATGGGGGAT GACGTGGAGC CTCGGCGGAA CTTTATTGAG AGTAATGCGT

....|....| ....|....| ....|....| ....|....| ....|....| ....|....|

2525 2535 2545 2555 2565 2575

X.albiline TAAAGGTCGC CAACCTCGAC ATTTGAATGA GTCAGG---- ---------- -GCAAGATCG

StenoK279a TGAAGGTCGC AAACCTGGAT ATCTGAATGA GTCAGG---- ---------- -GCAAGATCG

StenoR551 TGAAGGTCGC AAACCTGGAT ATCTGAATGA GTCAGG---- ---------- -GCAAGATCG

Vesicatori TGAAGGTGTC CAACCTGGAT ATCTGAATGA GTCAGG---- ---------- -GCAAGATCG

Citri TGAAGGTGTC CAACCTGGAT ATCTGAATGA GTCAGG---- ---------- -GCAAGATCG

Oryzae TGAAGGTGTC CAACCTGGAT ATTTGAATGA GTCAGG---- ---------- -GCAAGATCG

Campestris TGAAGGTTTC CAACCTGGAT ATCTGAATGA GTCAGG---- ---------- -GCAAGATCG

Xyl9a5C TAAAAGTGAC CCGCTTGGAT ATTTAAATGA ATCAGG---- ---------- -GTAAGATCG

XylTemecul TAAAAGTGAC TCGCTTGGAT ATTTAAATGA ATCAGG---- ---------- -GTAAGATCG

Burkholder TAAGGGCGGG GAATATTGAT GTTTGAATGA GTACTGCTGC TTTGGTAGAA GGCAAGATCG

Ralstonia TGGTGGCGCG GAATATTGAT GTTTGAATGA GTATCG---- ---------- -GAACGATTG

....|....| ....|....| ....|....| ....|....| ....|....| ....|....|

2585 2595 2605 2615 2625 2635

X.albiline TTCAGATCAT CGGCGCGGTC GTCGACGTCG AATTCGCGCG GGCCGATGTG CCGAAGATTT

StenoK279a TTCAGATCAT CGGCGCGGTC GTCGACGTCG AATTCCCGCG TGAATCGGTG CCGAAGGTGT

StenoR551 TTCAGATCAT CGGCGCCGTC GTCGACGTCG AATTCCCGCG TGAGTCGGTG CCGAAGGTGT

Vesicatori TTCAGATCAT CGGCGCGGTC GTCGACGTCG AGTTCCAGCG CAATGAAGTG CCGAAGGTGT

Citri TTCAGATCAT CGGCGCGGTC GTCGACGTCG AATTCCCGCG CAATGAAGTG CCGAAGGTGT

Oryzae TTCAGATCAT CGGCGCGGTC GTCGACGTCG AATTCCCGCG CAATGAAGTG CCGAAGGTGT

Campestris TTCAGATCAT CGGCGCGGTC GTCGACGTCG AATTCCAGCG CAATGAAGTG CCGAAGGTCT

Xyl9a5C TTCAGATCAT CGGCGCAATT GTCGACGTCG AATTCCCACG AAACAATGTC CCAAAAGTAT

XylTemecul TTCAGATCAT CGGCGCAATT GTCGACGTCG AATTCCCACG AAACAATGTC CCCAAAGTAT

Burkholder TACAGTGCAT CGGCGCCGTT ATCGACGTGG AATTCCCGCG CGAGAGCATG CCGAAGATCT

Ralstonia TGCAGTGCAT CGGCGCCGTG GTGGACATCC AGTTCCCGCG CGACGCGATG CCCAAGGTCT

....|....| ....|....| ....|....| ....|....| ....|....| ....|....|

2645 2655 2665 2675 2685 2695

X.albiline ACGACGCATT GAAGGTCGAA GGCA------ ---------- --CCGCCA-- ----TCACGC

StenoK279a ACGACGCACT GAAGGTGGAA AACA------ ---------- --CCGAGA-- ----TCACCC

StenoR551 ATGACGCACT GAAGGTGGAA AACA------ ---------- --CCGAGA-- ----TCACCC

Vesicatori ACGACGCGCT GAAGGTCGAA GGCA------ ---------- --CCGCCA-- ----TCACCC

Citri ATCACGCGCT GAAGGTCGAC GGCA------ ---------- --CCGAAA-- ----TCACCC

Oryzae ATCACGCGCT GAAGGTCGAA GGCA------ ---------- --CCGAAA-- ----TCACCC

Campestris ATCACGCGCT GAAGGTCGAA GGCA------ ---------- --CCGCCA-- ----TCACCC

Xyl9a5C ATAACGCGCT GAAGATCGAC GGCA------ ---------- --CAGCCA-- ----TCATCT

XylTemecul ATAACGCGCT GAAGATCGAC GGCA------ ---------- --CAGCCA-- ----TCATCT

Burkholder ACGACGCGCT CATTCTGGAA GGC------- ---------- --TCGGAA-- ---CTGACGC

Ralstonia ACGACGCGCT CGTGCTGCAG GACAGCGGCG AAGCGTCGTT CGCCGAGAAG GGCCTGAGCT

....|....| ....|....| ....|....| ....|....| ....|....| ....|....|

2705 2715 2725 2735 2745 2755

X.albiline TGGAAGTACA GCAGCAGCTG GGCGATGGCG TTGTGCGCAC CATTGCCCTC GGCTCCACCG

StenoK279a TCGAAGTCCA GCAGCAGCTG GGCGACGGCG TGGTGCGTAC CATCGCCCTC GGTTCCACCG

StenoR551 TTGAAGTCCA GCAGCAGCTG GGCGACGGCG TGGTCCGTTG CATCGCGCTG GGTTCCACCG

Vesicatori TGGAAGTGCA GCAGCAGCTG GGCGACGGCG TCGTGCGCAC CATTGCGCTC GGTTCCACCG

Citri TGGAAGTGCA GCAGCAGCTC GGCGACGGCG TCGTGCGCAC GATTGCGCTC GGCTCCACCG

Oryzae TGGAAGTGCA GCAGCAGCTC GGCGACGGCG TCGTGCGCAC GATTGCGCTC GGCTCCACCG

Campestris TGGAAGTGCA GCAGCAGCTC GGCGACGGCG TCGTGCGCAC GATTGCGCTC GGCTCCACCG

Xyl9a5C TGGAAGTGCA GCAACAGCTT GGCGACGGCA TTGTACGCAC TATAGCACTT GGCTCCACAG

XylTemecul TGGAAGTGCA GCAACAGCTT GGCGACGGCA TTGTACGCAC AATAGCACTT GGCTCCACAG

Burkholder TCGAAGTCCA GCAGCAGCTG GGCGACGGCG TGGTCCGTAC CATCTGTCTG GGTGCCTCCG

Ralstonia TCGAAGTGCA ACAACAGCTG GGCGACGGCG TGGTGCGTAC CATTGCGCTG GGTTCGTCGG

....|....| ....|....| ....|....| ....|....| ....|....| ....|....|

2765 2775 2785 2795 2805 2815

X.albiline ATGGCCTCAA GCGCAACCTG GTCGCGACCA ACACTGGCCG CGCGATCGCG GTGCCGGTCG

StenoK279a ACGGCCTGAA GCGCAACCTG GTTGCCGTCA ACACCGGCCG TGGCATCTCG GTGCCGGTCG

StenoR551 ATGGCCTGAA GCGCAACCTG GTTGCGGTCA ACACCGGCCG TGGCATCTCG GTGCCGGTCG

Vesicatori ACGGCCTCAA GCGCAACCTG GTCGCCACCA ACACCGAACG CGCCATCTCG GTGCCGGTCG

Citri ACGGCCTGAA GCGCAACCTG GTCGCCACCA ACACCGAACG CGCCATCTCG GTGCCGGTCG

Oryzae ACGGCTTGAA GCGCAACCTG CTGGCCACCA ACACCGAGCG CGCCATCTCG GTGCCGGTCG

Campestris ACGGATTGAA GCGCAACCTG CTGGCCACCA ACACCGAGCG TGCGATTTCG GTGCCGGTTG

Xyl9a5C ACGGTTTAAA ACGCAACCTC ATTGCCACGG ACACCGGCCA TGCAATCACA GTTCCCGTAG

XylTemecul ACGGCTTAAA ACGCAACCTC ATTGCCACCG ACACCGGCCA TGCAATCACA GTTCCCGTAG

Burkholder ACGGCCTGCG CCGCGGCGTC GTGGTGAAGA ATACGGGCAA TCCGATTTCG GTGCCGGTCG

Ralstonia ACGGCCTGCG CCGCGGCATG CCGGTGTCGA ACACTGGCGC GCCGATCTCG GTGCCCGTCG

....|....| ....|....| ....|....| ....|....| ....|....| ....|....|

2825 2835 2845 2855 2865 2875

X.albiline GCGCGGGCAC GCTGGGCCGC ATCATGGACG TGCTGGGCCG TCCGATCGAC GAAGCCGGTG

StenoK279a GCGCCGGCAC CCTGGGCCGC ATCATGGACG TGCTCGGCCG TCCGATCGAC GAAGCCGGCC

StenoR551 GCGCCGGTAC CCTGGGCCGC ATCATGGACG TGCTTGGCCG TCCGATCGAC GAAGCCGGCC

Vesicatori GCGCCGGTAC CCTGGGCCGC ATCATGGACG TGCTGGGTCG CCCGATCGAC GAGGCCGGCG

Citri GCGCCGGTAC GCTGGGCCGC ATCATGGACG TGCTGGGTCG CCCGATCGAC GAAGCTGGCG

Oryzae GTGCCGGTAC GCTGGGCCGC ATCATGGACG TGCTGGGTCG TCCGATCGAC GAAGCCGGCG

Campestris GCGCCGGGAC GCTGGGCCGC ATCATGGATG TGCTGGGTCG CCCGATCGAC GAAGCCGGCG

Xyl9a5C GCACTGGCAC ATTAGGCCGC ATCATGGATG TACTAGGCAA CCCAATCGAC GAAGCAGGTC

XylTemecul GCACTGGCAC ATTAGGTCGC ATCATGGATG TACTAGGCAA CCCAATCGAC GAAGCAGGTC

Burkholder GCAAGCCGAC CCTCGGCCGC ATCATGGACG TGCTCGGCCG CCCGATCGAC GAGGCGGGAC

Ralstonia GCCACGGCAC GCTGGGCCGC ATCATGGACG TGCTGGGTCG CCCGATCGAC GAAGCCGGTC

....|....| ....|....| ....|....| ....|....| ....|....| ....|....|

2885 2895 2905 2915 2925 2935

X.albiline AGGTGCAGGC CACCGACCAT TGGGAAATCC ACCGCGCTGC GCCGACATAT GAAGATCAGT

StenoK279a CGGTGGCCGC CAGCGACAGC TGGGAAATCC ACCGTGCGGC CCCGTCGTAC GAAGACCAGT

StenoR551 CGGTGGCCGC CAGCGACAGC TGGGAAATCC ACCGCGACGC GCCGTCGTAC GAAGACCAGT

Vesicatori ACGTGCAGGC GTCCGATCAT TGGGAAATCC ACCGTAGCGC CCCGTCCTAC GAGGACCAGG

Citri ACGTGCAGGC CTCGGACCAT TGGGAAATCC ATCGCGGCGC ACCGTCGTAT GAAGACCAGT

Oryzae ACGTGCAGGC GTCGGACCAT TGGGAAATCC ATCGCGGCGC ACCGTCGTAC GAAGACCAGT

Campestris ACGTGCAGGC CTCGGATCAT TGGGAAATCC ACCGCGCTGC GCCGTCGTAC GAAGACCAGT

Xyl9a5C CGATCACTTA TACAGATCAA TGGGAAATCC ATCGAAACGC TCCTTCCTAT GAAGATCAAG

XylTemecul CGATCACTTA TACAGATCAA TGGGAAATCC ATCGAAACGC TCCTTCCTAT GAAGATCAAG

Burkholder CGATCGAGAG CGAAAACAAG CGCTCGATCC ACCAGAAGGC GCCGGCGTTC GACGAGCTGT

Ralstonia CGATCGCCGC CGATGAAAAG CGCGCGATTC ACCAGAAGGC CCCGAAGTTC GACGAACTGT

....|....| ....|....| ....|....| ....|....| ....|....| ....|....|

2945 2955 2965 2975 2985 2995

X.albiline CCTCGGCCAC CGAATTGCTG GAAACCGGCA TCAAGGTCAT CGACCTGATG TGCCCGTTCG

StenoK279a CCCCGGCCAC CGAACTGCTG GAAACCGGCA TCAAGGTCAT CGACCTGATG TGCCCGTTCG

StenoR551 CCCCGGCCAC CGAGCTGCTG GAAACCGGCA TCAAGGTCAT CGACCTGATG TGCCCGTTCG

Vesicatori CGTCGACCAC CGAACTGCTG GAAACCGGCA TCAAGGTGAT CGACCTGATG TGCCCGTTCG

Citri CGTCCAGCAC CGAATTGCTG GAAACCGGCA TCAAGGTCAT CGACCTGATG TGCCCGTTCG

Oryzae CCTCCAGCAC CGAATTGCTG GAAACCGGCA TCAAGGTCAT CGACCTGATG TGCCCGTTCG

Campestris CGTCCAGCAC CGAATTGCTG GAAACCGGCA TCAAGGTGAT CGACCTGATG TGCCCGTTCG

Xyl9a5C CCTCAACCAC TGAACTGCTG GAGACCGGAA TCAAGGTCAT CGATCTTATG TGTCCATTTG

XylTemecul CCTCAACCAC CGAACTGCTG GAGACCGGAA TCAAGGTCAT CGACCTTATG TGTCCATTTG

Burkholder CGCCGTCGAC CGAACTGCTC GAAACGGGCA TCAAGGTCAT CGATCTGATC TGCCCGTTCG

Ralstonia CGCCGTCGGT GGACCTGCTG GAAACCGGCA TCAAGGTGAT CGACCTGGTT TGCCCGTTCG

....|....| ....|....| ....|....| ....|....| ....|....| ....|....|

3005 3015 3025 3035 3045 3055

X.albiline CCAAGGGCGG CAAGGTCGGC CTGTTCGGCG GCGCCGGTGT CGGCAAGACC GTCAACATGA

StenoK279a CCAAGGGCGG CAAGGTCGGC CTGTTCGGCG GCGCCGGCGT CGGCAAGACC GTCAACATGA

StenoR551 CCAAGGGCGG CAAGGTCGGT CTGTTCGGCG GCGCCGGCGT CGGCAAGACC GTCAACATGA

Vesicatori CCAAGGGCGG CAAGGTCGGC CTGTTCGGCG GCGCCGGCGT CGGCAAGACC GTCAACATGA

Citri CCAAGGGCGG CAAGGTCGGC CTGTTCGGCG GTGCCGGCGT CGGCAAGACC GTCAACATGA

Oryzae CCAAGGGCGG CAAGGTCGGC CTGTTCGGCG GCGCCGGCGT CGGCAAGACC GTCAACATGA

Campestris CCAAGGGCGG CAAGGTCGGC CTGTTCGGCG GCGCCGGCGT CGGCAAGACC GTCAACATGA

Xyl9a5C CTAAAGGCGG CAAAGTCGGA TTATTCGGTG GTGCCGGTGT CGGCAAAACT GTCAACATGA

XylTemecul CTAAAGGCGG CAAAGTCGGA TTATTCGGTG GTGCCGGTGT CGGCAAAACT GTCAACATGA

Burkholder CAAAGGGCGG CAAGGTCGGC CTGTTCGGCG GCGCGGGCGT GGGCAAGACC GTCAACATGA

Ralstonia CCAAGGGCGG CAAGGTGGGT CTGTTCGGTG GCGCCGGCGT CGGCAAGACC GTGAACATGA

....|....| ....|....| ....|....| ....|....| ....|....| ....|....|

3065 3075 3085 3095 3105 3115

X.albiline TGGAGCTGAT CAACAACATC GCCAAGGCAC ACGCAGGCTT GTCGGTGTTT GCTGGCGTGG

StenoK279a TGGAGCTGAT CAACAACATC GCCAAGGCGC ACAGCGGTCT GTCCGTGTTC GCCGGCGTGG

StenoR551 TGGAACTGAT CAACAACATC GCCAAGGCGC ACAGCGGTCT GTCCGTGTTC GCCGGCGTGG

Vesicatori TGGAACTGAT CAACAACATC GCCAAGGCGC ACTCGGGTCT GTCGGTGTTC GCCGGCGTGG

Citri TGGAACTGAT CAACAACATC GCCAAGGCGC ACAGCGGCTT GTCCGTGTTC GCCGGCGTGG

Oryzae TGGAACTGAT CAACAACATC GCCAAGGCGC ACAGCGGCTT GTCCGTGTTC GCCGGCGTGG

Campestris TGGAACTGAT CAACAACATC GCCAAGGCGC ACTCGGGCCT GTCCGTGTTC GCCGGCGTGG

Xyl9a5C TGGAATTGAT CAATAACATC GCCAAGGCAC ATAGTGGGCT GTCCGTATTC GCGGGTGTTG

XylTemecul TGGAATTGAT CAATAACATC GCCAAGGCAC ATAGTGGGCT GTCCGTATTC GCGGGTGTTG

Burkholder TGGAGCTCAT CAACAACATC GCGAAGGAGC ACGGCGGTTA CTCCGTGTTC GCGGGCGTGG

Ralstonia TGGAGCTGAT CAACAACATC GCCAAGCAGC ACTCGGGCTT GTCGGTGTTT GCTGGCGTGG

....|....| ....|....| ....|....| ....|....| ....|....| ....|....|

3125 3135 3145 3155 3165 3175

X.albiline GCGAGCGGAC CCGCGAGGGC AACGATTTCT ACCACGAAAT GAAGGACTCC AACGTCCTGG

StenoK279a GTGAGCGTAC CCGTGAGGGC AACGACTTCT ACCACGAAAT GAAGGACTCC AACGTCCTCG

StenoR551 GTGAGCGTAC CCGTGAGGGC AACGACTTCT ACCACGAGAT GAAGGACTCC AACGTCCTGG

Vesicatori GCGAGCGTAC CCGCGAGGGC AACGACTTCT ACCACGAGAT GAAGGACTCC AACGTCCTGG

Citri GCGAGCGTAC CCGCGAGGGC AACGACTTCT ACCACGAGAT GAAGGACTCC AACGTCCTGG

Oryzae GCGAGCGTAC CCGCGAGGGC AACGACTTCT ACCACGAGAT GAAGGACTCC AACGTTCTGG

Campestris GTGAGCGTAC CCGTGAGGGC AACGACTTCT ACCACGAGAT GAAGGACTCC AACGTCCTGG

Xyl9a5C GCGAGCGAAC CCGCGAAGGT AACGACTTCT ATCACGAGAT GAAAGACTCC AACGTTCTCG

XylTemecul GCGAGCGAAC CCGCGAAGGT AACGACTTCT ATCACGAGAT GAAAGACTCC AACGTTCTCG

Burkholder GCGAGCGTAC CCGTGAAGGG AACGACTTCT ACCACGAAAT GAAGGACTCG AACGTTCTCG

Ralstonia GCGAGCGTAC CCGTGAGGGC AACGACTTCT ACCACGAAAT GAAGGACTCC AACGTGCTCG

....|....| ....|....| ....|....| ....|....| ....|....| ....|....|

3185 3195 3205 3215 3225 3235

X.albiline ACAAGGTCGC GATGGTGTAT GGCCAGATGA ACGAGCCGCC GGGCAACCGA TTGCGCGTCG

StenoK279a ACAAGGTGGC GATGGTGTAC GGCCAGATGA ACGAGCCGCC GGGCAACCGT CTGCGCGTCG

StenoR551 ACAAGGTCGC GATGGTGTAC GGCCAGATGA ACGAGCCGCC GGGCAACCGT CTGCGCGTTG

Vesicatori ACAAGGTGGC GATGGTGTAC GGCCAGATGA ACGAGCCGCC GGGCAACCGT CTGCGCGTGG

Citri ACAAGGTGGC GATGGTGTAC GGCCAGATGA ACGAGCCGCC GGGCAACCGT CTGCGCGTGG

Oryzae ACAAGGTCGC GATGGTGTAC GGCCAGATGA ACGAGCCGCC GGGCAACCGT CTGCGCGTTG

Campestris ACAAGGTCGC GATGGTGTAC GGCCAGATGA ACGAGCCGCC GGGCAACCGT CTGCGCGTTG

Xyl9a5C ACAAGGTAGC AATGGTGTAT GGCCAGATGA ACGAACCGCC GGGTAACCGT CTGCGCGTTG

XylTemecul ACAAGGTAGC AATGGTGTAT GGGCAGATGA ACGAACCACC GGGTAACCGT CTGCGCGTTG

Burkholder ACAAGGTCGC GCTGGTGTAC GGCCAGATGA ACGAGCCGCC GGGCAACCGT CTGCGCGTGG

Ralstonia ACAAGGTGGC CATGGTGTTC GGCCAGATGA ACGAGCCGCC GGGCAACCGT CTGCGCGTGG

....|....| ....|....| ....|....| ....|....| ....|....| ....|....|

3245 3255 3265 3275 3285 3295

X.albiline CACTGACCGG CCTGACCATG GCCGAGTACT TCCGCGACGA AAAGGATGCT TCGGGCAAGG

StenoK279a CCCTGACCGG CCTGACCATG GCCGAGTACT TCCGCGACGA GAAGGACGAA AACGGCAAGG

StenoR551 CGCTGACCGG CCTGACCATG GCCGAGTACT TCCGCGACGA GAAGGACGAG AACGGCAAGG

Vesicatori CGTTGACCGG CCTGACCATG GCCGAGTACT TCCGCGACGA GAAGGATGCC AGCGGCAAGG

Citri CGTTGACCGG CCTGACCATG GCCGAGTACT TCCGCGACGA GAAGGACGCC AGCGGTAAGG

Oryzae CGCTGACCGG CCTGACCATG GCCGAGTACT TCCGCGACGA GAAGGACGCC AACGGCAAGG

Campestris CGCTGACCGG CCTGACCATG GCCGAGTACT TCCGCGACGA GAAGGACGAG AACGGCAAGG

Xyl9a5C CGCTCACCGG TCTCACCATG GCCGAGTACT TCCGCGATGA GAAAGACAGT AGCGGCAAAG

XylTemecul CGCTCACCGG TCTCACCATG GCCGAGTACT TCCGCGATGA GAAAGACAGT AGCGGCAAAG

Burkholder CGCTGACGGG CCTCACGATG GCCGAGCACT TCCGTGACGA ---------- --------AG

Ralstonia CGCTGACCGG CCTGACCATG GCCGAGCGCT TCCGTGACGA ---------- --------AG

....|....| ....|....| ....|....| ....|....| ....|....| ....|....|

3305 3315 3325 3335 3345 3355

X.albiline GCAAGGACGT GCTGCTGTTC GTCGATAACA TCTACCGCTA CACCCTGGCC GGTACCGAAG

StenoK279a GCAAGGACGT CCTGCTGTTC GTCGACAACA TCTACCGCTA CACCCTGGCC GGTACCGAAG

StenoR551 GCAAGGACGT GCTGCTGTTC GTCGACAACA TCTACCGCTA CACCCTGGCC GGTACCGAAG

Vesicatori GCAAGGACGT GCTGCTGTTC GTGGACAACA TCTACCGCTA CACGCTGGCC GGTACCGAAG

Citri GCAAGGACGT GCTGCTGTTC GTCGACAACA TCTACCGCTA CACGCTGGCC GGTACCGAAG

Oryzae GCAAGGACGT GCTGCTGTTC GTGGACAACA TTTACCGCTA CACGCTGGCC GGTACCGAAG

Campestris GCAAGGACGT GCTGCTGTTC GTGGACAACA TCTACCGCTA CACGCTGGCC GGTACCGAAG

Xyl9a5C GCAAAGATGT ACTTCTATTC ATCGACAATA TCTACCGCTA CACCTTAGCT GGCACGGAAG

XylTemecul GCAAAGATGT ACTTCTATTC ATCGACAATA TCTACCGCTA CACCTTAGCT GGCACGGAAG

Burkholder GCCTCGACGT GCTGTTCTTC GTCGACAACA TCTACCGTTT CACGCTGGCC GGTACCGAAG

Ralstonia GCCGCGACAT CCTGTTCTTC GTCGACAACA TCTACCGCTA CACGCTGGCC GGTACCGAAG

....|....| ....|....| ....|....| ....|....| ....|....| ....|....|

3365 3375 3385 3395 3405 3415

X.albiline TGTCGGCGTT GCTGGGCCGC ATGCCGTCGG CGGTGGGTTA CCAGCCGACC CTGGCCGAGG

StenoK279a TGTCGGCACT GCTGGGCCGC ATGCCGTCCG CCGTCGGTTA CCAGCCGACC CTGGCCGAGG

StenoR551 TGTCGGCACT GCTGGGCCGC ATGCCGTCGG CAGTGGGTTA CCAGCCGACC CTGGCCGAGG

Vesicatori TGTCGGCGCT GCTCGGCCGC ATGCCGTCGG CGGTGGGCTA CCAGCCGACC CTGGCCGAGG

Citri TGTCCGCGCT GCTCGGCCGT ATGCCGTCGG CCGTGGGCTA CCAGCCGACC CTGGCCGAAG

Oryzae TGTCCGCGCT GCTCGGCCGT ATGCCGTCGG CCGTGGGTTA CCAGCCCACT CTGGCCGAAG

Campestris TGTCCGCGCT GCTTGGCCGC ATGCCGTCGG CGGTGGGTTA CCAGCCGACC CTGGCCGAGG

Xyl9a5C TATCCGCCCT ACTCGGTCGC ATGCCCTCAG CAGTAGGCTA CCAACCAACC CTTGCCGAGG

XylTemecul TATCCGCCCT ACTCGGTCGC ATGCCCTCAG CAGTAGGCTA CCAACCAACC CTTGCCGAGG

Burkholder TGTCGGCGCT GCTCGGCCGT ATGCCGTCGG CAGTGGGCTA TCAGCCGACG CTGGCTGAAG

Ralstonia TGTCGGCACT GCTGGGCCGG ATGCCTTCCG CCGTGGGCTA TCAGCCGACG CTGGCTGAAG

....|....| ....|....| ....|....| ....|....| ....|....| ....|....|

3425 3435 3445 3455 3465 3475

X.albiline AAATGGGCGT GTTGCAGGAG CGCATCACCT CCACCAAGAA CGGCTCGATC ACCTCGATCC

StenoK279a AAATGGGCGT CCTGCAGGAG CGCATCACTT CGACCAAGAA CGGTTCGATC ACCTCGATCC

StenoR551 AAATGGGCGT CCTGCAGGAG CGCATCACCT CGACCAAGAA CGGTTCGATC ACCTCGATCC

Vesicatori AAATGGGCGT GCTGCAGGAG CGCATCACCT CGACCAAGAG CGGTTCGATC ACCTCGATCC

Citri AGATGGGCGT GCTGCAGGAG CGCATCACCT CGACCAAGAG CGGTTCGATC ACCTCGATCC

Oryzae AAATGGGCGT GCTGCAGGAA CGCATCACCT CGACCAAGAG CGGTTCGATC ACCTCGATCC

Campestris AAATGGGCGT GTTGCAGGAG CGCATCACCT CGACCAAGAG CGGTTCGATC ACCTCGATCC

Xyl9a5C AAATGGGCGT ACTACAAGAG CGTATCACCT CAACCGCAAA TGGCTCGATC ACCTCGATCC

XylTemecul AAATGGGCGT ACTACAAGAA CGTATCACCT CAACCGCAAA TGGCTCGATC ACCTCGATCC

Burkholder AAATGGGCAA GCTGCAAGAG CGCATCACGT CGACGAAGAA GGGCTCGATC ACGTCGGTTC

Ralstonia AAATGGGCAA GCTGCAGGAG CGCATCACGT CGACCAAGAC CGGTTCGATC ACGTCGATCC

....|....| ....|....| ....|....| ....|....| ....|....| ....|....|

3485 3495 3505 3515 3525 3535

X.albiline AGGCGGTGTA CGTGCCCGCG GACGACCTCA CCGACCCGTC GCCGGCGACC ACCTTCGCCC

StenoK279a AGGCCGTCTA CGTTCCCGCG GACGACCTGA CCGACCCGTC GCCGGCAACC ACCTTCGCCC

StenoR551 AGGCCGTCTA CGTTCCCGCG GATGACTTGA CCGATCCGTC GCCGGCGACC ACCTTCGCCC

Vesicatori AGGCCGTGTA CGTGCCTGCG GACGACCTGA CCGACCCGTC GCCGGCGACC ACCTTCGCCC

Citri AGGCCGTGTA CGTGCCCGCG GACGACCTGA CCGACCCGTC GCCGGCGACC ACCTTCGCCC

Oryzae AGGCCGTGTA CGTGCCTGCG GACGACCTGA CCGACCCGTC GCCGGCGACC ACCTTCGCCC

Campestris AGGCCGTGTA CGTGCCCGCG GACGACCTGA CCGACCCGTC GCCGGCGACC ACCTTCGCCC

Xyl9a5C AAGCAGTGTA CGTACCCGCC GATGATCTGA CTGACCCATC ACCTGCGACG ACCTTTGGAC

XylTemecul AAGCAGTGTA CGTACCCGCC GATGATCTGA CTGACCCATC ACCTGCGACG ACCTTTGGAC

Burkholder AGGCCGTGTA CGTGCCTGCG GACGACTTGA CCGACCCGTC GCCGGCCACC ACCTTCGGCC

Ralstonia AGGCCGTGTA CGTGCCTGCG GATGACTTGA CCGATCCGTC GCCCGCTACG ACCTTCCTGC

....|....| ....|....| ....|....| ....|....| ....|....| ....|....|

3545 3555 3565 3575 3585 3595

X.albiline ACCTCGACGC CACCGTCGTG CTGTCGCGTA ACATCGCCTC GCTGGGCATC TATCCGGCGG

StenoK279a ACCTGGACTC GACCGTTACC CTGTCGCGTT CGATCGCCTC GCTGGGTATC TACCCGGCCG

StenoR551 ACCTGGATTC GACCGTGACC CTGTCGCGTT CGATCGCCTC GCTGGGTATC TACCCGGCAG

Vesicatori ACCTGGATTC CACCGTCACG CTGAGCCGTA ACATCGCCTC GCTGGGTATC TACCCGGCGG

Citri ACTTGGACTC GACCGTTACG CTGAGCCGTA ACATCGCCTC GCTGGGTATC TACCCGGCGG

Oryzae ACTTGGACTC GACCGTCACG CTGAGCCGTA ACATCGCTTC GCTGGGTATC TACCCGGCTG

Campestris ACTTGGACTC GACGGTGACG CTGAGCCGTA ACATCGCCTC GCTGGGTATC TACCCGGCCG

Xyl9a5C ATCTGGACTC CACAGTGACA CTATCCCGCT CGATCGCCGC ATTAGGCATT TACCCAGCAG

XylTemecul ATCTGGACTC CACAGTGACA CTATCCCGCT CGATCGCCGC ATTAGGCATT TACCCGGCAG

Burkholder ACTTGGACGC AACCGTCGTT CTGTCGCGTG ACATCGCATC GCTCGGTATC TATCCGGCCG

Ralstonia ACCTGGACTC GACCGTCGTG CTGTCGCGTG ACATCGCTGC GCTGGGTATC TACCCCGCCG

....|....| ....|....| ....|....| ....|....| ....|....| ....|....|

3605 3615 3625 3635 3645 3655

X.albiline TGGACCCGTT GGATTCGACC AGCCGCCAGC TCGATCCGAA CGTGATCGGC CACGAGCATT

StenoK279a TCGATCCGCT GGACTCCACC AGCCGCCAGA TGGACCCGCT GGTCATCGGC CACGAGCACT

StenoR551 TCGACCCGCT GGATTCCACC AGCCGCCAGA TGGACCCGCT GGTCATCGGC CACGAGCACT

Vesicatori TGGATCCGCT GGACTCGACC AGCCGCCAGA TGGATCCGCT GGTGATCGGC CACGAGCATT

Citri TGGATCCGCT GGACTCCACC AGCCGCCAGA TGGACCCGCT GGTGATCGGC CACGAGCATT

Oryzae TGGATCCGCT GGACTCCACC AGCCGCCAGA TGGACCCGCT GGTGATCGGC CACGAGCATT

Campestris TGGATCCGCT GGACTCCACG AGCCGCCAGA TGGACCCGCT GGTGATCGGC CACGAGCATT

Xyl9a5C TGGATCCACT GGATTCGAGC AGTCGCCAGA TGGATCCGCT GATCATCGGC GAAGAACACT

XylTemecul TGGATCCACT GGATTCGAGC AGTCGCCAGA TGGATCCGCT GATCATCGGC GAAGAACACT

Burkholder TCGACCCGCT CGACTCGACG TCGCGCCAGA TCGACCCGAA CGTGATCGGC GAAGAGCACT

Ralstonia TCGATCCGCT CGACTCGACC TCGCGTCAGC TGGATCCGCA AATCGTCGGT ACGGAACACT

....|....| ....|....| ....|....| ....|....| ....|....| ....|....|

3665 3675 3685 3695 3705 3715

X.albiline ACGACACCGC GCGCCGCGTG CAGTCCACCT TGCAGAAGTA CAAGGAGCTG AAGGACATCA

StenoK279a ACGACACCGC CCAGCGCGTT CAGCAGACCC TGCAGAAGTA CAAGGAACTG AAGGACATCA

StenoR551 ACGACACCGC CCAGCGCGTC CAGCAGACCC TGCAGAAGTA CAAGGAACTG AAGGACATCA

Vesicatori ACGACACCGC CCAGCGCGTC CAGCAGACCT TGCAGAAGTA CAAGGAACTG AAGGACATCA

Citri ACGACACCGC CCAGCGCGTG CAGCAGACCT TGCAGAAATA CAAGGAACTG AAGGACATCA

Oryzae ACGACACCGC CCAGCGCGTC CAGCAGACCT TGCAGAAGTA CAAGGAACTG AAGGACATCA

Campestris ACGACACCGC CCAGCGCGTC CAGCAGACCT TGCAGAAGTA CAAGGAACTG AAGGACATCA

Xyl9a5C ACAATACCAC CCAACGTGTC CAACAAACTC TGCAAAAATA TAAAGATCTG AAGGACATCA

XylTemecul ACAATACCAC CCAACGTGTC CAACAAACTC TGCAAAAATA TAAAGATCTG AAGGACATCA

Burkholder ACTCGATCAC CCGCCGCGTT CAGCAGACGC TGCAGCGCTA CAAGGAACTG CGCGACATCA

Ralstonia ACGAAGTGGC CCGCCGCGTG CAGCAGACCC TGCAGCGCTA CAAGGAACTG CGCGACATCA

....|....| ....|....| ....|....| ....|....| ....|....| ....|....|

3725 3735 3745 3755 3765 3775

X.albiline TCGCGATCCT GGGCATGGAC GAACTGTCCG AAGAGGACAA GCAGGCCGTG TCGCGCGCGC

StenoK279a TCGCGATCCT GGGCATGGAC GAACTGTCCG AAGAGGACAA GCAGGCCGTG TCGCGCGCCC

StenoR551 TCGCCATCCT GGGCATGGAC GAACTGTCCG AAGAAGACAA GCAGGCCGTG TCGCGCGCAC

Vesicatori TCGCCATCCT GGGCATGGAC GAGCTGAGCG AAGAAGACAA GCAGTCGGTG TCGCGCGCGC

Citri TCGCCATCCT GGGCATGGAC GAGCTGAGCG AAGAAGACAA GCAGTCGGTG TCGCGCGCAC

Oryzae TCGCCATCCT GGGCATGGAC GAGCTGAGCG AAGAAGACAA GCAGTCGGTG TCGCGCGCAC

Campestris TCGCGATCCT GGGCATGGAC GAGCTGAGCG AAGAAGACAA GCAGTCGGTG TCGCGCGCCC

Xyl9a5C TTGCAATTTT AGGCATGGAT GAACTTTCCG AGGACGACAA ACTAGCCGTG TCACGTGCAC

XylTemecul TTGCAATCTT AGGCATGGAT GAACTTTCCG AAGACGACAA ACTATCCGTG TCACGTGCAC

Burkholder TCGCGATTCT GGGCATGGAC GAGCTGTCGC CGGAAGACAA GCTGTCGGTT GCGCGCGCAC

Ralstonia TCGCGATTCT GGGCATGGAC GAACTGTCGC CGGAAGACAA GCTGGCCGTG GGCCGCGCCC

....|....| ....|....| ....|....| ....|....| ....|....| ....|....|

3785 3795 3805 3815 3825 3835

X.albiline GCAAGATCGA GCGCTTCTTC AGCCAGCCGT TCCACGTGGC CGAAGTGTTC ACCGGCTCGC

StenoK279a GTAAGATCGA GCGCTTCTTC AGCCAGCCGT TCCACGTGGC CGAAGTGTTC ACCGGTTCGC

StenoR551 GCAAGATCGA ACGCTTCTTC AGCCAGCCGT TCCACGTGGC CGAAGTGTTC ACCGGCTCGC

Vesicatori GCAAGATCGA GCGCTTCTTC AGCCAGCCTT TCCACGTGGC CGAAGTGTTC ACCGGTTCGC

Citri GCAAGATCGA GCGCTTCTTC AGCCAGCCCT TCCACGTGGC CGAAGTGTTC ACCGGATCGC

Oryzae GCAAGATCGA GCGCTTCTTC AGCCAGCCCT TCCACGTGGC CGAAGTGTTC ACCGGCTCCC

Campestris GCAAGATCGA GCGCTTCTTC AGCCAGCCGT TCCACGTGGC CGAAGTGTTC ACCGGCTCGC

Xyl9a5C GCAAAATAGA ACGATTTTTC AGTCAACCAT TCCATGTCGC AGAAGTATTC ACCGGTGCCC

XylTemecul GCAAAATTGA ACGATTTTTC AGTCAACCAT TCCATGTCGC AGAAGTATTC ACCGGTGCCC

Burkholder GTAAGATCCA GCGTTTCCTG TCGCAGCCGT TCCACGTCGC CGAAGTGTTC ACGGGCTCGC

Ralstonia GTAAGATCCA GCGTTTCCTG TCGCAGCCGT TCCACGTGGC CGAAGTGTTC ACGGGTTCGC

....|....| ....|....| ....|....| ....|....| ....|....| ....|....|

3845 3855 3865 3875 3885 3895

X.albiline CGGGCAAGTA CGTGTCGCTG AAGGACACCA TCCGCGGCTT CAAGGCGATC GTCGATGGCG

StenoK279a CGGGCAAGTA CGTTCCGCTG AAGGACACCA TCCGTGGCTT CAAGGCCATC GTTGATGGCG

StenoR551 CGGGCAAGTA CGTGTCGCTG AAGGACACCA TCCGTGGCTT CAAGGCCATC GTCGATGGCG

Vesicatori CGGGCAAGTA CGTGTCGCTG AAGGACACCA TCCGCGGCTT CAAGGCGATC TGCGACGGCG

Citri CGGGCAAGTA CGTGTCGTTG AAGGACACCA TCCGCGGCTT CAAGGCAATC TGCGACGGCG

Oryzae CGGGCAAGTA CGTCTCGCTG AAGGACACCA TCCGCGGCTT CAAGGCGATC TGCGACGGCG

Campestris CGGGCAAGTA CGTCTCGCTG AAGGACACGA TCCGCGGCTT CAAGGCGATC TGCGACGGTG

Xyl9a5C CAGGTAAATA CGTCCCATTA AAAGAGACAA TCCGTGGTTT CAAAGCAATC GTAGACGGCG

XylTemecul CAGGTAAATA CGTCCCATTA AAAGACACAA TCCGTGGTTT CAAAGCAATC GTAGACGGCG

Burkholder CGGGCAAGTA CGTGCCGCTG AAGGAAACGA TCCGCGGCTT CAAGATGATC GTCGACGGCG

Ralstonia CGGGCAAGTA CGTGCCGCTG AAGGAAACCA TCCGCGGCTT CAAGATGCTG GTGGATGGCG

....|....| ....|....| ....|....| ....|....| ....|....| ....|....|

3905 3915 3925 3935 3945 3955

X.albiline AGTACGACCA CCTGCCGGAG CAGGCGTTCT ACATGGTCGG CGGCATCGAG GAAGCGGTCG

StenoK279a AGTACGACCA CCTGCCGGAG CAGGCGTTCT ACATGGTTGG CGGCATCGAA GAAGCGGTCG

StenoR551 AGTACGACCA CCTGCCGGAG CAGGCGTTCT ACATGGTCGG CAGCATCGAA GAAGCGGTCG

Vesicatori AATACGACCA CCTGCCGGAG CAGGCGTTCT ACATGGTCGG CAGCATCGAA GAAGCGGTCG

Citri AATACGACCA CCTGCCGGAG CAGGCGTTCT ACATGGTCGG CAGCATCGAA GAAGCCGTCG

Oryzae AATACGACCA CCTACCGGAG CAGGCGTTCT ACATGGTCGG CAGCATCGAA GAAGCCGTCG

Campestris AATACGACCA CCTGCCGGAG CAGGCGTTCT ACATGGTCGG CAGCATCGAA GAAGCGGTCG

Xyl9a5C AATATGACCA TCTTCCCGAA CAAGCCTTCT ATATGGTTGG TAACATTGAA GAAGTCATCG

XylTemecul AATATGACCA TCTTCCCGAA CAAGCCTTCT ATATGGTCGG TAACATTGAA GAAGTCATCG

Burkholder AGTGCGACCA CCTGCCGGAA CAGGCGTTCT ACATGGTCGG CACGATCGAC GAAGCCTTCG

Ralstonia AGTGCGATCA CCTGCCGGAG CAGGCGTTCT ACATGGTCGG CTCGATCGAC GAGGCCTTCG

....|....| ....|....| ....|....| ....|....| ....|....| ....|....|

3965 3975 3985 3995 4005 4015

X.albiline AGAAAGCCAA GAAGATGGCC GAAAAGGCCT GA-------- ---------- ----------

StenoK279a AGAAGGCCAA GAAGATGGCC GAGAAGGCCT GA-------- ---------- ----------

StenoR551 AGAAGGCCAA GAAGATGGCC GAGAAGGCCT GA-------- ---------- ----------

Vesicatori AGAAAGCCAA CAAGATGA-- ---------- ---------- ---------- ----------

Citri AGAAGGCCAA CAAGATGA-- ---------- ---------- ---------- ----------

Oryzae AGAAAGCCAA CAAGATGAGC GCCAAGGCGT AA-------- ---------- ----------

Campestris AGAAGGCCAA CAAGATGAGC GCCAAGGCCT GA-------- ---------- ----------

Xyl9a5C AAAAAGCCAA CAAAATGA-- ---------- ---------- ---------- ----------

XylTemecul AAAAAGCCAA CAAAATGA-- ---------- ---------- ---------- ----------

Burkholder AGAAGGCCAA GAAGATCCAG TAAATG---- ---------- ---------- ----------

Ralstonia AGAAGGCCAA GAAGCTCCAG TAAATGCTCA ACTTTTCAAA AATCGATCTT GAAAAGGCCG

....|....| ....|....| ....|....| ....|....| ....|....| ....|....|

4025 4035 4045 4055 4065 4075

X.albiline ---------- ---------- ---------- ---------- ---------- ----------

StenoK279a ---------- ---------- ---------- ---------- ---------- ----------

StenoR551 ---------- ---------- ---------- ---------- ---------- ----------

Vesicatori ---------- ---------- ---------- ---------- ---------- ----------

Citri ---------- ---------- ---------- ---------- ---------- ----------

Oryzae ---------- ---------- ---------- ---------- ---------- ----------

Campestris ---------- ---------- ---------- ---------- ---------- ----------

Xyl9a5C ---------- ---------- ---------- ---------- ---------- ----------

XylTemecul ---------- ---------- ---------- ---------- ---------- ----------

Burkholder ---------- ---------- ---------- ---------- ---------- ----------

Ralstonia GGAGGCACTC CCACATCCGA GCCAAGTTTG TATTCAGTGC AGTCCTGAGA CCAGATTCGA

....|....| ....|....| ....|....| ....|....| ....|....| ....|....|

4085 4095 4105 4115 4125 4135

X.albiline ---------- -ATGGGCAAG ATCATCGGCA TCGACCTGGG CACGACCAAT TCGTGCGTGG

StenoK279a ---------- -ATGGGCAAG ATCATTGGTA TCGACCTCGG CACCACCAAC TCGTGCGTGG

StenoR551 ---------- -ATGGGCAAG ATCATTGGTA TCGACCTCGG CACCACCAAC TCGTGCGTGG

Vesicatori ---------- -ATGGGCAAG ATCATTGGTA TTGACCTCGG CACCACGAAC TCGTGCGTGT

Citri ---------- -ATGGGCAAG ATCATTGGTA TTGACCTCGG CACCACGAAC TCGTGCGTGT

Oryzae ---------- -ATGGGCAAG ATCATTGGTA TTGACCTCGG CACCACGAAC TCGTGCGTGT

Campestris ---------- -ATGGGCAAG ATCATTGGTA TTGACCTCGG CACCACGAAC TCGTGCGTGG

Xyl9a5C ---------- -ATGGGCAAA ATCATTGGTA TCGACCTCGG CACCACAAAT TCGTGCTTAG

XylTemecul ---------- -ATGGGCAAA ATCATTGGTA TCGACCTCGG CACCACAAAT TCGTGCTTAG

Burkholder ---------- ----GGAAAG ATCATCGGTA TTGACCTCGG CACCACGAAC TCGTGCGTCG

Ralstonia GGAGCAAGAA CATGGGCAAA ATCATCGGTA TCGACCTGGG TACCACCAAC AGCTGCGTGT

....|....| ....|....| ....|....| ....|....| ....|....| ....|....|

4145 4155 4165 4175 4185 4195

X.albiline CGATCATGGA CGGCGGCAAG GCTCGCGTCA TCGAGAATTC CGAGGGCGAC CGCACCACGC

StenoK279a CGATCATGGA CGGCGGCAAG GCCCGCGTCA TCGAGAATTC GGAAGGCGAT CGCACCACCC

StenoR551 CGATCATGGA CGGCGGCAAG GCCCGCGTCA TCGAGAATTC GGAAGGCGAT CGCACCACAC

Vesicatori CGATCATGGA CGGCGGCAAG GCCCGCGTCA TCGAAAACTC CGAGGGCGAT CGCACCACGC

Citri CGATCATGGA CGGCGGCAAG GCCCGCGTCA TCGAAAACTC CGAGGGCGAT CGCACCACGC

Oryzae CGATCATGGA CGGCGGCAAG GCCCGCGTCA TCGAAAACTC CGAGGGCGAT CGCACCACGC

Campestris CGATCATGGA CGGCGGCAAG GCCCGCGTCA TCGAAAATTC CGAGGGCGAT CGCACCACGC

Xyl9a5C CGATTATTGA AGGCGGTAAA GGGCGTGTCA TCGAAAACTC GGAGGGTGAT CGGACCACTC

XylTemecul CGATTATTGA AGGCGGTAAA GGGCGTGTCA TCGAAAACTC GGAGGGTGAT CGGACCACTC

Burkholder CGATCATGGA AGGCAACCAG GTCAAGGTCA TCGAGAATTC GGAAGGCGCG CGCACGACGC

Ralstonia CCATCATGGA GGGCAACACG CCCAAGGTGA TCGAGAACGC GGAAGGCGCG CGCACCACGC

....|....| ....|....| ....|....| ....|....| ....|....| ....|....|

4205 4215 4225 4235 4245 4255

X.albiline CTTCGATCGT CGCCTACACC AAGGACGGCG AAGTGTTGGT GGGCGCCTCG GCCAAGCGCC

StenoK279a CGTCGATCGT CGCCTACACC AAGGACGGCG AAGTCCTGGT CGGTGCCTCG GCCAAGCGCC

StenoR551 CGTCGATCGT CGCCTACACC AAGGACGGCG AAGTCCTGGT GGGTGCCTCG GCCAAGCGCC

Vesicatori CTTCGATCGT CGCCTACACC AAGGACGGCG AAGTGCTGGT CGGCGCCTCG GCCAAGCGCC

Citri CTTCGATCGT CGCCTACACC AAGGACGGCG AAGTGCTGGT CGGCGCTTCG GCCAAGCGCC

Oryzae CCTCGATCGT CGCCTACACC AAGGACGGCG AAGTGCTGGT AGGCGCCTCG GCCAAGCGCC

Campestris CCTCGATCGT CGCCTACACC AAGGACGGCG AAGTGCTGGT CGGTGCCTCG GCCAAGCGCC

Xyl9a5C CTTCCATTGT TGCTTACACC AAGGATGGTG AAGTGCTTGT TGGTGCTGCA GCCAAGCGCC

XylTemecul CTTCCATTGT TGCTTACACC AAGGATGGTG AAGTGCTTGT TGGTGCTGCA GCCAAGCGCC

Burkholder CGTCGATCAT CGCGTACATG GACGACAACG AAGTGCTCGT CGGCGCGCCC GCCAAGCGCC

Ralstonia CGTCGATCAT CGCTTACATG GAAGACGGCG AGATCCTGGT CGGCGCCCCC GCCAAGCGCC

....|....| ....|....| ....|....| ....|....| ....|....| ....|....|

4265 4275 4285 4295 4305 4315

X.albiline AGGCGGTGAC CAACCCGAAG AACACCTTCC ATGCGGTCAA GCGTCTGATC GGCCGCAAGT

StenoK279a AGGCCGTGAC CAACCCGAAG AACACCTTCT ACGCGGTGAA GCGCCTGATC GGCCGCAAGT

StenoR551 AGGCCGTCAC CAACCCCAAG AACACCTTCT ACGCGGTGAA GCGCCTGATC GGCCGCAAGT

Vesicatori AGGCCGTGAC CAACCCGAAG AACACCTTCT ACGCGGTGAA GCGCCTGATC GGCCGCAAGT

Citri AGGCCGTGAC CAACCCGAAG AACACCTTCT ACGCGGTGAA GCGCCTGATC GGCCGCAAGT

Oryzae AGGCGGTGAC CAACCCGAAG AACACCTTCT ACGCGGTGAA GCGCCTGATC GGCCGCAAGT

Campestris AGGCGGTGAC CAACCCGAAG AACACCTTCT ACGCGGTGAA GCGCCTGATC GGCCGCAAGT

Xyl9a5C AAGCGGTCAC AAATCCGAAA AACACCTTTT ATGCGGTTAA GCGTTTGATT GGACGTAAGT

XylTemecul AAGCGGTCAC AAATCCGAAA AACACCTTTT ATGCGGTTAA GCGTTTGATT GGACGTAAGT

Burkholder AATCGGTGAC GAACCCGAAG AACACGCTGT TCGCAGTCAA GCGCCTGATC GGCCGCCGTT

Ralstonia AGGCCGTCAC CAACCCGAGG AACACGCTGT ACGCCGTCAA GCGCCTGATC GGCCGCAAGT

....|....| ....|....| ....|....| ....|....| ....|....| ....|....|

4325 4335 4345 4355 4365 4375

X.albiline TCGGCGATGC CGAAGTGCAG AAGGACATCG GCCTGGTGCC TTACGCCATC GCTCAGCACG

StenoK279a TCACCGACGC CGAAGTGCAG AAGGACATCG CGCACGTCCC GTACAGCATC CTGGCCCATG

StenoR551 TCACCGACGC CGAAGTGCAG AAGGACATCG CGCACGTCCC GTACAGCATC CTGGCGCATG

Vesicatori TCACCGATGG CGAAGTGCAG AAGGACATCT CGCACGTGCC GTACGGCATC CTGGCGCACG

Citri TCACCGACGG CGAAGTGCAG AAGGACATCT CGCACGTGCC GTACGGCATC CTGGCGCACG

Oryzae TCACCGACGC CGAAGTGCAG AAGGACATCT CGCACGTGCC GTACGGCATC CTGGCGCACG

Campestris TCACTGACGG CGAAGTGCAG AAGGACATCT CCCACGTGCC GTACGGCATT CTGGCGCACG

Xyl9a5C TCGGTGATGC AGAGGTCCAA AAGGACCTTG ATTTGGTGCC GTATAAGATT ACTCAGCATG

XylTemecul TCGGTGATGC AGAGGTCCAA AAGGACCTTG ATTTGGTGCC GTATAAGATT ACTCAGCATG

Burkholder TCGAAGGGAA GGAAGTCCAG AAGGACATCG GCCTGATGCC GTACGCGATC ATCAAGGCGG

Ralstonia TCGAAGAGAA GGAAGTCCAG AAGGACATCG GCCTGATGCC GTACACCATC TCCAAGGCCG

....|....| ....|....| ....|....| ....|....| ....|....| ....|....|

4385 4395 4405 4415 4425 4435

X.albiline ACAACGGCGA TGCCTGGGTG GCCACTGCCG ATGGACGCAA GCTGGCGCCG CAGGAAATTT

StenoK279a ACAATGGCGA CGCCTGGGTG GCCACCAGCG ACGGCAAGAA GATGGCCCCG CAGGAAATCT

StenoR551 ACAATGGCGA TGCCTGGGTG GCCACCAGCG ATGCCAAGAA GATGGCGCCG CAGGAAATCT

Vesicatori ACAACGGCGA CGCCTGGGTG CAGACCAGCG ATGCCAAGCG CATGGCGCCG CAGGAAATCT

Citri ACAATGGCGA CGCCTGGGTG CAGACCAGCG ACGGCAAGCG CATGGCGCCG CAGGAAATCT

Oryzae ACAACGGCGA CGCCTGGGTG CAGACCAGCG ATGCCAAGCG CATGGCGCCG CAGGAAATCT

Campestris ACAACGGCGA TGCCTGGGTG CAGACCAGCG ATTCCAAGCG CATGGCGCCG CAGGAAATTT

Xyl9a5C ACAATGGGGA TGCCTGGGTG GCCACTGCTG ATGCTAAGAA GCTGGCGCCG CAGGAAATCT

XylTemecul ACAATGGGGA TGCCTGGGTG GCCACTGCTG ATGGTAAGAA GCTGGCGCCG CAGGAAATCT

Burkholder ACAACGGCGA CGCGTGGGTC GAGGCGCACG GCG---AGAA GCTCGCGCCG CCGCAGGTGT

Ralstonia ACAACGGCGA CGCCTGGGTG GAAGTGCGCG ACA---AGAA GATGGCGCCG CCGCAGATTT

....|....| ....|....| ....|....| ....|....| ....|....| ....|....|

4445 4455 4465 4475 4485 4495

X.albiline CCGCACAGGT GCTGGAAAAG ATGAAAAAGA CCGCCGAGGC GTTCCTTGGC GAGAAGGTGA

StenoK279a CGGCCAAGGT GCTGGAAAAG ATGAAGAAGA CCGCCGAGGA CTTCCTCGGT GAGAAGGTCA

StenoR551 CGGCCAAGGT GCTGGAAAAG ATGAAGAAGA CCGCCGAGGA CTTCCTCGGT GAGAAGGTCA

Vesicatori CCGCGCGCGT GCTGGAGAAG ATGAAGAAGA CCGCCGAAGA CTTCCTCGGC GAGAAGGTCA

Citri CCGCGCGCGT GCTGGAAAAG ATGAAGAAGA CCGCCGAAGA CTTCCTCGGC GAGAAGGTCA

Oryzae CCGCACGCGT GCTGGAGAAA ATGAAGAAGA CCGCGGAAGA CTACCTCGGC GAGAAGGTCA

Campestris CCGCACGCGT GCTGGAAAAG ATGAAGAAGA CCGCCGAGGA CTTCCTGGGC GAAAAGGTCA

Xyl9a5C CTGCCAAGGT GCTAGAGAAA ATGAAGAAGA CCGCCGAGGA TTTCCTGGGC GAGAAAGTCA

XylTemecul CTGCCAAGGT GCTGGAGAAA ATGAAGAAGA CCGCCGAGGA TTTCCTGGGC GAGAAAGTCA

Burkholder CGGCCGAAGT GCTGCGCAAG ATGAAGAAGA CGGCCGAAGA CTACCTCTGC GAGCCGGTCA

Ralstonia CGGCCGAAGT CCTGCGCAAG ATGAAGAAGA CCGCCGAGGA CTACCTGGGC GAGGAAGTGA

....|....| ....|....| ....|....| ....|....| ....|....| ....|....|

4505 4515 4525 4535 4545 4555

X.albiline CCGAAGCGGT CATCACCGTG CCGGCGTACT TCAACGATAG CCAGCGTCAG GCGACCAAGG

StenoK279a CCGAAGCGGT CATCACCGTG CCGGCCTACT TCAACGACAG CCAGCGCCAG GCAACCAAGG

StenoR551 CCGAAGCGGT CATCACCGTG CCGGCCTACT TCAACGACAG CCAGCGCCAG GCAACCAAGG

Vesicatori CCGAGGCGGT GATCACCGTG CCGGCATACT TCAACGACAG CCAGCGTCAG GCCACCAAGG

Citri CCGAGGCGGT GATCACGGTG CCGGCGTACT TCAACGACAG CCAGCGTCAG GCCACCAAGG

Oryzae CCGAAGCGGT GATCACGGTG CCGGCGTACT TCAACGACAG CCAGCGTCAG GCCACCAAGG

Campestris CCGAAGCGGT GATCACGGTG CCGGCGTACT TCAACGACAG CCAGCGCCAG GCCACCAAGG

Xyl9a5C CCGAAGCGGT GATTACCGTG CCGGCGTATT TTAATGACAG TCAGCGTCAG GCGACGAAAG

XylTemecul CCGAAGCGGT GATTACCGTG CCGGCATATT TTAATGACAG TCAGCGTCAG GCAACGAAAG

Burkholder CGGAAGCCGT GATCACGGTG CCGGCGTACT TCAACGACAG CCAGCGCCAG GCGACGAAGG

Ralstonia CCGAAGCCGT GATCACGGTG CCGGCCTACT TCAACGATTC GCAGCGCCAG GCGACCAAGG

....|....| ....|....| ....|....| ....|....| ....|....| ....|....|

4565 4575 4585 4595 4605 4615

X.albiline ACGCCGGTCG CATCGCCGGC CTGGACGTCA AGCGCATCAT CAACGAGCCT ACAGCGGCGG

StenoK279a ACGCCGGCCG CATCGCCGGC CTGGACGTCA AGCGCATCAT CAACGAGCCG ACCGCGGCCG

StenoR551 ACGCAGGTCG CATCGCCGGC CTGGACGTCA AGCGCATCAT CAATGAGCCG ACTGCGGCCG

Vesicatori ATGCCGGCCG CATCGCCGGT CTGGACGTCA AGCGCATCAT CAACGAGCCG ACCGCCGCAG

Citri ATGCCGGCCG CATCGCCGGT CTGGACGTCA AGCGCATCAT CAACGAGCCG ACCGCCGCAG

Oryzae ACGCTGGCCG CATCGCCGGT CTGGACGTCA AGCGCATCAT CAACGAGCCG ACCGCCGCAG

Campestris ACGCTGGCCG CATCGCCGGT CTGGACGTCA AGCGCATCAT CAACGAGCCG ACGGCTGCTG

Xyl9a5C ATGCTGGTCG GATCGCTGGC CTGGATGTCA AACGTATTAT CAACGAGCCG ACCGCTGCGG

XylTemecul ATGCTGGTCG GATCGCTGGC CTGGATGTCA AACGTATTAT CAACGAGCCG ACCGCTGCGG

Burkholder ACGCGGGGCG CATCGCGGGC CTCGAAGTCA AGCGGATCAT CAACGAGCCG ACCGCGGCCG

Ralstonia ACGCCGGCCG CATCGCGGGC CTGGACGTCA AGCGCATCAT CAACGAGCCG ACCGCGGCCG

....|....| ....|....| ....|....| ....|....| ....|....| ....|....|

4625 4635 4645 4655 4665 4675

X.albiline CGCTTGCATA TGGCTTGGAC AAGGCTCACG GCGGTGATCG CAAGATTGCC GTGTACGACC

StenoK279a CGCTGGCCTA TGGCCTGGAC AAGGGCGACA ACAAGGATCG CAAGATCGTG GTGTACGACC

StenoR551 CGCTGGCCTA TGGCCTGGAC AAGGGCGACA ACAAGGATCG CAAGATCGTG GTGTACGACC

Vesicatori CCCTGGCCTA TGGCCTGGAC AAGAACGGCG GC---GACCG CAAGATAGCC GTGTACGACC

Citri CGCTGGCCTA TGGCCTGGAC AAGAACGGCG GC---GACCG CAAGATTGCC GTGTACGACC

Oryzae CACTGGCTTA TGGCCTGGAC AAGAAGGGCG GC---GACCG CAAGATTGCG GTGTACGACC

Campestris CCCTGGCCTA TGGCCTGGAC AAGAACGGCG GT---GACCG CAAGATTGCC GTGTACGACC

Xyl9a5C CATTGGCTTA CGGTTTGGAC AAGAAGGGCG GA---GATCG CAAGATCGCT GTATATGATC

XylTemecul CATTGGCTTA CGGTTTGGAC AAGAAGGGCG GA---GATCG CAAGATCGCT GTATATGATC

Burkholder CGCTCGCGTT CGGCCTCGAC AAGGCCGAGA AGGGCGATCG CAAGATCGCG GTGTATGACC

Ralstonia CGCTGGCCTT CGGCCTGGAC AAGAACGAGA AGGGCGACCG CAAGATCGCG GTGTATGACC

....|....| ....|....| ....|....| ....|....| ....|....| ....|....|

4685 4695 4705 4715 4725 4735

X.albiline TGGGTGGCGG CACCTTCGAC GTGTCGATCA TCGAAATCGC CAATGTCGAC GGCGAAAAGC

StenoK279a TGGGCGGCGG CACCTTCGAC GTCTCGGTGA TCGAGATCGC CAACGTCGAC GGTGAAAAGC

StenoR551 TGGGCGGCGG CACCTTCGAC GTTTCGGTGA TCGAGATCGC CAACGTCGAT GGTGAAAAGC

Vesicatori TGGGCGGCGG TACCTTCGAC GTGTCGATTA TCGAGATCGC CGAAGTCGAT GGTGAAAAGC

Citri TGGGCGGCGG CACCTTCGAC GTCTCGATCA TCGAAATCGC TGAAGTCGAT GGCGAGAAGC

Oryzae TGGGCGGCGG CACCTTCGAC GTGTCGATCA TCGAGATCGC TGAAGTCGAT GGCGAAAAGC

Campestris TGGGCGGCGG CACCTTCGAC GTGTCGATCA TCGAAATTGC CGAGGTCGAT GGCGAGAAGC

Xyl9a5C TTGGCGGCGG TACCTTTGAC GTCTCGATCA TTGAAATTGC TGAAGTGGAT GGTGAGAAGC

XylTemecul TTGGCGGCGG TACCTTTGAC GTCTCAATCA TTGAAATTGC TGAAGTGGAT GGTGAGAAGC

Burkholder TCGGCGGCGG CACGTTCGAC GTGTCGATCA TCGAGATCGC GGACGTCGAC GGCGAAATGC

Ralstonia TGGGCGGCGG CACGTTCGAC ATCTCGATCA TCGAGATCGC CGACGTGGAC GGCGAGAAGC

....|....| ....|....| ....|....| ....|....| ....|....| ....|....|

4745 4755 4765 4775 4785 4795

X.albiline AGTTCGAAGT GCTAGCCACC AATGGCGACA CCTTCCTGGG TGGCGAGGAT TTCGACAAGC

StenoK279a AGTTCGAAGT GCTGGCCACC AACGGCGACA CGTTCCTGGG TGGCGAAGAC TTCGACAACC

StenoR551 AGTTCGAAGT GCTGGCGACC AACGGTGACA CCTTCCTGGG CGGCGAAGAT TTCGACAACC

Vesicatori AGTTCGAAGT GCTGGCCACC AACGGCGACA CCTTCCTGGG CGGCGAAGAC TTCGACAACC

Citri AGTTCGAAGT GCTGGCCACC AATGGCGACA CTTTCCTGGG CGGCGAAGAT TTCGACAACC

Oryzae AGTTCGAAGT GCTGGCTACC AACGGCGATA CCTTCCTGGG CGGCGAAGAC TTCGACAACC

Campestris AGTTCGAAGT GCTGGCCACC AATGGCGACA CCTTCCTGGG CGGCGAAGAC TTCGACAACC

Xyl9a5C AGTTCGAGGT ATTGGCGACC AATGGTGATA CCTTCTTGGG AGGTGAAGAT TTCGACAAGC

XylTemecul AGTTCGAGGT ATTGGCGACC AATGGTGATA CCTTCTTGGG AGGTGAGGAT TTCGACAAGC

Burkholder AGTTCGAAGT GCTGTCGACC AACGGCGACA CGTTCCTCGG CGGCGAGGAC TTCGACCAGC

Ralstonia AGTTCGAAGT GCTGTCGACC AACGGCGACA CCTTCCTGGG CGGCGAAGAC TTCGACCAGC

....|....| ....|....| ....|....| ....|....| ....|....| ....|....|

4805 4815 4825 4835 4845 4855

X.albiline GCGTCATCGA CTATCTGGTG GACGAGTTCA ACAAAGATCA AGGCATCGAT CTGCGCAAGG

StenoK279a GCGTCATCGA GTACCTGGTT GAAGAGTTCA ACAAGGACCA GGGCATCGAC CTGCGCAAGG

StenoR551 GCGTCATCGA GTACCTGGTT GAAGAGTTCA ACAAGGACCA GGGCATCGAC CTGCGCAAGG

Vesicatori GCGTCATCGA GTACCTGGTC GACGAATTCA ACAAGGACCA GGGCATCGAC CTGCGTAAGG

Citri GCGTCATCGA GTACCTGGTC GATGAATTCA ACAAGGATCA GGGTATCGAT CTGCGCAAGG

Oryzae GCGTCATCGA GTACCTGGTC GATGAGTTCA ACAAGGACCA GGGCATCGAC CTGCGCAAGG

Campestris GCGTCATCGA GTATCTGGTC GACGAATTCA ACAAGGATCA GGGCATCGAT CTGCGCAAGG

Xyl9a5C GTGTTATCGA TTATCTGGTT GATGAATTCA ATAAGGATCA GGGGATCGAT TTGCGCAAGG

XylTemecul GTGTTATTGA TTATCTGGTT GATGAATTCA ATAAGGATCA GGGGATTGAT TTGCGCAAGG

Burkholder GCATCATCGA TTACATCATC GGCGAATTCA AGAAGGAGCA GGGCGTCGAT CTCTCGAAGG

Ralstonia GCATCATCGA TTACATCATC GGCGAGTTCA AGAAGGAGTC GGGCGTCGAC CTGTCGAAGG

....|....| ....|....| ....|....| ....|....| ....|....| ....|....|

4865 4875 4885 4895 4905 4915

X.albiline ATCCGTTGGC GCTACAGCGC CTGAAGGATG CCGCCGAGCG TGCCAAGATC GAACTCTCGT

StenoK279a ATCCGCTGGC CCTGCAGCGC CTGAAGGATG CTGCCGAGCG CGCCAAGATC GAGCTGTCCA

StenoR551 ATCCGCTGGC CCTGCAGCGC CTGAAGGATG CTGCCGAGCG CGCCAAGATC GAACTGTCCA

Vesicatori ATCCGCTGGC GCTGCAGCGC CTGAAGGACG CCGCAGAGCG CGCCAAGATC GAGCTGTCGT

Citri ATCCGTTGGC GCTGCAGCGC CTGAAGGACG CCGCAGAGCG CGCCAAGATC GAGCTGTCGT

Oryzae ATCCGCTCGC GTTGCAGCGC CTGAAGGACG CCGCAGAGCG CGCCAAGATC GAGCTGTCGT

Campestris ATCCGTTGGC GCTGCAGCGC CTCAAGGACG CCGCAGAGCG CGCCAAGATC GAGCTGTCGA

Xyl9a5C ATCCGCTGGC GTTACAACGT TTAAAGGATG CTGCTGAGCG CGCTAAAATC GAATTGTCTT

XylTemecul ATCCGCTTGC GTTGCAACGT TTAAAGGATG CTGCTGAGCG CGCTAAAATC GAATTGTCTT

Burkholder ACGTGCTCGC GCTGCAGCGC CTGAAGGAAG CGGCCGAGAA GGCGAAGATC GAGCTGTCGT

Ralstonia ACGTGCTTGC GCTGCAACGC CTGAAGGATG CCGCCGAGAA GGCCAAGATC GAGCTGTCGT

....|....| ....|....| ....|....| ....|....| ....|....| ....|....|

4925 4935 4945 4955 4965 4975

X.albiline CCTCGCAGCA GACCGAAGTC AACTTGCCTT ACGTCACCGC CGACGCGTCG GGTCCGAAGC

StenoK279a GCGCCCAGCA GACCGAAGTG AACCTGCCGT ACGTCACCGC TGACGCGTCG GGTCCGAAGC

StenoR551 GCGCCCAGCA GACCGAAGTG AACCTGCCGT ACGTCACCGC TGACGCGTCG GGTCCGAAGC

Vesicatori CCAGCCAGCA GACCGAAGTG AACCTGCCGT ACGTCACCGC CGATGCGTCG GGCCCGAAGC

Citri CCAGCCAGCA GACCGAAGTG AACCTGCCGT ACGTCACCGC CGATGCCTCG GGCCCGAAGC

Oryzae CCAGCCAGCA GACCGAAGTC AATCTGCCGT ACGTCACCGC CGATGCGTCG GGCCCGAAGC

Campestris CCTCGCAGCA GACCGAAGTG AACCTGCCGT ACGTCACCGC CGATGCCTCG GGCCCGAAGC

Xyl9a5C CCTCCCAGCA AACCGAAGTT AACCTGCCAT ACATTACGGC GGATGCATCG GGTCCGAAGC

XylTemecul CATCCCAGCA AACCGAAGTT AACCTGCCAT ACATTACGGC GGATGCATCG GGTCCGAAGC

Burkholder CGAGCCAGCA GACCGAAATC AACCTGCCGT ACATCACGGC CGACGCGTCG GGCCCGAAGC

Ralstonia CGACGCAACA GACCGAGATC AACCTGCCGT ACATCACGGC CGATGCCTCG GGCCCGAAGC

....|....| ....|....| ....|....| ....|....| ....|....| ....|....|

4985 4995 5005 5015 5025 5035

X.albiline ATCTGAACAT CAAGCTGACC CGGGCCAAGC TCGAGGCGCT GGTGGAAGAT CTGGTCAAGC

StenoK279a ACCTGAACAT CAAGCTGACC CGCGCCAAGC TGGAAGCCCT GGTGGACGAC CTGATCAAGA

StenoR551 ACCTGAACAT CAAGTTGACC CGCGCCAAGC TGGAGTCGCT GGTGGAAGAG CTGATCAGGA

Vesicatori ACCTCAACAT CAAGTTGACC CGTGCCAAGC TCGAAGCGCT GGTGGAAGAC CTGGTCAAGA

Citri ACCTCAACAT CAAGTTGACC CGTGCCAAGC TCGAAGCGCT GGTGGAAGAC CTGGTCAAGA

Oryzae ACCTCAACAT CAAGTTGACC CGTGCCAAGC TCGAAGCGCT GGTGGAAGAC CTGGTCAAGA

Campestris ACCTCAACAT CAAGTTGACC CGTGCCAAGC TCGAAGCGCT GGTGGAAGAC CTGGTCAAGA

Xyl9a5C ACCTGAATAT CAAACTGACT CGTGCCAAGC TTGAAGCCTT GGTAGACGAT TTGGTCCGTA

XylTemecul ACCTGAATAT CAAACTGACT CGTGCCAAGC TTGAAGCCTT GGTAGACGAC TTGGTCCGTA

Burkholder ACTTGAACCT GAAGGTCACG CGCGCGAAGC TCGAGGCGCT CGTCGAGGAT CTGGTCGAGC

Ralstonia ACTTGAACCT GAAGATCACG CGCGCCAAGC TCGAAGCGCT GGTCGAAGAC CTGATCGCTC

....|....| ....|....| ....|....| ....|....| ....|....| ....|....|

5045 5055 5065 5075 5085 5095

X.albiline GCACCATCGA CCCGTGCCGC ACCGCGTTGA ACGATGCCGG CTTGCGCGCC AGCGACATCA

StenoK279a AGTCGATCGA GCCGTGCCGC GTCGCCCTGA ACGATGCCGG CCTGCGTTCG AGCGACATCA

StenoR551 AGTCGATCGA GCCGTGCCGC GTCGCCCTGA ACGATGCCGG CCTGCGTTCG AGCGACATCA

Vesicatori AGTCGATCGA ACCGTGCCGC ACCGCGTTGA ACGACGCCGG CCTGCGCGCC AGCGACATCA

Citri AGTCGATCGA GCCGTGCCGC ACCGCGTTGA ACGACGCCGG CCTGCGCGCC AGCGACATCA

Oryzae AGTCGATCGA GCCGTGCCGC ACCGCGTTGA ACGACGCCGG CCTGCGCGCC AGCGACATCA

Campestris AGTCGATCGA GCCGTGCCGC ACCGCGTTGA ACGACGCCGG CCTGCGCGCC AGCGACATCA

Xyl9a5C AGTCAATTGA GCCATGCCGC ATTGCGTTGA ACGACGCTGG TTTGCGTACC AGTGATGTTC

XylTemecul AGTCAATTGA GCCATGCCGC ATTGCGTTGA ACGACGCTGG GTTGCGTACC AGTGATGTTC

Burkholder GCACGATCGA GCCGTGCCGC ACCGCGATCA AGGACGCGGG CGTCAAGGTG TCGGACATCG

Ralstonia GCACGATCGA GCCGTGCCGC ACCGCCATCA AGGACGCCGG CGTGAAGGTG TCGGACATCC

....|....| ....|....| ....|....| ....|....| ....|....| ....|....|

5105 5115 5125 5135 5145 5155

X.albiline CCGAGGTGAT CCTGGTCGGT GGCCAGACCC GCATGCCCAA GGTGCAGCAG GCCGTGGCCG

StenoK279a GCGAAGTGAT CCTGGTCGGC GGCCAGACCC GCATGCCGAA GGTGCAGCAG GCGGTGACCG

StenoR551 GCGAAGTGAT CCTGGTCGGC GGCCAGACCC GCATGCCGAA GGTGCAGCAG GCGGTGACCG

Vesicatori ACGAAGTGAT CCTGGTCGGC GGCCAGACCC GCATGCCGAA GGTGCAGCAG GCGGTTGCCG

Citri ACGAAGTGAT CCTGGTCGGC GGCCAGACCC GTATGCCGAA GGTGCAGCAG GCGGTTGCCG

Oryzae ACGAAGTGAT CCTGGTCGGC GGTCAGACCC GCATGCCGAA GGTGCAGCAG GCGGTTGCCG

Campestris ACGAAGTGAT CCTGGTCGGC GGTCAGACCC GTATGCCGAA GGTGCAGCAG GCCGTTGCCG

Xyl9a5C AAGAAGTGAT TTTGGTTGGT GGCCAGACCC GTATGCCGAA GGTCCAGCAA GCGGTTGCCG

XylTemecul AAGAAGTGAT TTTGGTTGGC GGCCAGACCC GTATGCCGAA GGTCCAGCAA GCGGTTGCCG

Burkholder ACGACGTGAT CCTCGTCGGC GGCCAGACCC GCATGCCGAA GGTGCAGGAG AAGGTGAAGG

Ralstonia ATGACGTGAT CCTGGTCGGC GGCATGACGC GCATGCCGAA GGTGCAGGAG AAGGTGAAGG

....|....| ....|....| ....|....| ....|....| ....|....| ....|....|

5165 5175 5185 5195 5205 5215

X.albiline AGTTCTTCGG CAAGGATCCG CGCAAGGACG TCAACCCGGA CGAGGCCGTG GCGTTGGGCG

StenoK279a AGTTCTTCGG CAAGGAACCG CGCAAGGACG TCAACCCGGA CGAAGCCGTG GCACTGGGTG

StenoR551 AGTTCTTCGG CAAGGAACCG CGCAAGGACG TCAACCCGGA CGAAGCCGTG GCACTGGGTG

Vesicatori ATTTCTTCGG CAAGGAACCG CGCAAGGACG TCAACCCGGA CGAAGCGGTG GCCGTGGGTG

Citri ATTTCTTCGG CAAGGAACCG CGCAAGGACG TCAACCCGGA CGAAGCCGTG GCCGTGGGTG

Oryzae ATTTCTTCGG CAAGGAACCG CGCAAGGATG TCAACCCGGA CGAAGCCGTG GCCGTGGGTG

Campestris ATTTCTTCGG CAAGGAACCG CGCAAGGACG TCAACCCGGA CGAAGCCGTG GCCGTGGGCG

Xyl9a5C ACTTCTTTGG TAAAGAGCCG CGTAAGGACG TTAATCCGGA CGAAGCGGTT GCATTGGGTG

XylTemecul ACTTCTTTGG TAAAGAGCCG CGTAAGGACG TTAATCCGGA CGAAGCAGTT GCATTGGGTG

Burkholder AATTCTTCGG CAAGGAGCCG CGCCGCGACG TGAACCCGGA CGAAGCCGTC GCGGTGGGCG

Ralstonia AGTTCTTCGG CAAGGAAGCC CGCAAGGACG TGAACCCGGA CGAGGCTGTT GCCGTGGGCG

....|....| ....|....| ....|....| ....|....| ....|....| ....|....|

5225 5235 5245 5255 5265 5275

X.albiline CGGCGATCCA GGGCGGTGTG CTGGCCGGCG ACGTCAAGGA CGTCTTGCTG CTCGACGTGA

StenoK279a CTGCGATCCA GGGCGGCGTG CTGGGCGGCG ACGTCAAGGA CGTGCTGCTG CTGGACGTGA

StenoR551 CTGCGATCCA GGGCGGCGTG CTGGGCGGCG ACGTCAAGGA CGTGCTGCTG CTGGACGTGA

Vesicatori CGGCGATCCA GGGCGGCGTG CTGGCCGGCG ACGTCAAGGA CGTGCTGCTG CTGGACGTGA

Citri CGGCGATCCA GGGCGGCGTG CTGGCCGGCG ACGTCAAGGA CGTGCTGCTG CTGGACGTGA

Oryzae CTGCGATTCA GGGCGGCGTG CTGGCCGGCG ACGTCAAGGA CGTGCTGCTG CTGGACGTGA

Campestris CCGCGATCCA GGGCGGCGTG CTGGCTGGCG ACGTCAAGGA CGTGCTGCTG CTGGACGTGA

Xyl9a5C CTGCGATTCA GGGTGGGGTG CTGGCTGGTG ACGTGAAGGA TGTGTTACTG CTGGATGTGA

XylTemecul CTGCAATTCA GGGTGGGGTG CTGGCTGGTG ACGTGAAGGA TGTGTTACTG CTAGATGTGA

Burkholder CGGCGATCCA GGGCCAGGTG CTTTCGGGCG ACCGCAAGGA CGTGCTGCTG CTCGACGTGA

Ralstonia CCGCCATCCA GGGCCAGGTG CTGGGCGGCG ACCGCAAGGA CGTGCTGCTG CTGGACGTGA

....|....| ....|....| ....|....| ....|....| ....|....| ....|....|

5285 5295 5305 5315 5325 5335

X.albiline CCCCGCTGAG CCTGGGGATC GAGACCCTGG GTGGCGTGTT CACCAAGATC ATCGAGAAGA

StenoK279a CCCCGCTGTC GCTGGGCATC GAGACCATGG GCGGCGTGTT CACCAAGATC ATCGAGAAGA

StenoR551 CCCCGCTGTC GCTGGGTATC GAAACCATGG GTGGTGTGTT CACCAAGATC ATCGAGAAGA

Vesicatori CCCCGCTGTC GCTGGGTATC GAGACCATGG GCGGCGTGTT CACCAAGATC ATCGAAAAGA

Citri CCCCGCTGTC GCTGGGCATC GAGACCATGG GCGGCGTGTT CACCAAGATC ATCGAAAAGA

Oryzae CCCCGCTGTC GCTGGGTATC GAAACCATGG GCGGCGTGTT CACCAAGATC ATCGAAAAGA

Campestris CCCCGCTGTC GCTGGGTATC GAAACCATGG GCGGCGTGTT CACCAAGATC ATCGAAAAGA

Xyl9a5C CCCCGTTATC CCTTGGTATT GAAACGATGG GTGGGGTGTT TACAAAGATT ATCGAGAAGA

XylTemecul CCCCGTTATC CCTTGGTATT GAAACGATGG GTGGTGTGTT TACCAAGATT ATCGAGAAGA

Burkholder CGCCGCTGTC GCTCGGCATC GAGACGCTCG GCGGCGTGAT GACGAAGATG ATCAACAAGA

Ralstonia CGCCGCTGTC GCTGGGCATC GAGACGCTGG GCGGCGTGAT GACCAAGATG ATCGGCAAGA

....|....| ....|....| ....|....| ....|....| ....|....| ....|....|

5345 5355 5365 5375 5385 5395

X.albiline ACACCACCAT CCCGACCAAG GCCGCGCAGG TGTTCTCCAC TGCCGAGGAC AATCAGTCGG

StenoK279a ACACCACCAT CCCGACCAAG GCCTCGCAGG TGTTCTCCAC CGCCGAGGAC AACCAGTCGG

StenoR551 ACACCACCAT CCCGACCAAG GCCTCGCAGG TGTTCTCCAC CGCCGAGGAC AACCAGTCGG

Vesicatori ACACCACCAT TCCGACCAAG GCCTCGCAGA CCTTCTCCAC CGCCGAAGAC AACCAGTCGG

Citri ACACCACGAT CCCGACCAAG GCCTCGCAGA CCTTCTCCAC CGCCGAAGAC AACCAGTCTG

Oryzae ACACCACCAT CCCGACCAAG GCTTCGCAGA CCTTCTCCAC CGCCGAAGAC AACCAGTCGG

Campestris ACACCACCAT CCCGACCAAG GCCTCGCAGA CCTTCTCCAC TGCCGAAGAC AACCAGTCGG

Xyl9a5C ACACGACCAT TCCGACCAAA GCGTCTCAGG TGTTTTCTAC TGCCGAGGAT GGTCAGTCCG

XylTemecul ACACGACCAT TCCGACCAAA GCGTCCCAGG TGTTTTCTAC TGCCGAGGAT GGTCAGTCCG

Burkholder ACACGACGAT CCCGACGAAG CACGCTCAGG TGTATTCGAC GGCGGACGAC AACCAGGGCG

Ralstonia ACACGACCAT CCCGACCAAG TTCTCGCAGA CCTTCTCGAC CGCCGACGAC AACCAGCCGG

....|....| ....|....| ....|....| ....|....| ....|....| ....|....|

5405 5415 5425 5435 5445 5455

X.albiline CAGTGACCGT GCATGTGCTG CAGGGCGAGC GCGAACAGGC TCGCTATAAC AAGTCTTTGG

StenoK279a CCGTGACCGT GCACGTGCTG CAGGGTGAGC GCGAACAGGC CCGCTTCAAC AAGTCGCTGG

StenoR551 CCGTGACCGT GCACGTGCTG CAGGGTGAGC GCGAGCAGGC CCGCTTCAAC AAGTCGCTGG

Vesicatori CCGTGACCGT GCATGTGTTG CAGGGTGAGC GCGAGCAGGC CCGCTTCAAC AAGTCGCTGG

Citri CCGTGACCGT GCACGTGTTG CAGGGTGAGC GCGAGCAGGC CCGCTTCAAC AAGTCGCTGG

Oryzae CCGTGACCGT GCACGTGTTG CAGGGTGAGC GCGAGCAGGC CCGCTTCAAC AAGTCGCTGG

Campestris CCGTGACCGT GCACGTGCTG CAGGGTGAGC GCGAGCAGGC CCGCTTCAAC AAGTCGCTGG

Xyl9a5C CAGTGACTGT CCATGTGTTG CAAGGTGAAC GTGAGCAGGC GCGTTTCAAT AAATCGCTTG

XylTemecul CAGTGACTGT CCATGTGTTG CAAGGTGAAC GTGAGCAGGC GCGTTTCAAT AAATCGCTTG

Burkholder CCGTGACGAT CAAGGTGTTC CAGGGCGAAC GCGAGATGGC GGCGGGCAAC AAGCTGCTCG

Ralstonia CCGTGACGAT CAAGGTCTAC CAGGGCGAGC GCGAGATGGC CTCCGGCAAC AAGATGCTGG

....|....| ....|....| ....|....| ....|....| ....|....| ....|....|

5465 5475 5485 5495 5505 5515

X.albiline CCAAGTTTGA TCTGTCCGGC ATTGAGCCGG CGCCGCGTGG CCTGCCGCAG GTGGAGGTGT

StenoK279a CCAAGTTCGA CCTGTCCGGC ATCGAGCCGG CCCCGCGTGG CCTGCCGCAG GTGGAAGTGT

StenoR551 CCAAGTTCGA CCTGTCCGGC ATCGAGCCGG CCCCGCGCGG CCTGCCGCAG GTGGAAGTGT

Vesicatori CCAAGTTCGA CCTGTCCGGC ATCGAGCCGG CGCCGCGCGG CATGCCGCAG GTGGAAGTGT

Citri CCAAGTTCGA CCTGTCCGGC ATCGAGCCGG CGCCGCGCGG CATGCCGCAG GTGGAAGTGT

Oryzae CCAAGTTCGA CCTGTCCGGC ATCGAGCCGG CCCCGCGTGG CATGCCGCAG GTGGAAGTGT

Campestris CCAAGTTCGA CCTCTCCGGC ATCGAGCCGG CGCCGCGTGG CATGCCGCAG GTGGAAGTGT

Xyl9a5C CTAAATTCGA TTTGGCGGGT ATCGAACCTG CGCCGCGTGG CCAGCCGCAG ATTGAGGTGT

XylTemecul CTAAATTTGA TTTGGCGGGT ATCGAACCTG CGCCGCGTGG CCAGCCGCAG ATTGAGGTGT

Burkholder GCGAGTTCAA CCTCGAGGGC ATCCCGCCCG CGCCGCGCGG CGTGCCGCAG ATCGAAGTGA

Ralstonia GCGAGTTCAA CCTCGAGGGC ATTCCGCCGG CACCGCGCGG CACGCCGCAG ATCGAGGTGT

....|....| ....|....| ....|....| ....|....| ....|....| ....|....|

5525 5535 5545 5555 5565 5575

X.albiline CCTTCGACAT CGACGCCAAC GGCATCTTGC ACGTGTCGGC CAAGGACAAG AAGACCAACA

StenoK279a CCTTCGACAT CGACGCCAAC GGCATCCTGC ACGTGTCGGC CAAGGACAAG AAGACCAACA

StenoR551 CCTTCGACAT CGACGCCAAC GGCATCCTGC ACGTGTCGGC CAAGGACAAG AAGACCAACA

Vesicatori CCTTCGACAT CGACGCCAAC GGCATCCTGC ACGTGTCGGC CAAGGACAAG AAGACCAACA

Citri CCTTCGACAT CGACGCCAAC GGCATCCTGC ACGTGTCGGC CAAGGACAAG AAGACCAACA

Oryzae CCTTCGACAT CGACGCCAAC GGCATCCTGC ACGTGTCGGC CAAGGACAAG AAGACCAACA

Campestris CCTTCGACAT CGACGCCAAC GGCATCCTGC ACGTGTCGGC CAAGGACAAG AAGACCAACA

Xyl9a5C CTTTTGATAT CGATGCCAAC GGTATTTTGC ATGTCTCTGC CAAGGACAAG AAAACCAATA

XylTemecul CTTTTGATAT TGATGCCAAC GGTATTTTGC ATGTCTCTGC CAAGGACAAG AAAACCAATA

Burkholder CCTTCGACAT CGACGCGAAC GGCATCCTGC ACGTCGGCGC GAAGGACAAG GCGACCGGCA

Ralstonia CGTTCGACAT CGACGCCAAC GGCATCCTGC ACGTCGGTGC CAAGGACAAG GCCACCGGCA

....|....| ....|....| ....|....| ....|....| ....|....| ....|....|

5585 5595 5605 5615 5625 5635

X.albiline AGGAACAGAA GGTCGAGATC AAGGCCGGGT CGGGTCTGTC GGACGCGGAA ATCCAGCGCA

StenoK279a AGGAACAGAA GGTCGAGATC AAGGCCGGTT CGGGCCTGTC CGAGGAAGAG ATCGCGCGCA

StenoR551 AGGAACAGAA GGTCGAGATC AAGGCCGGTT CGGGCTTGTC CGAGGAAGAG ATCGCACGCA

Vesicatori AGGAACAGAA GGTCGAGATC AAGGCCGGTT CGGGTCTGTC GGACGAAGAG ATCCAGCGCA

Citri AGGAACAGAA GGTCGAGATC AAGGCCGGTT CGGGTTTGTC GGATGAAGAG ATCCAGCGCA

Oryzae AGGAACAGAA GGTCGAGATC AAGGCCGGTT CGGGTCTGTC GGATGAAGAG ATCCAGCGTA

Campestris AGGAACAGAA GGTCGAGATC AAGGCCGGTT CGGGCCTGTC GGATGAAGAG ATCCAGCGCA

Xyl9a5C AGGAGCAGAA GGTCGAGGTT AAAGCTGGTT CTGGACTTTC GGATAGTGAG ATTCAACAGA

XylTemecul AGGAGCAGAA GGTCGAGGTT AAAGCTGGTT CTGGACTTTC GGATAGTGAG ATTCAACAGA

Burkholder AGGAAAACAA GATCACGATC AAGGCGAACT CGGGCCTGTC CGAAGCCGAG ATCGAGAAGA

Ralstonia AGGAAAACAA GATCACCATC AAGGCGAGCT CGGGCCTGAG CGAGGCCGAG ATCGAGCGTA

....|....| ....|....| ....|....| ....|....| ....|....| ....|....|

5645 5655 5665 5675 5685 5695

X.albiline TGGTCGCCGA TGCCGAAGCC AATCGCGAGG AAGACAAGAA GTTCCACGAG TTGGTGCAGG

StenoK279a TGGTCGCCGA CGCGGAAGCC AACCGCGAAG AAGACAAGAA GTTCCAGGAA CTGGTGCAGG

StenoR551 TGGTCGCCGA CGCGGAAGCC AACCGCGAAG AAGACAAGAA GTTCCAGGAA CTGGTGCAGG

Vesicatori TGGTCGCCGA CGCGGAAGCC AACCGCGAAG AAGACAAAAA GTTCCAGGAG CTGGTGCAGG

Citri TGGTCGCCGA CGCGGAAGCC AACCGCGAAG AAGACAAGAA GTTCCAGGAG CTGGTGCAGG

Oryzae TGGTCGCCGA CGCGGAAGCC AACCGCGAAG AAGACAAGAA GTTCCATGAG CTGGTGCAGG

Campestris TGGTCGCCGA CGCGGAAGCC AACCGCGAAG AAGACAAGAA GTTCCAGGAG CTGGTGCAGA

Xyl9a5C TGGTTGCCGA TGCTGAGGCA CATCGTGAGG AAGACAAAAA GTTCCAAGAG TTGGTGCAGG

XylTemecul TGGTTGCCGA TGCTGAGGCA CATCGTGAGG AAGACAAAAA GTTCCAAGAG TTGGTGCAGG

Burkholder TGGTGAAGGA CGCGGAAGCG AACGCGGCGG AAGACCACAA GCTGCGTGAG CTCGCCGAAT

Ralstonia TGGTGAAGGA CGCCGAGGCC AACGCCGAGG AAGACAAGAA GCTGCGCGAA CTGGTCGACT

....|....| ....|....| ....|....| ....|....| ....|....| ....|....|

5705 5715 5725 5735 5745 5755

X.albiline CGCGCAACCA GGCCGATGGC TTGATCCACT CCACGCGCAG CGCGATCGCC GAGCACGGCA

StenoK279a CCCGCAACCA GGCCGACGCC CTGATCCACG GCACCCGCAG CGCCATCACC GAGCACGGCA

StenoR551 CCCGCAACCA GGCCGACGCC CTGATCCACG GCACCCGCAG CGCCATCACC GAGCACGGCA

Vesicatori CCCGCAACCA GGCCGATGGT CTGATCCACG CCACCCGTAC CGCGATCACC GAGCATGGCA

Citri CCCGCAACCA GGCCGATGGT CTGATCCACG CTACCCGTAC CGCAATCACC GAGCATGGCA

Oryzae CACGCAACCA GGCCGATGGC CTGATCCATG CCACCCGCAC CGCGATCACC GAGCATGGCA

Campestris CCCGCAACCA GGCCGATGGC TTGATCCACG CCACCCGCAC CGCGATCACC GAGCATGGCA

Xyl9a5C CAAGGAACCA TGCTGACGGT TTGATCCATT CAACCCGTTC GGCCATTAAG GAGCATGGTA

XylTemecul CAAGGAACCA TGCTGACGGT TTGATCCATT CAACTCGTTC GGCCATTAAG GAGCATGGTA

Burkholder CCCGCAACCA GGGCGACGCG CTCGTGCACA GCACGAAGAA GGCGCTCACC GAGTACGGCG

Ralstonia CCCGCAACCA GGGCGAAGCG CTGGTGCACT CGACCAGGAA GGCGCTGGGC GAATACGGCG

....|....| ....|....| ....|....| ....|....| ....|....| ....|....|

5765 5775 5785 5795 5805 5815

X.albiline GCAAGGTTGG CGGCGACGTG ATTGGCAAGG TCGAAGCCGC ACTGGCCGAT CTTGAAACCG

StenoK279a GCAAGGTCGG CGGCGATGTC ATCGGCAAGG TCGAGGCGGC CCTGGCCGAC CTGGAAACCG

StenoR551 GCAAGGTTGG CGGCGATGTC ATCGGCAAGG TGGAAGCGGC CCTGGCCGAC CTGGAAACCG

Vesicatori GCAAGGTCGG TGGCGATGTG ATCGGCAAGG TGGAAGCGGC GCTGTCGGAT CTGGAAACCG

Citri GCAAGGTCGG TGGCGATGTG ATCGGCAAGG TGGAAGCGGC TCTGTCGGAT CTGGAAACCG

Oryzae GCAAGGTCGG TGGCGATGTG ATCGGCAAGG TGGAAGCGGC GCTGTCGGAT CTGGAGACCG

Campestris GCAAGGTGGG TGGCGATGTG ATCGGCAAGG TGGAAGCGGC CCTGGCCGAC CTGGAAACCG

Xyl9a5C GTAAGGTTGG GGGTGAGGTG ATTGGGCGTG TCGAAGCCTC CCTTGCAGAG CTTGAGGCTG

XylTemecul GTAAGGTTGG GGGTGAGCTG ATTGGGCGTG TCGAAGCCTC CCTTGCAGAG CTTGAGGCTG

Burkholder ACAAGCTGGA GGCGGGCGAG AAGGAGAAGA TCGAAGCGGC GCTCAAGGAG CTCGAGGACG

Ralstonia ACAAGCTGGA AGCGGGCGAG AAGGACAAGA TCGAAGCCGC GATCAAGGAC CTTGAAGACG

....|....| ....|....| ....|....| ....|....| ....|....| ....|....|

5825 5835 5845 5855 5865 5875

X.albiline CGATGAAGGG C------GAG GACAAGAGCC AGATTGAGGC CAAGAGCAAA ACGTTGG---

StenoK279a CGATGAAGGG T------GAC GACAAGGCAC AGATCGAAGC CAAGTCGAAG GCACTGG---

StenoR551 CGATGAAGGG T------GAC GACAAGGCGC AGATCGAAGC CAAGTCGAAG GTGCTGG---

Vesicatori CCATGAAGGG C------GAC GACAAGGCGC AGATCGAAGC ACGCACCAAG ACGCTGG---

Citri CCATGAAGGG C------GAC GACAAGGCGC AGATCGAAGC GCGCACCAAG ACGCTGG---

Oryzae CCATGAAGGG C------GAC GACAAGGCGC AGATCGAAGC ACGCACCAAG ACGTTGG---

Campestris CCATGAAGGG C------GAC GACAAGGCGC AGATCGAAGC GCGCAGCAAG ACCCTGG---

Xyl9a5C CCGTCAAAGG T------GAT GATAAAAACC AGATTGAGGC GAAATCTAAA ACTCTTG---

XylTemecul CCGTCAAAGG T------GAT GATAAAAACC AGATTGAGGC GAAATCCAAA ACTCTTG---

Burkholder TGCTGAAGAA CGCGTCGAGC GACAAGGCGG CGATCGACGC GAAGGTCGAA GCGGTTGCGA

Ralstonia TCCTCAAGGG C------AGC GACAAGGCCG CGATCGACGC CAAGGTCGAA GCGCTGGCGA

....|....| ....|....| ....|....| ....|....| ....|....| ....|....|

5885 5895 5905 5915 5925 5935

X.albiline ---------A GGAGGTCGGC CAGTCGTTGT ACGCGG---C TGCGGC---- GGCCGGC---

StenoK279a ---------A AGAAGCTGGC CAGTCGCTGT TCGCCG---C TGCTTC---- GGCCGACCA-

StenoR551 ---------A GGAAGCTGGC CAGTCGCTGT TCGCCG---C CGCTTC---- GGCCGAGCA-

Vesicatori ---------A AGAAGCCGGT CAGTCGTTGT ACGCAGCAGC TGCGGC---- GGCAGAGCA-

Citri ---------A AGAAGCCGGC CAGTCGTTGT ACGCAGCAGC TGCGGC---- AGCAGAGCA-

Oryzae ---------A AGAAGCCGGC CAGTCGCTGT ACGCCGCAGC TGCGGC---- AGCAGAGCA-

Campestris ---------A AGAAGCCGGC CAGTCGCTGT ACGCCGCGGC CGCCGC---- GGCAGAGCA-

Xyl9a5C ---------A GGAAGTTGCA CAGTCGTTGC ACATGG---C AGCAAC---- AGCAGAGC--

XylTemecul ---------A GGAAGTTGCA CAGTCGTTGC ATATGG---C AGCAAC---- AGCAGAGC--

Burkholder CGGCGTCGCA GAAGCTCGGC GAGAAGATGT ACGCCGACAT GCAGGC---- -GCAGC----

Ralstonia CCGCCTCGCA GAAGCTGGGC GAGAAGGTCT ACGCCGACAT GCAGGCCAAG GGCGGCGCGG

....|....| ....|....| ....|....| ....|....| ....|....| ....|....|

5945 5955 5965 5975 5985 5995

X.albiline GAGCAGC--C GCCGGCCGCC AGTGCGGGTG GCGCGCATGC GTCCTCGTCG GCCGATGACG

StenoK279a GGGCGGT--- GCC----CCG GGTGCCGACG CCGGCAATGC CGGCAAGGCG CAGGACGACG

StenoR551 GGGCGGT--- GCCGCC-CCG GGCGCCGACG CCGGCAATGC GGGCAAGGCG CAGGATGACG

Vesicatori GGGCGGTAAC GCCGAT-GCG GCCAGCGGCA ACGCCCAGGC CTCCAAGGCC GCCGACGACG

Citri GGGCGGCAAC GCCGAT-GCG GCCAGCGGCA ACGCCCAGGC CTCCAAGGCC GCCGATGACG

Oryzae GGGCGGCAGC GCCGAT-GCC GCCAGCGGTA ACGCGCAGGC GTCCAAGGCC GCCGATGACG

Campestris GGGCGGCAAC GCCGAT-GCG GCCAGCGGCA ATGCGCAGGC CTCCAAGGCC GCCGATGACG

Xyl9a5C -AGCAATCAG GAAGCA--CG GGGGCTGGTG CAGGCTCTTC AGCCAAGGT- --TGACGATG

XylTemecul -AGCAGTCAG GAAGCA--CG GGGGCTGGTG CAGGCGCTTC AGCCAAGGT- --TGACGATG

Burkholder AGGCAGGCTC CGCGGGCGTG GCCGGGGCGG CAACCGAAGG TGCGTCGGCG CAGGGCGGCG

Ralstonia AAGCCGGCGC CGAGCAGGCT GCCGCCGGTG CCCACGCAGG CGCACAGGCC GGACACGGCG

....|....| ....|....| ....|....| ....|....| ....|....| ....|....|

6005 6015 6025 6035 6045 6055

X.albiline TG-------- -GTCGATGCC GAGTTTACCG AAGTCAAGGA CGGCAAG--- AAG---TAAA

StenoK279a TG-------- -GTCGACGCC GAGTTCACCG AAGTCAAGGA CGACAAG--- AAGTCCTGAA

StenoR551 TG-------- -GTGGATGCC GAGTTCACCG AAGTCAAGGA CGACAAG--- AAGTCCTGAA

Vesicatori TG-------- -GTGGACGCC GAGTTCACCG AGGTCAAGGA CGACAAG--- AAG---TAAA

Citri TG-------- -GTGGACGCC GAGTTCACCG AGGTCAAGGA CGACAAG--- AAG---TAAA

Oryzae TG-------- -GTGGACGCC GAGTTCACCG AAGTCAAGGA CGACAAG--- AAG---TAAA

Campestris TG-------- -GTGGACGCC GAGTTCACCG AGGTCAAGGA CGATAAG--- AAGGCGTGAA

Xyl9a5C TG-------- -GTTGATGCT GAATTCACAG AAGTCAAGGG CGACAAA--- AAA---TAAA

XylTemecul TG-------- -GTTGATGCT GAATTCACAG AAGTCAAGGC CGACAAA--- AAA---TAAA

Burkholder CGCAGCCGGC CGACGACGTC GTCGACGCCG ACTTCAAGGA AGTGAAG--- AAGGACTGAA

Ralstonia CGCCGCAGGA CGACAACGTC GTGGACGCCG AGTTCAAGGA AGTGAACGAC AAGAAGTAAA

....|....| ....|....| ....|....| ....|....| ....|....| ....|....|

6065 6075 6085 6095 6105 6115

X.albiline TGGCCAGCTA TGGCATGAAC GATGTCAAGA ACGGGATGAA GATCCTGGTC AACAGCGAAC

StenoK279a TGGCCAGCTA CGGCATGAAC GACGTCAAGA ACGGGATGAA GATCCTGGTC AACAACCAAC

StenoR551 TGGCCACTGC GGGCATGAAC GATGTCAAGA ACGGGATGAA GATCCTGGTC AACAATGAAC

Vesicatori TGGCCACTGT TGGCATGAAC GACGTCAAGA ACGGCATGAA GATCCTGGTC AACAACGAGC

Citri TGGCCACTGT TGGCATGAAC GACGTCAAGA ACGGCATGAA GATCCTGGTC AACAACGAGC

Oryzae TGGCCACTGT TGGCATGAAC GACGTCAAGA ACGGCATGAA GATCCTGGTC AACAACGAGC

Campestris TGGCCACTGT TGGCATGAAC GACGTCAAGA ACGGCATGAA GATCCTGGTC AACAACGAAC

Xyl9a5C TGGCCAGTTA CGGCATGAAC GATGTAAAAA ACGGCATGAA AATTCTGGTC AACGCCGAAC

XylTemecul TGGCCAGTTA CGGCATGAAC GATGTAAAAA ACGGCATGAA AATTCTGGTC AATGCCGAAC

Burkholder TG------AA AACCGCACAG GAACTCCGCG TAGGCAACGT CGTGATGATC GGCAACGACG

Ralstonia TGGCCTTGAA AATCGCGCAA GAACTCCGCG CTGGTAACGT TTTCATGATC GGCAACGACC

....|....| ....|....| ....|....| ....|....| ....|....| ....|....|

6125 6135 6145 6155 6165 6175

X.albiline CGGCGATCAT CACCGATACC GAATACGTGA AGCCGGGCAA GGGCCAGGCC TTCACCCGGG

StenoK279a CGGCCGTCAT CATCGACACC GAATACGTCA AGCCGGGCAA GGGCCAGGCC TTCACCCGCG

StenoR551 CGGCCGTCAT CTCCGAGACC GAGTTCATCA AGCCGGGCAA GGGCCAGGCC TTCACCCGCG

Vesicatori CGGCGGTCAT CACCGAGACC GAATACGTCA AGCCGGGCAA GGGCCAGGCC TTCACCCGCA

Citri CGGCGGTCAT CACCGAGACC GAATACGTCA AGCCGGGCAA GGGCCAGGCC TTCACCCGCA

Oryzae CGGCGGTCAT CACCGAGACC GAATACGTCA AGCCGGGCAA GGGCCAGGCC TTCACCCGCA

Campestris CGGCGGTCAT CACCGAGACC GAATACGTCA AGCCGGGCAA GGGCCAGGCC TTTACCCGCA

Xyl9a5C CCGCAGTCAT TACGGACACT GAATATGTCA AGCCTGGTAA GGGCCAAGCC TTTACCCGCG

XylTemecul CCGCAGTCAT TACGGACACT GAATATGTCA AGCCTGGTAA GGGCCAAGCC TTTACCCGCG

Burkholder CATGGGTCGT CTCGAAGACC GAATACAACA AGTCGGGCCG CAACGCCGCC GTCGTCAAGA

Ralstonia CGATGGTCGT GCTCAAGACC GAATACAGCC GCTCCGGCCG CAACGCCGCT GTCGTCAAGA

....|....| ....|....| ....|....| ....|....| ....|....| ....|....|

6185 6195 6205 6215 6225 6235

X.albiline TCAAATATCG TTTCATCAAA TCCGGGCGCG TGGTCGAAAT GACCATGAAG GCCACCGACA

StenoK279a TGAAGTACCG CCTGATCAAG GACGGCCGTA CCCAGGAAGT GACCATGAAG TCGACCGACT

StenoR551 TGCGCTATCG CTTCATCAAG TCGGGCCGCA CGGTCGAAAT GACCATGAAG GCGACCGATG

Vesicatori TGAAGTACCG CTTCATCAAG TCCGGTCGCG TGGTCGAAAT GACCATGAAG GCGACCGACG

Citri TGAAGTACCG CTTCATCAAG TCCGGGCGCG TGGTCGAAAT GACCATGAAG GCGACCGACG

Oryzae TGAAGTACCG CTTCATCAAG TCCGGGCGCG TGGTCGAAAT GACCATGAAG GCGACCGACG

Campestris TGAAGTACCG CTTCATCAAG TCCGGCCGCG TGGTGGAAAT GACCATGAAG GCCACCGATG

Xyl9a5C TCAAATACCG TTTGATCAAA TCCGGCCGGG TGCAGGAAGT CACCATGAAA TCCACCGATA

XylTemecul TCAAATACCG TTTGATCAAA TCTGGCCGGG TGCAGGAAGT CACCATGAAA TCCACCGATA

Burkholder TGAAGCTGAA GAACCTGCTG AATGGCGGAG GGCAGGAATC GGTGTACAAG GCCGACGACA

Ralstonia TGAAGTACAA GAACCTGCTG ACCGGCGCGC CGTCGGAATC GGTGTTCAAG GCCGACGACA

....|....| ....|....| ....|....| ....|....| ....|....| ....|....|

6245 6255 6265 6275 6285 6295

X.albiline GCGTGGAAGC GGCCGATGTG GTCGATACCA ACATGCAGTA TCTGTATACC GATGGCGAAT

StenoK279a CGCTGGATGC AGCCGACGTC GTCGATACCG ACATGAACTT CATGTACAGC GACGGCGAGT

StenoR551 ACGTGGAAGT GGCCGATGTG GTCGATACCA ACATGGATTA CATGTACAGC GACGGCGAGT

Vesicatori ATGTGGAAGT GGCCGACGTG GTCGATACCG ACATGCGCTA CCTCTACAGC GATGGCGAGT

Citri ATGTCGAAGT GGCCGACGTT GTCGATACCG ACATGCGCTA CCTCTACAGC GATGGCGAGT

Oryzae ACGTGGAAGT GGCCGATGTC GTCGATACCG ACATGCGCTA CCTGTACAGC GATGGCGAGT

Campestris ACGTGGAAGT GGCCGACGTG GTCGATACCG ACATGCGTTA CCTGTACACC GATGGCGAGT

Xyl9a5C CGCTTGAAGC AGCCGACGTG GTCGACACAG ATATGCAGTA TTTGTACAGC GATGGCGAAT

XylTemecul CGCTTGAAGC AGCCGACGTG GTCGATACAG ATATGCAGTA TTTGTACAGC GATGGCGAAT

Burkholder AGTTCGAAGT CGTCGTGCTC GACCGCAAGG AAGTGACGTA CTCGTACTTC GCCGATCCGA

Ralstonia AGATGGACCA GATCATCCTC GACAAGAAGG AGTGCACCTA CTCCTACTTC GCCGATCCGA

....|....| ....|....| ....|....| ....|....| ....|....| ....|....|

6305 6315 6325 6335 6345 6355

X.albiline ACTGGCACTT CATGCAGCAG GAGACCTTCG AGCAGGTGCA GGCCGACAAG GCCGGCGTCG

StenoK279a ACTGGCACTT CATGGACCCG GAATCCTTCG AGCAGGTCCA GGCCACCAAG GCCGGCATGG

StenoR551 ACTGGCACTT CATGGACCCG GAAACCTTCG AGCAGGTGCA GGCCGACAAG GCCGGCATGG

Vesicatori ACTGGCACTT CATGGACCCG GAAACCTTCG AGCAGGTGCA GACCGACAAG GCCGGCATGG

Citri ACTGGCACTT CATGGACCCG GACACCTTCG AGCAGGTGCA GACCGACAAG GCCGGCATGG

Oryzae ACTGGCACTT CATGGACCCG GAAACCTTCG AGCAGGTGCA GACCGACAAG GCCGGCATGG

Campestris ACTGGCACTT CATGGACCCG GAATCCTTCG AGCAGGTGCA GGCCGACAAG GCCGGCATGG

Xyl9a5C ACTGGCACTT CATGCAACAA GAAACCTTTG AACAAGTTCA AGCCGACAAA AACGGAATGG

XylTemecul ACTGGCACTT CATGCAACAA GAAACCTTTG AACAAGTTCA AGCCGACAAA AACGGAATGG

Burkholder TGTACGTGTT CATGGACGCC GA---CTACA ACCAGTACGA AGTCGAAGCG GAAATGATGG

Ralstonia TGTACGTGTT CATGGACACC GA---CTACA ACCAGTACGA AGTGGAAGCC GACAGCATGG

....|....| ....|....| ....|....| ....|....| ....|....| ....|....|

6365 6375 6385 6395 6405 6415

X.albiline GCGATGCCGC CAAATGGATC AAGGGGGAGG AAGACTGCGT GGTGACGTTG TGGAACGGTA

StenoK279a GCGGCGCCGA GAAGTGGCTG AAGGGCGAGG AGTCCTGCGT GGTCACCCTG TGGAACGGTG

StenoR551 GCGGCGCCGA GAAGTGGCTG AAGGGTGAAG AGTCCTGCAT CGTGACCCTG TTCAACGGTT

Vesicatori GCGGCGCCGA CAAGTGGCTC AAGGGCGAGG AAGACTGCAT CGTGACCTTG TGGAACGGCA

Citri GCGGCGCCGA CAAGTGGCTC AAGGGCGAGG AAGACTGCAT CGTGACCTTG TGGAACGGCA

Oryzae GCGGCGCCGA CAAGTGGCTG AAGGGCGAGG AAGACTGCAT CGTGACGTTG TGGAACGGTG

Campestris GCGGCGCGGA GAAGTGGCTC AAGGGCGAAG AAGACTGCAT CGTGACCTTG TGGAACGGCG

Xyl9a5C GTGGCGCCGA AAAATGGCTT AAAGGTGAAG AACAGTGTGT GGTCACACTC TGGAACGGTG

XylTemecul GTGGCGCCGA AAAATGGCTT AAAGGTGAAG AACAGTGTGT GGTCACACTC TGGAACGGTG

Burkholder GCGACGCGCT CAACTACCTC GAGGACGGCA TGGCATGCGA AGTCGTGTTC TACAACGAGA

Ralstonia GCGACGCGAT CCACTACCTG GAAGACGGCA TGGCCGCCGA AGTGACCTTC TACAACGAGA

....|....| ....|....| ....|....| ....|....| ....|....| ....|....|

6425 6435 6445 6455 6465 6475

X.albiline CCCCGATCCA GGTCACCCCG CCGAATTTCG TCGAGCTGAA GATCGTCCAG ACCGATCCGG

StenoK279a AGCCGATCTT CGTGCAGCCG CCGAACTTCG TCGAGCTGAA GATCACCGAA ACCGATCCGG

StenoR551 CGCCGATCTT CGTGCAGCCG CCGAACTTCG TCGAACTGAA GATCACCGAG ACCGATCCGG

Vesicatori CCCCGATCTG GGTGCAGCCG CCGAACTTCG TCGAGCTGAA GATCACCGAG ACCGATCCGG

Citri CGCCGATCTG GGTGCAGCCG CCGAACTTCG TCGAACTCAA GATCACCGAG ACCGACCCGG

Oryzae CGCCGATCTG GGTGCAGCCG CCGAATTTCG TCGAACTCAA GATCACCGAG ACCGACCCGG

Campestris CCCCGATCTG GGTGCAGCCG CCGAACTTCG TCGAGCTGAA GATCACCGAG ACCGACCCGG

Xyl9a5C TGCCGATCGG AGTGCAGCCG CCGAACTTCG TCGAGCTGAA AATCACCGAA ACCGATCCGG

XylTemecul TGCCGATCGG AGTGCAGCCA CCGAACTTCG TCGAACTGAA AATCACCGAA ACCGATCCGG

Burkholder AGGCGATCTC GGTCGAACTG CCGACGATCC TCGTTCGCGA GATCACGTAC ACGGAGCCGG

Ralstonia AGGCCATCTC GGTCGAACTG CCGACCACGC TGGTCCGCGA AATCGAATAC ACCGAGCCGG

....|....| ....|....| ....|....| ....|....| ....|....| ....|....|

6485 6495 6505 6515 6525 6535

X.albiline GCGTGCGTGG CGATACCTCT GGCGGCGGCG G---CAAGCC GGCCACGCTG GAGACCGGCG

StenoK279a GCGTCCGTGG CGACACCTCG GGCGGCGGCG G---CAAGCC GGCCACCCTG GAAACCGGCG

StenoR551 GCGTCCGTGG CGACACCTCG GGCGGCGGCG G---CAAGCC GGCCACCCTG GAAACCGGCG

Vesicatori GCGTGCGCGG CGACACCTCC GGCGGCGGCG G---CAAGCC GGCCACGCTG GAAACCGGCG

Citri GCGTGCGCGG CGATACCTCC GGCGGCGGCG G---CAAGCC GGCCACGCTG GAAACCGGCG

Oryzae GCGTGCGCGG CGATACCTCC GGTGGTGGCG G---CAAGCC GGCCACGCTG GAAACCGGCG

Campestris GCGTGCGTGG CGATACCTCT GGCGGCGGCG G---CAAGCC GGCCACCCTG GAAACCGGTG

Xyl9a5C GGTTACGCGG TGACACCTCT GGCGGCGGCG G---CAAGCC GGCCACCTTG GAAACCGGTG

XylTemecul GCTTACGCGG TGACACCTCT GGCGGCGGCG G---CAAGCC CGCTACCTTG GAAACCGGTG

Burkholder CCGTCAAGGG CGACACGTCG TCGGGCAAGG TGCTCAAGAA CGCGAAGCTC GCGACGGGCT

Ralstonia CCGTCAAGGG CGACACCTCG TCGGGCAAGG TGCTGAAGAT GGCCAAGATC AAGGGCGGCT

....|....| ....|....| ....|....| ....|....| ....|....| ....|....|

6545 6555 6565 6575 6585 6595

X.albiline CGGTGGTGCG GGTGCCCTTG TTCGTTGGCC AGGACGAAGT GATCAAGGTC GATACGCGCT

StenoK279a CCGTGGTCCG CGTGCCGCTG TTCGTGAACC AGGACGAAGT GATCCGCGTC GACACCCGTT

StenoR551 CAGTGGTCCG CGTGCCGCTG TTCGTCAACC AGGATGAAAT CATCAAGGTC GACACCCGCT

Vesicatori CGGTGGTGCG CGTGCCGCTG TTCGTCAACC AGGACGAGAT CATCAAGGTC GATACCCGTT

Citri CGGTGGTGCG CGTGCCGCTG TTCGTCAACC AGGACGAAAT CATCAAGGTC GACACCCGTT

Oryzae CAGTGGTGCG CGTGCCGCTG TTCGTCAACC AGGACGAAAT CATCAAGGTC GACACCCGTT

Campestris CGGTGGTGCG CGTGCCGCTG TTCGTCAACC AGGACGAAGT GATCAAGGTC GACACCCGCT

Xyl9a5C CCGTCGTGCG TGTCCCGTTA TTTGTGAATC AAGATGAAGT CATCAAAGTG GATACACGCT

XylTemecul CTGTCGTGCG TGTCCCGTTA TTTGTGAATC AAGATGAAGT CATCAAAGTG GATACCCGCT

Burkholder TCGAGCTGCA GGTGCCGCTC TTCTGCAACA CCGGCGACAA GATCGAAATC GACACGCGGA

Ralstonia TCGAGATCCA GGTCCCGCTG TTCTGCAGCA CCGGCGACAA GATCGAAATC GACACCCGTA

....|....| ....|....| ....|....| ....|....| ....|....| ....|....|

6605 6615 6625 6635 6645 6655

X.albiline CGGGCGAATA TTTCTCGCGC GTCAAGTAAA TGGCTGCCAA AGATATCCGT TTCGGCGAAG

StenoK279a CGGGCGAATA CTCCGCACGC GTCAAGTAAA TGGCTGCCAA GGATATTCGT TTCGGTGAAG

StenoR551 CGGGCGAGTA CTCCTCGCGC GTGAAGTAAA TGGCTGCCAA GGATATTCGT TTCGGTGAAG

Vesicatori CGGGCGAATA CTCGGCGCGC GTCAAGTAAA TGGCTGCTAA AGACATTCGT TTCGGTGAAG

Citri CGGGCGAATA CTCGGCGCGC GTCAAGTAAA TGGCTGCTAA AGACATTCGT TTCGGTGAAG

Oryzae CGGGCGAATA CTCGGCGCGC GTCAAGTAAA TGGCTGCTAA AGACATTCGT TTCGGTGAAG

Campestris CGGGCGAGTA CTCGGCGCGC GTCAAGTAAA TGGCTGCTAA AGACATTCGT TTCGGTGAAG

Xyl9a5C CTGGCGAGTA CGTCTCACGT GTCAAGTAAA TGGCTGCCAA AGAAATTATT TTCAGTGAAA

XylTemecul CTGGCGAGTA CGTCTCACGT GTCAAATAAA TGGCTGCCAA AGAAATTATT TTCAGTGAAA

Burkholder CGAACGAGTA CCGCAGCCGC GCG---TAAA TGGCAGCTAA AGACGTCGTA TTCGGCGATT

Ralstonia CGCACGAGTA CCGCAGCCGC GCCAACTGAA TGGCAGCTAA AGACGTAGTG TTCGGCGATG

....|....| ....|....| ....|....| ....|....| ....|....| ....|....|

6665 6675 6685 6695 6705 6715

X.albiline ACGCCCGTTC GCGCATGGTG CGCGGTGTCA ACATCCTCGC CAATGCCGTC AGTGCCACCC

StenoK279a ACGCCCGTTC GCGCATGGTG CGCGGCGTCA ACGTTCTCGC CAATGCCGTC AAGGCCACCC

StenoR551 ACGCCCGTTC GCGCATGGTG CGCGGCGTCA ACGTTCTCGC CAATGCCGTC AAGGCCACCC

Vesicatori ACGCACGTAC CCGCATGGTT CGTGGCGTCA ACGTGCTTGC CAATGCCGTG AAGGCCACCC

Citri ACGCACGTAC CCGCATGGTT CGTGGCGTCA ACGTGCTTGC CAATGCCGTG AAGGCAACCC

Oryzae ACGCACGTAC CCGCATGGTT CGTGGCGTCA ACGTGCTTGC CAATGCCGTG AAGGCCACCC

Campestris ATGCGCGTAC CCGTATGGTG CGTGGCGTCA ACGTTCTCGC CAATGCCGTG AAGGCAACCC

Xyl9a5C AAGCCCGTTC ACGTATGGTG CACGGGGTGA ACCTGCTTGC TAATGCAGTA AAAGCCACCT

XylTemecul AAGCCCGTTC ACGTATGGTG CACGGGGTGA ACCTGCTTGC TAATGCAGTG AAAGCCACCT

Burkholder CCGCACGCGC GAAGATGGTC GAAGGCGTGA ACATTCTCGC CAACGCTGTG AAGGTCACGC

Ralstonia CCGCACGCGC CAAGATGGTC GAGGGCGTCA ACATCCTCGC CAACGCCGTG AAGGTGACCC

....|....| ....|....| ....|....| ....|....| ....|....| ....|....|

6725 6735 6745 6755 6765 6775

X.albiline TTGGCCCGAA GGGCCGCAAC GTCGTGCTCG AGAAGAGCTT CGGTGCCCCG CACATCACCA

StenoK279a TGGGCCCGAA GGGCCGCAAC GTCGTGCTGG AAAAGAGCTT CGGCGCCCCG ACCATCACCA

StenoR551 TGGGCCCGAA GGGCCGCAAC GTCGTGCTGG AAAAGAGCTT CGGCGCTCCG ACCATCACCA

Vesicatori TGGGCCCGAA GGGCCGCAAC GTCGTGCTCG AGAAGAGCTT CGGCGCGCCG ACCATCACCA

Citri TGGGCCCGAA GGGCCGCAAC GTCGTGCTCG AGAAGAGCTT CGGCGCGCCG ACCATCACCA

Oryzae TGGGCCCGAA GGGCCGCAAC GTCGTGCTCG AAAAGAGCTT CGGCGCCCCG ACCATCACCA

Campestris TGGGCCCGAA GGGCCGCAAC GTCGTGCTCG AGAAGAGCTT CGGCGCGCCG ACCATCACCA

Xyl9a5C TAGGTCCCAA AGGCCGCCAC GTAGTGCTTG ATAAGAGCTT CGGCTCCCCA ATCATTACTA

XylTemecul TAGGTCCCAA AGGCCGCCAC GTAGTGCTTG ATAAGAGCTT CGGCTCCCCA ATCATTACTA

Burkholder TGGGTCCGAA GGGCCGCAAC GTGGTGCTCG AGCGCAGCTT CGGCGGCCCG ACGGTCACGA

Ralstonia TGGGCCCGAA GGGCCGCAAC GTGGTGCTGG AGCGCAGCTT CGGTGGCCCG ACCGTGACCA

....|....| ....|....| ....|....| ....|....| ....|....| ....|....|

6785 6795 6805 6815 6825 6835

X.albiline AGGACGGCGT CTCCGTCGCC AAGGAAATCG AACTGGCGGA TAAGTTCGAA AATATGGGCG

StenoK279a AGGACGGCGT CTCCGTCGCC AAGGAAATCG AACTGGCTGA CAAGTTCGAG AACATGGGCG

StenoR551 AGGACGGCGT CTCCGTCGCC AAGGAAATCG AACTGGCTGA CAAGTTCGAG AACATGGGCG

Vesicatori AGGACGGCGT TTCCGTCGCC AAGGAAATCG AACTGGCCGA CAAGTTCGAG AACATGGGCG

Citri AGGATGGCGT CTCCGTCGCC AAGGAAATCG AACTGGCTGA CAAGTTCGAG AACATGGGCG

Oryzae AGGACGGCGT CTCCGTCGCC AAGGAAATCG AACTGGCCGA CAAGTTCGAG AACATGGGCG

Campestris AGGACGGCGT CTCCGTCGCC AAGGAAATCG AACTGGCTGA CAAGTTCGAG AACATGGGCG

Xyl9a5C AAGACGGCGT CTCTGTCGCC AAAGAAATTG AGCTGGCCGA CAAGTTCGAA AACATGGGCG

XylTemecul AAGACGGCGT CTCTGTCGCC AAAGAGATTG AGTTGGCCGA CAAGTTTGAA AACATGGGCG

Burkholder AGGACGGTGT GTCGGTCGCG AAGGAAATCG AGCTGAAGGA CAAGCTCCAG AACATGGGCG

Ralstonia AGGACGGCGT GTCGGTCGCC AAGGAAATCG AGCTGAAGGA CAAGCTGCAG AACATGGGCG

....|....| ....|....| ....|....| ....|....| ....|....| ....|....|

6845 6855 6865 6875 6885 6895

X.albiline CACAGATGGT GAAGGAAGTC GCCTCCAAAA CGTCCGATAA CGCCGGCGAT GGCACCACCA

StenoK279a CGCAGATGGT GAAGGAAGTT GCTTCGCGCA CCAACGACGA TGCCGGCGAC GGCACCACCA

StenoR551 CGCAGATGGT GAAGGAAGTT GCTTCCCGCA CCAACGACGA CGCTGGCGAC GGCACCACCA

Vesicatori CGCAGATGGT CAAGGAAGTC GCGTCCAAGA CCAACGACAA CGCCGGCGAC GGCACCACCA

Citri CGCAGATGGT CAAGGAAGTC GCTTCCAAGA CCAACGACAA CGCCGGCGAC GGCACCACCA

Oryzae CGCAGATGGT CAAGGAAGTC GCTTCCAAGA CCAACGATAA CGCTGGCGAC GGCACCACCA

Campestris CGCAGATGGT CAAGGAAGTT GCTTCCAAGA CCAATGACAA CGCTGGCGAC GGCACCACCA

Xyl9a5C CACAAATGCT TAAAGAAGTC GCGTCCAAAA CAAATGACCA TGCAGGCGAC GGCACCACCA

XylTemecul CACAAATGCT TAAAGAAGTT GCGTCCAAAA CAAATGACCA TGCAGGCGAC GGCACCACCA

Burkholder CGCAGATGGT CAAGGAAGTC GCTTCCAAGA CCAGCGACAA CGCCGGCGAC GGCACGACGA

Ralstonia CGCAGATGGT CAAGGAAGTG GCTTCCAAGA CCAGCGACAA CGCCGGTGAC GGCACCACCA

....|....| ....|....| ....|....| ....|....| ....|....| ....|....|

6905 6915 6925 6935 6945 6955

X.albiline CCGCCACCGT GCTGGCGCAG GCGCTGATCC GCGAAGGCTC CAAGGCCGTG GCTGCCGGCA

StenoK279a CCGCCACCGT GCTGGCCCAG GCCCTGATCC GCGAAGGCGC CAAGGCCGTT GCCGCCGGCA

StenoR551 CCGCCACCGT GCTGGCCCAG GCCCTGATCC GCGAAGGTGC CAAGGCTGTT GCCGCCGGCA

Vesicatori CCGCCACCGT GCTGGCCCAG GCCCTGATCC GCGAAGGCGC CAAGGCCGTG GCCGCCGGCA

Citri CCGCCACCGT GCTGGCCCAG GCCCTGATCC GCGAAGGCGC CAAGGCTGTG GCCGCCGGCA

Oryzae CCGCCACCGT GCTGGCGCAG GCCCTGATCC GCGAAGGCGC CAAGGCTGTG GCCGCCGGCA

Campestris CCGCCACCGT GCTGGCCCAG GCCCTGATCC GCGAAGGCGC CAAGGCTGTG GCCGCCGGCA

Xyl9a5C CTGCCACGGT ACTAGCCCAG GCGTTGATCC GTGAAGGATG CAAAGCAGTG GCCGCTGGTA

XylTemecul CTGCAACGGT ACTAGCCCAG GCGTTGATCC GTGAAGGATG CAAAGCAGTG GCCGCTGGTA

Burkholder CGGCCACCGT CCTCGCGCAA TCGATCGTCC GCGAAGGCAT GAAGTACGTC GCATCGGGCA

Ralstonia CCGCCACGGT GCTGGCCCAG TCGATCGTGC GCGAAGGCAT GAAGTACGTG GCCGCCGGCA

....|....| ....|....| ....|....| ....|....| ....|....| ....|....|

6965 6975 6985 6995 7005 7015

X.albiline TGAACCCGAT GGACCTCAAG CGCGGCATCG ACAAGGCGGT TGCGGCCGCC GTGGTCGAGT

StenoK279a TGAACCCGAT GGACCTCAAG CGCGGTATCG ACAAGGCCGT TTCGGCCGCC GTCGCCGAGC

StenoR551 TGAACCCGAT GGACCTCAAG CGCGGTATCG ACAAGGCCGT CGTGGCCGCC GTCAACGAGC

Vesicatori TGAACCCGAT GGACCTCAAG CGTGGTATCG ACCAGGCCGT CAAGGCTGCG GTCGTCGAGC

Citri TGAACCCGAT GGACCTCAAG CGCGGTATCG ACCAGGCCGT CAAGGCTGCG GTCGTCGAGC

Oryzae TGAACCCGAT GGATCTGAAG CGCGGTATCG ACCAAGCCGT CAAGGCTGCG GTCGTCGAGC

Campestris TGAACCCGAT GGATCTGAAG CGCGGTATCG ACCAGGCCGT CAAGGCCGCC GTCATCGAGC

Xyl9a5C TGAATCCAAT GGATCTTAAG CGTGGTATCG ATAAAGCAGT CATTGCCGCG GTTACCGAAC

XylTemecul TGAATCCAAT GGATCTTAAG CGTGGTATCG ATAAAGCAGT CATTGCCGCG GTTACCGAAC

Burkholder TGAACCCGAT GGACCTGAAG CGCGGCATCG ACAAGGCAGT CGCCGCGGCA GTCGAAGAGC

Ralstonia TGAACCCGAT GGACCTGAAG CGCGGCATCG ACAAGGCCGT CGCCGCTGCC GTCGAAGAGC

....|....| ....|....| ....|....| ....|....| ....|....| ....|....|

7025 7035 7045 7055 7065 7075

X.albiline TGAAGAAGAT CAGCAAGCCC ACCGCCGACG ACAAGGCCAT CGCCCAGGTC GGCACCATCT

StenoK279a TGAAGAACAT CTCCAAGCCG ACCGCCGACG ACAAGGCGAT CGCCCAGGTC GGTACCATCT

StenoR551 TGAAGAGCAT CTCCAAGCCG ACCGCTGACG ACAAGGCGAT CGCCCAGGTC GGCACCATCT

Vesicatori TGAAGAACAT CTCCAAGCCC ACCACCGACG ACAAGGCGAT TGCCCAGGTC GGCACCATTT

Citri TGAAGAACAT CTCCAAGCCC ACCACCGACG ACAAGGCGAT CGCCCAGGTC GGCACCATCT

Oryzae TGAAGAACAT CTCCAAGCCC ACCACCGACG ACAAGGCGAT TGCCCAGGTC GGCACCATCT

Campestris TGAAGAACAT CTCCAAGCCC ACCACCGACG ACAAGGCGAT TGCCCAGGTC GGCACCATCT

Xyl9a5C TGAAGAAGAT CTCCAAGCCA ACCAGTGACG ATAAAGCCAT TGCCCAAGTC GCAACAATCT

XylTemecul TGAAGAAGAT CTCCAAGCCA ACCAGCGACG ATAAAGCCAT TGCCCAAGTC GCAACCATCT

Burkholder TGAAGAAGAT CAGCAAGCCG TGCACGACGA ACAAGGAAAT CGCGCAAGTC GGCGCGATCT

Ralstonia TGAAGAAGAT CAGCAAGCCG ACTACCACCA GCAAGGAAAT CGCCCAGGTT GGAGCCATCT

....|....| ....|....| ....|....| ....|....| ....|....| ....|....|

7085 7095 7105 7115 7125 7135

X.albiline CCGCCAACTC CGACGCGTCG ATCGGCGACA TCATCGCCGA TGCGATGAAG AAGGTCGGCA

StenoK279a CGGCCAACTC GGACGAGTCG ATCGGCCAGA TCATCGCTGA CGCGATGAAG GAAGTCGGCA

StenoR551 CGGCCAACTC GGACGAGTCG ATCGGCCAGA TCATCGCTGA CGCGATGAAG GAAGTCGGCA

Vesicatori CGGCCAACTC GGACGAGTCG ATCGGCAACA TCATTGCCGA AGCGATGAAG AAGGTCGGCA

Citri CGGCCAACTC GGACGAGTCG ATCGGCAACA TCATTGCCGA AGCGATGAAG AAGGTCGGCA

Oryzae CGGCCAACTC GGACGAGTCG ATCGGCAACA TCATTGCTGA AGCGATGAAG AAGGTCGGCA

Campestris CGGCCAACTC GGACGAATCG ATCGGCAACA TCATTGCCGA AGCGATGCAG AAGGTCGGCA

Xyl9a5C CTGCTAACTC AGACGAGTCT ATCGGCAACA TTATTGCCGA AGCAATGAAG AAGGTCGGTA

XylTemecul CTGCTAACTC AGACGAGTCT ATCGGCAACA TTATTGCCGA AGCAATGAAG AAGGTCGGTA

Burkholder CGGCGAACAG CGATTCGTCG ATCGGCGATC GCATCGCTGA AGCGATGGAC AAGGTCGGCA

Ralstonia CGGCCAACAG CGACGAGTCG ATCGGCGCGC GCATCGCTGA AGCGATGGAC AAGGTGGGCA

....|....| ....|....| ....|....| ....|....| ....|....| ....|....|

7145 7155 7165 7175 7185 7195

X.albiline AGAAGGGTGT GATCACGGTC GAGGAAGGCT CGGGCCTGGT CAACGAACTG GATGTGGTCG

StenoK279a AGGAAGGCGT CATCACCGTT GAAGAAGGCT CGGGCCTGGA CAACGAGCTG GACGTGGTCA

StenoR551 AGGAAGGCGT GATCACCGTT GAAGAGGGCT CGGGCCTGGA CAACGAGCTG GACGTGGTCA

Vesicatori AGGAAGGCGT GATCACCGTT GAAGAAGGCT CGGGCCTGGA AAACGAGCTG GACGTGGTCG

Citri AGGAAGGCGT GATCACCGTT GAAGAAGGCT CGGGCCTGGA AAACGAGCTG GACGTGGTCG

Oryzae AGGAAGGCGT GATCACCGTT GAAGAAGGCT CGGGCCTGGA AAACGAGCTG GACGTGGTCG

Campestris AGGAAGGCGT GATCACCGTT GAAGAAGGCT CGGGCCTGGA AAACGAGCTG GACGTGGTCG

Xyl9a5C AAGAAGGAGT GATTACTATT GAGGAAGGTA CGACTCTAGA AAACGAATTG GATGTCGTCG

XylTemecul AAGAAGGAGT GATTACCATT GAGGAAGGCA CAACTCTAGA AAACGAATTG GATGTCGTCG

Burkholder AGGAAGGCGT GATCACCGTC GAAGACGGCA AGTCGCTCGC CGACGAGCTC GACGTCGTCG

Ralstonia AGGAAGGCGT GATCACCGTG GAAGACGGCA AGTCGCTGGA AGACGAGCTG GACGTCGTGG

....|....| ....|....| ....|....| ....|....| ....|....| ....|....|

7205 7215 7225 7235 7245 7255

X.albiline AAGGCATGCA GTTCGACCGC GGCTACCTGT CGCCATACTT CATCAACAAC CAGCAGAGCC

StenoK279a AGGGCATGCA GTTCGACCGC GGCTACCTGT CGCCGTACTT CATCAACAAC CAGCAGTCGC

StenoR551 AGGGCATGCA GTTCGACCGC GGCTACCTGT CCCCGTACTT CATCAACAAC CAGCAGTCGC

Vesicatori AGGGCATGCA GTTCGACCGC GGCTACCTCT CCCCGTACTT CATCAACAAC CAGCAGAGCC

Citri AGGGCATGCA GTTCGACCGC GGCTATCTCT CCCCGTACTT CATCAACAAC CAGCAGAGCC

Oryzae AGGGCATGCA GTTCGATCGC GGCTACCTCT CCCCGTACTT CATCAACAAC CAGCAGAGCC

Campestris AGGGCATGCA GTTCGATCGC GGCTACCTCT CCCCGTACTT CATCAACAAC CAGCAGAGCC

Xyl9a5C AAGGGATGCA GTTTGACCGT GGTTACTCTT CGCCATATTT CATCAACAAC CAGCAGTCCC

XylTemecul AAGGGATGCA GTTTGACCGT GGTTACTCTT CGCCATATTT CATCAACAAC CAGCAGTCCC

Burkholder AAGGCATGCA GTTCGACCGC GGCTACCTGT CGCCGTACTT CATCAACAAC CCGGACAAGC

Ralstonia AAGGCATGCA GTTCGACCGC GGCTACCTGT CGCCGTACTT CATCAACAAC CCGGAAAAGC

....|....| ....|....| ....|....| ....|....| ....|....| ....|....|

7265 7275 7285 7295 7305 7315

X.albiline AGTCGGCCGA CTTGGACGAT CCGTTCATCC TGCTGCACGA CAAGAAGATC TCCAACGTGC

StenoK279a AGACCGCTGA CCTGGATGAC CCGTTCATCC TGCTGCACGA CAAGAAGATC TCCAACGTCC

StenoR551 AGACCGCTGA TCTGGATGAC CCGTTCATCC TGCTGCACGA CAAGAAGATC TCCAACGTCC

Vesicatori AGTCGGCCGA CCTGGACGAC CCGTTCATCC TGCTGCACGA CAAGAAGATC TCCAACGTGC

Citri AGTCGGCCGA TCTGGACGAC CCGTTCATCC TGCTGCACGA CAAGAAGATC TCCAACGTGC

Oryzae AGTCGGCCGA CCTGGACGAC CCGTTCATCC TGCTGCACGA CAAGAAGATC TCCAACGTGC

Campestris AGTCGGCCGA TCTGGACGAC CCGTTCATCC TGCTGCACGA CAAGAAGATC TCCAACGTGC

Xyl9a5C AAATTGTTGA GCTGGATAAT CCCTACATCC TCCTTCACGA CAAGAAAATT TCCAGTGTGC

XylTemecul AAATTGTTGA GCTGGATAAT CCCTACATCC TCCTTCACGA CAAGAAAATT TCCAGTGTGC

Burkholder AAGTCGCCGT CCTCGAGAAC CCGTTCGTGC TGCTGCACGA CAAGAAGGTG TCGAACATCC

Ralstonia AGGTTGTTCA GCTGGACAAC CCGTTCGTGC TGCTGTTCGA CAAGAAGATC AGCAACATCC

....|....| ....|....| ....|....| ....|....| ....|....| ....|....|

7325 7335 7345 7355 7365 7375

X.albiline GTGACCTGCT GCCGTTGCTG GAAGGCGTGG CCAAGGCCGG CAAGCCGCTG CTGATCGTCG

StenoK279a GTGACCTGCT GCCGGTGCTG GAAGGCGTCG CCAAGGCCGG CAAGCCGCTG CTGATCGTGG

StenoR551 GTGACCTGCT GCCGGTGCTG GAAGGCGTCG CCAAGGCCGG CAAGCCGCTG CTGATCGTCG

Vesicatori GTGACCTGCT GCCCGTGCTG GAAGGCGTGG CCAAGGCCGG CAAGCCGCTG CTGATCGTCG

Citri GCGACCTGCT GCCCGTGCTG GAAGGCGTGG CCAAGGCCGG CAAGCCGTTG CTGATCGTCG

Oryzae GTGACCTGCT GCCCGTGCTG GAAGGCGTGG CCAAGGCCGG CAAGCCGCTG CTGATCGTGG

Campestris GTGACCTGCT GCCCGTGCTG GAAGGCGTGG CCAAGGCCGG CAAGCCGCTG CTGATCGTCG

Xyl9a5C GCGATTTACT CACCGTGCTT GACGCCGTCG CCAAAGAAAG CAAGCCGTTG CTGATCGTCG

XylTemecul GCGATTTACT CACCGTGCTT GACGCCGTCG CCAAAGAAAG CAAGCCGTTG CTGATCGTCG

Burkholder GCGACCTGCT GCCGGTGCTC GAGCAAGTCG CGAAGGCTGG CCGTCCGCTG CTGATCATCG

Ralstonia GCGACCTGCT GCCGGTGCTG GAGCAAGTGG CCAAGGCCGG CCGTCCGCTG CTGATCGTCG

....|....| ....|....| ....|....| ....|....| ....|....| ....|....|

7385 7395 7405 7415 7425 7435

X.albiline CCGAGGAAGT GGAAGGCGAG GCGCTGGCGA CTCTGGTAGT CAACACCATT CGTGGCATCG

StenoK279a CCGAGGAAGT TGAAGGCGAA GCGCTGGCCA CCCTGGTCGT CAACACCATC CGTGGCATCG

StenoR551 CTGAAGAAGT CGAAGGCGAA GCGCTGGCGA CCCTGGTGGT CAACACCATC CGTGGCATCG

Vesicatori CCGAAGAAGT CGAAGGCGAA GCGCTGGCGA CCCTGGTGGT CAACACCATC CGCGGCATCG

Citri CCGAGGAAGT CGAAGGCGAA GCGCTGGCGA CCCTGGTGGT CAACACCATC CGCGGCATCG

Oryzae CGGAAGAAGT CGAAGGCGAA GCGTTGGCGA CCCTGGTGGT CAACACCATT CGTGGCATCG

Campestris CTGAAGAAGT CGAAGGCGAA GCCCTGGCGA CGCTGGTGGT CAACACCATC CGCGGCATCG

Xyl9a5C CTGAGGAAGT CGAAGGCGAA GCTTTGGCAA CTCTGGTCGT TAACAACATC CGCGGCATCA

XylTemecul CTGAGGAAGT CGAAGGCGAA GCTTTGGCAA CTCTGGTCGT TAACAACATC CGCGGCATCA

Burkholder CCGAAGACGT CGAAGGCGAA GCGCTCGCAA CGCTGGTCGT CAACAACATC CGCGGCATCC

Ralstonia CTGAAGATGT CGAAGGCGAA GCCCTGGCAA CGCTGGTGGT CAACAACATC CGTGGCATCC

....|....| ....|....| ....|....| ....|....| ....|....| ....|....|

7445 7455 7465 7475 7485 7495

X.albiline TCAAGGTCGT GGCGGTCAAG GCTCCTGGCT TCGGCGACCG CCGCAAGGCG ATGCTGGAAG

StenoK279a TCAAGGTCGT GGCCGTCAAG GCTCCGGGCT TCGGCGACCG TCGCAAGGCG ATGCTGGAAG

StenoR551 TCAAGGTCGT GGCCGTCAAG GCTCCGGGCT TCGGCGACCG TCGCAAGGCG ATGCTGGAAG

Vesicatori TCAAGGTCGT GGCCGTCAAG GCGCCGGGCT TCGGCGACCG TCGCAAGGCG ATGCTGGAAG

Citri TGAAGGTCGT GGCCGTCAAG GCGCCGGGCT TCGGCGACCG TCGCAAGGCG ATGCTGGAAG

Oryzae TCAAGGTCGT GGCCGTCAAG GCACCGGGCT TCGGCGACCG TCGCAAGGCG ATGCTGGAAG

Campestris TCAAGGTCGT GGCCGTCAAG GCACCGGGCT TCGGCGACCG TCGCAAGGCG ATGCTGGAAG

Xyl9a5C TCAAAGTCTG CGCAGTCAAA GCACCTGGCT TCGGTGATCG TCGCAAAGCC ATGCTGGAAG

XylTemecul TCAAAGTCTG CGCAGTCAAA GCACCTGGCT TCGGTGATCG TCGCAAAGCC ATGCTGGAAG

Burkholder TGAAGACCGT TGCGGTCAAG GCACCGGGCT TCGGCGATCG TCGCAAGGCG ATGCTGGAAG

Ralstonia TGAAGACCGC CGCCGTCAAG GCTCCGGGCT TCGGCGACCG CCGCAAGGCC ATGCTGGAAG

....|....| ....|....| ....|....| ....|....| ....|....| ....|....|

7505 7515 7525 7535 7545 7555

X.albiline ACATGGCGGT GCTGACTGGC GGCACCGTGA TCTCCGAGGA GGTGGGTCTG TCCCTCGAGA

StenoK279a ACATGGCCGT GCTGACCGGC GGCACCGTGA TCTCCGAAGA AGTCGGCCTG TCGCTGGAGA

StenoR551 ACATGGCCGT GCTGACCGGC GGCACCGTGA TCTCCGAAGA AGTGGGCCTG TCGCTGGAAA

Vesicatori ACATGGCCGT GCTGACCGGC GGTACCGTGA TCTCCGAGGA AGTGGGCCTG GCGCTTGAGA

Citri ACATGGCCGT GCTGACCGGC GGTACCGTGA TCTCCGAGGA AGTGGGTCTG GCGCTGGAGA

Oryzae ACATGGCCGT GCTGACCGGC GGCACCGTGA TCTCCGAGGA AGTGGGCCTG GCGCTGGAAA

Campestris ACATGGCCGT GCTGACCGGC GGCACCGTGA TCTCCGAGGA AGTGGGTCTG GCCCTGGAAA

Xyl9a5C ATATGGCTGT GCTGACAGGC GGCACCGTCA TCTCGGAAGA AGTAGGTCTG TCTCTGGAAA

XylTemecul ATATGGCTGT GCTGACAGGC GGCACCGTCA TCTCGGAAGA AGTAGGTCTG TCTCTGGAAA

Burkholder ACATCGCGAT CCTGACGGGC GGCCAGGTCA TCGCGGAAGA AACCGGCCTC ACGCTCGAGA

Ralstonia ACATCGCCAT CCTGACGGGC GGCCAGGTCA TCGCTGAAGA AGTCGGCCTG ACGCTGGAAA

....|....| ....|....| ....|....| ....|....| ....|....| ....|....|

7565 7575 7585 7595 7605 7615

X.albiline AGGCGACCCT GAAGGATCTG GGCCGCGCCA AGAAGGTACA GGTTTCCAAG GAGAACACCA

StenoK279a AGGCCACCAT CAAGGACCTG GGCCGCGCCA AGAAGGTGCA GGTCTCCAAG GAGAACACCA

StenoR551 AGGCCACCAT CAAGGATCTC GGCCGCGCCA AGAAGGTGCA GGTCTCCAAG GAAAACACCA

Vesicatori AGGCGACCAT CAAGGACCTG GGCCGCGCCA AGAAGGTGCA GGTCTCCAAG GAAAACACCA

Citri AGGCGACCAT CAAGGACCTG GGCCGCGCCA AGAAGGTGCA GGTCTCCAAG GAAAACACCA

Oryzae AGGCGACCAT CAAGGACCTG GGCCGCGCCA AGAAGGTGCA GGTTTCCAAA GAAAACACCA

Campestris AGGCCACGAT CAAGGATCTC GGCCGCGCGA AGAAGGTGCA GGTCTCCAAG GAGAACACCA

Xyl9a5C AGGCCACGAC CAGCCACCTT GGCAAGGCCA AGAAAGTACG CGTCTCTAAA GAAAACACCA

XylTemecul AGGCCACGAC CAGCCATCTT GGCAAGGCCA AGAAAGTACG CGTCTCTAAA GAAAACACCA

Burkholder AGGCAACGCT GGCAGAACTG GGCCAGGCGA AGCGCATCGA AGTGGGCAAG GAAAACACGA

Ralstonia AGGCGACCCT GAACGATCTG GGCCAAGCCA AGCGTGTGGA AATCGGCAAG GAAAACACCA

....|....| ....|....| ....|....| ....|....| ....|....| ....|....|

7625 7635 7645 7655 7665 7675

X.albiline CCATCATCGA TGGCGTCGGC GATACGGCCG CGATCGAGTC GCGCATCAAG CAGATCGAAT

StenoK279a CCATCATCGA CGGCGTCGGC GACAAGGCCA ACGTTGATGC ACGCGTGGCG CAGATCAAGA

StenoR551 CCATCATCGA TGGCGTGGGT GACAAGGCTG CGGTCGATTC GCGCGTTGCG CAGATCAAGA

Vesicatori CCATCATCGA CGGCGCCGGC GACACCGCGG CGATCGAATC GCGCGTGGGC CAGATCAAGA

Citri CGATCATCGA CGGCGCTGGC GATTCGGCCG CGATCGAGTC GCGCGTGGGC CAGATCAAGA

Oryzae CGATCATCGA CGGCGCTGGC GATTCGGCCG CGATCGAGTC GCGCGTCGGC CAGATCAAGA

Campestris CCATCATCGA CGGCGCCGGC GATTCGGCCA CGATCGAAGC CCGCGTGGGC CAGATCAAGA

Xyl9a5C CCATTATTGA TGGTATCGGT GACAACGATG CGATCAATGG TCGCGTCAAG CAAATCAAGA

XylTemecul CCATTATTGA TGGTATGGGT GACAACGATG CGATCAATGG TCGCGTCAAG CAAATCAAGA

Burkholder CGATCATCGA CGGCGCGGGC GAAGCCGTGA ACATCGAAGC GCGCGTCAAG CAAATCCGCA

Ralstonia CGATCATCGA TGGCGCCGGC GATGCCCGCA ACATCGAAGC GCGCGTCAAG CAAGTGCGCG

....|....| ....|....| ....|....| ....|....| ....|....| ....|....|

7685 7695 7705 7715 7725 7735

X.albiline TGCAGATTGC CGAGACCTCC TCCGACTACG ACAAGGAGAA GTTGCAGGAG CGCATGGCCA

StenoK279a CCCAGATCCA GGACACCTCC TCGGATTACG ACCGCGAGAA GCTGCAGGAA CGCGTGGCCA

StenoR551 CCCAGATCCA GGACACCTCC TCGGATTACG ACCGCGAGAA GCTGCAGGAA CGCGTGGCCA

Vesicatori CCCAGATCGA AGACACCTCG TCCGATTACG ACCGTGAGAA GCTGCAGGAG CGCGTGGCCA

Citri CCCAGATCGA AGACACCTCG TCCGATTACG ACCGCGAGAA GCTGCAGGAG CGCGTGGCCA

Oryzae CCCAGATCGA AGACACCTCG TCCGATTACG ACCGTGAAAA GCTGCAGGAA CGCGTGGCCA

Campestris CCCAGATCGA AGACACCTCG TCCGATTACG ACCGTGAGAA GCTGCAGGAA CGCGTGGCCA

Xyl9a5C CCCAGATCGA GGAAACCACC TCGGACTACG ACCGCGAAAA ACTGCAGGAA CGTGTAGCCA

XylTemecul CCCAGATCGA GGAAACCACC TCGGACTACG ACCGCGAAAA ACTGCAGGAA CGTGTAGCCA

Burkholder CGCAAATCGA AGAAGCGACG TCGGACTACG ACCGTGAAAA GCTGCAAGAG CGCGTGGCCA

Ralstonia CCCAGATCGA GGAAGCCACG TCGGACTACG ACCGTGAGAA GCTGCAAGAG CGCGTGGCCA

....|....| ....|....| ....|....| ....|....| ....|....| ....|....|

7745 7755 7765 7775 7785 7795

X.albiline AGCTGGCCGG CGGTGTGGCG GTGATCAAGG TCGGTGCTGC GACCGAAATC GAGATGAAGG

StenoK279a AGCTGGCCGG CGGTGTTGCC GTGATCAAGG TCGGCGCCTC GACCGAAATC GAAATGAAGG

StenoR551 AGCTGGCCGG CGGCGTTGCC GTGATCAAGG TCGGTGCCTC GACCGAAATC GAAATGAAGG

Vesicatori AGCTGGCCGG TGGCGTTGCA GTGATCAAGG TCGGCGCCTC GACCGAAATC GAAATGAAGG

Citri AGCTGGCCGG TGGCGTTGCG GTGATCAAGG TGGGCGCCTC GACCGAAATC GAGATGAAGG

Oryzae AGCTGGCCGG CGGCGTTGCA GTGATCAAGG TTGGCGCCTC GACCGAAATC GAAATGAAGG

Campestris AGCTGGCCGG TGGCGTTGCA GTGATCAAGG TCGGCGCGTC GACCGAAATC GAAATGAAGG

Xyl9a5C AGCTCGCCGG TGGTGTAGCC GTCATCAAGG TCGGTGCTGC AACCGAAGTG GAAATGAAGG

XylTemecul AGCTCGCCGG TGGTGTAGCC GTCATCAAGG TCGGTGCTGC AACCGAAGTG GAAATGAAGG

Burkholder AGCTGGCAGG CGGCGTGGCG GTGATCAAGG TCGGCGCTGC GACCGAAGTC GAAATGAAGG

Ralstonia AGCTGGCCGG CGGCGTGGCA GTGATCAAGG TTGGTGCGGC CACCGAAGTC GAAATGAAGG

....|....| ....|....| ....|....| ....|....| ....|....| ....|....|

7805 7815 7825 7835 7845 7855

X.albiline AAAAGAAGGC ACGCGTCGAA GATGCCCTGC ACGCCACCCG TGCCGCAGTG GAAGAAGGCG

StenoK279a AAAAGAAGGA TCGCGTCGAC GACGCCCTGC ACGCCACCCG TGCGGCCGTT GAAGAAGGCG

StenoR551 AAAAGAAGGA CCGCGTCGAC GACGCCCTGC ACGCCACCCG TGCAGCCGTT GAAGAAGGCG

Vesicatori AAAAGAAGGC ACGCGTCGAA GACGCCCTGC ACGCCACCCG TGCAGCCGTC GAAGAAGGCG

Citri AAAAGAAGGC ACGCGTCGAA GACGCCCTGC ACGCCACCCG TGCAGCCGTC GAAGAAGGCG

Oryzae AAAAGAAGGC ACGCGTCGAA GACGCCCTGC ACGCCACCCG TGCAGCCGTC GAAGAAGGCG

Campestris AAAAGAAGGC CCGCGTCGAA GACGCCCTGC ACGCCACCCG TGCAGCCGTC GAAGAAGGCG

Xyl9a5C AAAAGAAAGC ACGTGTTGAT GATGCTTTAC TTGCAACGCG TGCAGCCGTT GAAGAAGGAG

XylTemecul AAAAGAAAGC ACGTGTTGAT GATGCTTTAC TTGCAACCCG TGCAGCCGTT GAAGAAGGAG

Burkholder AAAAGAAGGC ACGTGTCGAG GACGCGCTGC ACGCCACCCG CGCTGCCGTT GAAGAAGGCA

Ralstonia AAAAGAAGGC CCGCGTGGAA GACGCCCTGC ACGCTACCCG CGCTGCCGTG GAAGAAGGCA

....|....| ....|....| ....|....| ....|....| ....|....| ....|....|

7865 7875 7885 7895 7905 7915

X.albiline TGGTCCCCGG CGGCGGCGTG GCGCTGGTGC GCGCGCTGAG CGCGATCGGT TCGCTCACGG

StenoK279a TGGTTCCGGG CGGCGGCGTT GCCCTGGTCC GCGCGATCAC CGCGCTGGCC GGCCTGAAGG

StenoR551 TGGTCCCGGG CGGCGGCGTT GCCCTGGTCC GTGCGGTCTC CGCGCTGGCT GGTCTGAAGG

Vesicatori TGGTCCCGGG CGGCGGCGTG GCCCTGGTGC GTGCGCTGGT GGCCGTGGGC GAGCTCAAGG

Citri TGGTCCCGGG CGGCGGCGTG GCCCTGGTGC GTGCGCTGGT GGCCGTGGGT GACCTGAAGG

Oryzae TGGTCCCGGG CGGCGGCGTG GCCCTGGTGC GTGCGCTGGT GGCCGTCGGT AACCTGACCG

Campestris TGGTCCCGGG CGGCGGTGTG GCCCTGGTGC GTGCGCTGGT GGCCGTCGGC AACCTGACCG

Xyl9a5C TGATTCCAGG CGGCGGCGTG GCCCTGATAC GTGCAATCAC TGCAATCAGC AATCTGAAGG

XylTemecul TGATTCCAGG TGGCGGCGTG GCCCTGATAC GTGCAATCAC GGCAATCAGC AATCTGAAGG

Burkholder TCGTCCCGGG CGGCGGCGTC GCGCTGATCC GCGCACGCAC CGCGATCGCG AGCCTGACCG

Ralstonia TCGTGGCTGG CGGCGGCGTT GCGCTGCTGC GTGCCCGTGC GCTGATCTCC GGCCTGAAGG

....|....| ....|....| ....|....| ....|....| ....|....| ....|....|

7925 7935 7945 7955 7965 7975

X.albiline GCGACAATGA AGATCAGACC CACGGCATCC AGATCGCGCT GCGCGCGATG GAAGCGCCGC

StenoK279a GCGCCAACGA AGACCAGAAC CACGGCATCC AGATCGCCCT GCGTGCGATG GAAGCCCCGC

StenoR551 GTGCCAACGA AGACCAGAAC CACGGCATCC AGATCGCCCT GCGCGCGATG GAAGCCCCGC

Vesicatori GCGCCAACGA AGACCAGACC CACGGCATCC AGATCGCCCT GCGCGCCATG GAAGCCCCGC

Citri GCGCCAACGA AGACCAGACC CACGGCATCC AGATCGCCCT GCGCGCCATG GAAGCCCCGC

Oryzae GTGCCAACGA AGACCAGACC CACGGCATCC AGATCGCTTT GCGCGCCATG GAAGCCCCGC

Campestris GTGCCAACGA AGACCAGACC CACGGCATCC AGATTGCCCT GCGCGCCATG GAAGCCCCGC

Xyl9a5C GTGCCAATGA AGACCAGACA CACGGCATTC AAATCGCATT GCGCGCTATG GAGGCACCAT

XylTemecul GTGCCAATGA AGACCAGACA CACGGCATTC AAATCGCATT GCGCGCTATG GAGGCACCAT

Burkholder GCGTGAACGC CGACCAGAAC GCCGGCATCA AGATCGTGCT GCGCGCGATG GAAGAGCCGC

Ralstonia GTGCCAACGC TGACCAGGAC GCCGGCATCA AGATCGTGCT GCGCGCCATG GAAGAGCCGC

....|....| ....|....| ....|....| ....|....| ....|....| ....|....|

7985 7995 8005 8015 8025 8035

X.albiline TGCGCGCGAT CGTCACCAAC GCTGGTGAAG AACCGTCGGT GATTCTCAAT AAGGTCAAGG

StenoK279a TGCGCGAGAT CGTTGCCAAC GCCGGTGACG AGCCGTCGGT CATCATCAAC AAGGTCAAGG

StenoR551 TGCGCGAGAT CGTCGCCAAC GCCGGCGAAG AGCCGTCGGT CATCGTCAAC AAGGTCAAGG

Vesicatori TGCGCGAGAT CGTGGCCAAT GCCGGCGAAG AGCCGTCCGT GATCCTGAAC AAGGTCAAGG

Citri TGCGCGAGAT CGTGGCCAAT GCCGGCGAAG AGCCCTCCGT GATCCTGAAC AAGGTCAAGG

Oryzae TGCGCGAAAT CGTCGCCAAT GCCGGCGAAG AGCCGTCCGT GATCCTGAAC AAGGTCAAGG

Campestris TGCGCGAAAT CGTGGCCAAC GCCGGCGAAG AGCCGTCCGT GATCCTGAAC AAGGTCAAGG

Xyl9a5C TACGCGAAAT TGTTGCCAAC GCCGGTGAAG AGCCATCGGT CATCTTAAAT AAGGTGAAAG

XylTemecul TACGCGAAAT TGTTGCCAAC GCCGGTGAAG AGCCATCGGT CATCTTAAAT AAGGTGAAAG

Burkholder TGCGCCAGAT CGTCACGAAC GGCGGCGAAG AAGCGAGCGT CGTGGTGGCG GCAGTTGCTG

Ralstonia TGCGCCAGAT CGTCACGAAC GCTGGCGACG AGGCTTCGGT GGTGGTGGCC AACGTCATCG

....|....| ....|....| ....|....| ....|....| ....|....| ....|....|

8045 8055 8065 8075 8085 8095

X.albiline AAGGCACCGG CAACTTCGGC TACGACGCTG CTAAAGGCGA GTTCGGCGAC ATGGTCGCGT

StenoK279a AAGGCACCGG CAGCTTCGGC TACAACGCCG CCACCGGCGA GTTCGGCGAC ATGCTGCAGT

StenoR551 AAGGCACCGG CAGCTTCGGC TACAACGCCG CCACCGGCGA GTTCGGTGAC ATGCTGCAGT

Vesicatori AAGGCAGCGG CAACTACGGC TACAACGCCG CCAACGGCGA GTTCGGCGAC ATGGTCGAAT

Citri AAGGCACCGG CAACTACGGC TACAACGCCG CCAACGGCGA GTTCGGCGAC ATGGTCGAAT

Oryzae AAGGCACCGG CAACTACGGC TACAACGCGG CCAACGGCGA GTTCGGCGAC ATGGTCGAAT

Campestris AAGGCACCGG CAACTACGGC TACAACGCAG CCAACGGCGA GTTCGGCGAC ATGGTCGAAT

Xyl9a5C AAGGCAAGGA CAATTTTGGC TACAACGCCG CTACTGGCGA ATTCGGCGAT ATGGTCAACC

XylTemecul AAGGCAAGGA CAATTTTGGC TACAACGCCG CTACTGGCGA ATTCGGCGAT ATGGTCAACC

Burkholder CGGGCAAGGG CAACTACGGC TACAACGCGG CGACGGGCGA GTACGTCGAC ATGGTCGAAG

Ralstonia CAGGCAAGGG CAACTACGGC TACAACGCCT CCACCGGCGA GTACGGTGAC CTGGTGGAAA

....|....| ....|....| ....|....| ....|....| ....|....| ....|....|

8105 8115 8125 8135 8145 8155

X.albiline TCGGTATCCT GGATCCGACC AAGGTGACCC GTTCGGCGCT GCAGAACGCT TCCTCGATCG

StenoK279a TCGGCATCCT GGACCCGACC AAGGTGACCC GTTCGGCGCT GCAGAACGCG GCCTCGATCG

StenoR551 TCGGCATCCT GGACCCGACC AAGGTGACCC GTTCGGCGCT GCAGAACGCA GCTTCGATCG

Vesicatori TCGGCATCCT GGACCCGACC AAGGTGACCC GTTCGGCACT GCAGAACGCC GCCTCGATCG

Citri TCGGCATCCT GGACCCGACC AAGGTGACCC GCTCTGCGCT GCAGAACGCC GCCTCGATCG

Oryzae TCGGCATCCT GGACCCGACC AAGGTCACCC GTTCGGCGCT GCAGAACGCC GCCTCGATCG

Campestris TCGGCATCCT GGACCCGACC AAGGTCACCC GTTCGGCGCT GCAGAACGCA GCATCGATCG

Xyl9a5C TTGGCATCCT GGACCCCACC AAGGTCACCC GTTCAGCACT CCAGAACGCT GCTTCAATCG

XylTemecul TTGGCATCCT GGACCCAACC AAGGTCACCC GTTCAGCACT CCAGAACGCT GCTTCAATCG

Burkholder CCGGCGTCGT CGATCCGACG AAGGTCACCC GTACCGCGCT GCAGAACGCG GCTTCGGTCG

Ralstonia TGGGCGTGCT GGACCCGACC AAGGTGACCC GCACCGCGCT GCAGAACGCC GCTTCGGTCG

....|....| ....|....| ....|....| ....|....| ....|....| ....|....|

8165 8175 8185 8195 8205 8215

X.albiline CTGGCCTGAT GATCACCACC GAGGCGATGG TGGCCGAAGC GCCGAAGAAG GACGAGCCGG

StenoK279a CTGGCCTGAT GATCACCACC GAAGCCATGG TTGCCGAAGC TCCGAAGAAG GACGAGCCGG

StenoR551 CTGGCCTGAT GATCACCACC GAAGCCATGG TTGCCGAAGC TCCGAAGAAG GACGAGCCGG

Vesicatori CCGGCCTGAT GATCACCACC GAAGCCATGG TGGCCGATGC GCCGAAGAAG GACGAGCCGG

Citri CCGGCCTGAT GATCACCACC GAAGCCATGG TGGCCGATGC ACCGAAGAAG GACGAGCCGG

Oryzae CCGGCCTGAT GATCACCACC GAAGCCATGG TGGCCGATGC ACCGAAGAAG GACGAGCCGG

Campestris CCGGCCTGAT GATCACCACC GAAGCCATGG TGGCCGATGC ACCGAAGAAG GACGAGCCGG

Xyl9a5C CTGGCCTGAT GATCACCACA GAAGCAATGG TCGCCGAGGC TCCGAAGAAA GACGAGCCAA

XylTemecul CTGGCCTGAT GATCACCACA GAAGCAATGG TCGCCGAGGC TCCGAAGAAA GACGAGCCAA

Burkholder CCGGCCTGCT GCTGACGACG GACGCAGCCG TTGCCGAACT GCCGAAGGAA GACG---CTC

Ralstonia CATCGCTGAT GCTGACGACG GACTGCGCCG TGGCCGAACT GCCGAAGGAC GATG---CAG

....|....| ....|....| ....|....| ....|....| ....|....| ....|....|

8225 8235 8245 8255 8265 8275

X.albiline CCGCGCCGGG CGGTATGGGC GGTGGCATGG GTGGCATGGG ---------C GGCATGGATT

StenoK279a CCATGGGCGG CGC------C GGTGGCATGG GCGGCATGGG TGGCATGGGC GGCATGGACT

StenoR551 CCATGGGTGG CGC------C GGTGGCATGG GCGGCATGGG TGGCATGGGC GGCATGGACT

Vesicatori CGATGCCGGC CGG------C GGCGGCATGG GCGGCATGGG ---------C GGCATGGATT

Citri CACTGCCGGC CGG------C GGTGGCATGG GCGGCATGGG ---------C GGCATGGATT

Oryzae CAATGCCGGC CGG------C GGTGGCATGG GCGGCATGGG ---------C GGCATGGATT

Campestris CGATGCCGGC CGG------C GGTGGCATGG GCGGCATGGG ---------C GGCATGGATT

Xyl9a5C CCCCACCTGC TGCT---GGT GGCGGTATGG GCGGTATGGG ---------C GGTATGGATT

XylTemecul CCCCACCTGC TGCT---GGT GGCGGCATGG GCGGTATGGG ---------C GGTATGGATT

Burkholder CGATGCCGGG CGGCATGCCG GGCGGCATGG GCGGCATGGG CATGGGCATG GGCATGGACA

Ralstonia C----TCCGG CA--ATGCCG GGCGGCATGG GTGGCATGGG C---GGCATG GACGGCATGA

....|....| ....|....| ....|....| ....|....| ....|....| ....|....|

8285 8295 8305 8315 8325 8335

X.albiline TCTGAATG-- ---------- ---------- ---------- ---------- ----------

StenoK279a TCTAAATG-- ---------- ---------- ---------- ---------- ----------

StenoR551 TCTAAATG-- ---------- ---------- ---------- ---------- ----------

Vesicatori TCTGAATG-- ---------- ---------- ---------- ---------- ----------

Citri TCTGAATG-- ---------- ---------- ---------- ---------- ----------

Oryzae TCTGAATG-- ---------- ---------- ---------- ---------- ----------

Campestris TCTGAATG-- ---------- ---------- ---------- ---------- ----------

Xyl9a5C TCTAAATGCT TACCCGAAAA AAATCAATGG ATTGCAAGGT TGGCAGACTT GGTATGCCAA

XylTemecul TCTAAATG-- ---------- ---------- ---------- ---------- ----------

Burkholder TGTAAATGAG TAA------- ---------- ---------- ---------- ----------

Ralstonia TGTAAATGTC CCA------- ---------- ---------- ---------- ----------

....|....| ....|....| ....|....| ....|....| ....|....| ....|....|

8345 8355 8365 8375 8385 8395

X.albiline ---------- ---------- ---------- ---------- ---------- ---TCTGCGG

StenoK279a ---------- ---------- ---------- ---------- ---------- ---TCCGTGG

StenoR551 ---------- ---------- ---------- ---------- ---------- ---TCCGTGG

Vesicatori ---------- ---------- ---------- ---------- ---------- ---TCCGTGG

Citri ---------- ---------- ---------- ---------- ---------- ---TCCGTGG

Oryzae ---------- ---------- ---------- ---------- ---------- ---TCCGTGG

Campestris ---------- ---------- ---------- ---------- ---------- ---TCCGTGG

Xyl9a5C TTGCTTTAGC TGTTTCTGAC TGTTACCCCA ATCCTAAAAG GTTTCCTCTG ATGTCGCTGG

XylTemecul ---------- ---------- ---------- ---------- ---------- ---TCGCTGG

Burkholder ---------- ---------- ---------- ---------- ---------- --AACCGTCG

Ralstonia ---------- ---------- ---------- ---------- ---------- --AAGCATTG

....|....| ....|....| ....|....| ....|....| ....|....| ....|....|

8405 8415 8425 8435 8445 8455

X.albiline AAACTATTGA GAAGTTGGTT AAGGACAACA AGATCGAGTT CGTCGACCTG CGCTTCGTCG

StenoK279a AAAACGTTGA GAAGCTGATC AAGGACAACC AGATCGAATT CGTCGATCTG CGCTTTGTCG

StenoR551 AAAACGTTGA GAAGCTGATC AAGGACAACC AGATCGAGTT CGTCGACCTG CGCTTTGTCG

Vesicatori AAAATGTTGA AAAGCTGATC AAGGACAACA AGGTCGAGTT CGTCGACCTG CGCTTCGTCG

Citri AAAATGTTGA AAAGCTGATC AAGGACAACA AGGTCGAGTT CGTCGACCTG CGCTTCGTCG

Oryzae AAAATGTTGA AAAGCTGATC AAGGACAACA AGGTCGAGTT CGTCGACCTG CGCTTCGTCG

Campestris AAAATGTTGA AAAGCTGATC AAGGACAACA AGGTCGAGTT TGTCGACCTG CGCTTCGTCG

Xyl9a5C AAAATGTTGA AAAGCTTGTC AAGGATAAAA AGATCGAATT CGTCGATTTG CGCTTTGTTG

XylTemecul AAAATGTTGA AAAGCTTGTC AAGGATAAAA AGATCGAATT CGTCGATTTG CGCTTTGTTG

Burkholder CCGACGTCAT GCAGCTCGCG AAGGACGAGG ACGTCAAGTT TGTCGATTTC CGCTTCACGG

Ralstonia CAGACGTGAT GAAGCTCGTG AAGGAAAACG ACGTCAAGTT CGTCGATTTC CGCTTCACCG

....|....| ....|....| ....|....| ....|....| ....|....| ....|....|

8465 8475 8485 8495 8505 8515

X.albiline ACCTGCGCGG CGTGCAGCAG CACGTCACGT TCCCGGTCAG CATCATCGAG CCTGCATTGT

StenoK279a ACATGCGTGG TGTCGAGCAG CACGTGACCT TCCCGGTCAG CATCGTCGAA CCGTCGCTGT

StenoR551 ACATGCGTGG TGTCGAGCAG CACGTGACCT TCCCGGTCAG CATCGTCGAG CCGTCGCTGT

Vesicatori ATATGCGTGG TGTACAGCAG CACATCACCT TTCCGGTCAA TATCATCGAG CCGGCGCTGT

Citri ATATGCGTGG TGTACAGCAG CACATCACCT TTCCGGTCAA TATCATCGAG CCGGCGCTGT

Oryzae ATATGCGTGG TGTACAGCAG CACATCACCT TTCCGGCCAA TATCATCGAG CCGGCGCTGT

Campestris ATATGCGTGG CGTGCAGCAG CACATCACCT TTCCGGCCAG CATCATCGAG CCGGCGTTGT

Xyl9a5C ATATGCGCGG CGTGCAACAG CACGTCACCT TTCCGGTGAA CATTCTTGAA CCATCGCTGT

XylTemecul ATATGCGCGG CGTACAACAG CACGTCACCT TTCCGGTGAA CATTCTTGAA CAATCGCTGT

Burkholder ACACGCGCGG CAAGGAGCAA CACGTGTCGG TGCCGGTGTC GGCCTTCGGC GAGGACAAGT

Ralstonia ATACCAAGGG TAAAGAGCAG CACGTCTCGG TTCCCGTGTC GCATTTCGGC GAAGACAAGT

....|....| ....|....| ....|....| ....|....| ....|....| ....|....|

8525 8535 8545 8555 8565 8575

X.albiline TCGAAGAGGG CAAGATGTTC GACGGCAGCT CGATCGCCGG CTGGAAGGGC ATCAATGAGT

StenoK279a TCGAAGAAGG CAAGATGTTC GATGGCAGCT CGATCGCCGG CTGGAAGGGC ATCAACGAGT

StenoR551 TCGAAGAAGG CAAGATGTTC GATGGCAGCT CGATCGCCGG CTGGAAGGGC ATCGCCGAAT

Vesicatori TTGAAGAAGG CAAGATGTTC GACGGCAGTT CGATCGCGGG CTGGAAGGGC ATCAACGAGT

Citri TTGAAGAAGG CAAGATGTTC GACGGCAGTT CGATCGCGGG CTGGAAGGGC ATCAACGAGT

Oryzae TTGAAGAAGG CAAGATGTTC GACGGCAGTT CGATCGCGGG TTGGAAGGGC ATCAACGAGT

Campestris TCGAAGAAGG CAAGATGTTC GACGGCAGCT CCATCGCGGG TTGGAAGGGC ATCAACGAGT

Xyl9a5C TCAAGGATGG AAAGATGTTC GACGGTAGCT CGATTGCCGG TTGGAAGGGG ATCAATGAGT

XylTemecul TCAAGGATGG AAAGATGTTC GACGGTAGCT CGATTGCTGG TTGGAAGGGG ATCAATGAGT

Burkholder TCGAAAGCGG CCACGCGTTC GACGGCTCGT CGATCGCGGG CTGGAAGGGC ATCGAGGCGT

Ralstonia TCGAAAGCGG CCACGCATTC GACGGCTCGT CGATCGCCGG CTGGAAGGGC ATCGAAGCTT

....|....| ....|....| ....|....| ....|....| ....|....| ....|....|

8585 8595 8605 8615 8625 8635

X.albiline CGGACATGGT TCTGCTGCCG GATGCCGACA CCGCTTTCCT CGACCCGTTC ATGGCTGACC

StenoK279a CGGACATGGT GCTGCTGCCG GATACCGCCA GCGCCTACGT CGACCCGTTC TACGCCGATC

StenoR551 CGGACATGGT GCTGCTGCCG GATACCGCCA GCGCCTACGT CGACCCGTTC TACGCCGATC

Vesicatori CGGACATGGT GTTGCTGCCG GACGCGGGCA CCGCGTATCT GGATCCGTTC TTCGCCGATC

Citri CGGACATGGT GTTGCTGCCG GACGCGGGCA CCGCGTATCT GGATCCGTTC TTCGCCGATC

Oryzae CGGACATGGT GCTGCTGCCG GACGCAGGCA CCGCGTATCT GGATCCGTTC TTCGCCGATC

Campestris CGGACATGGT CCTGCTCCCG GACGCGGGCA CCGCTTACCT GGATCCGTTC TTCGCCGATC

Xyl9a5C CGGACATGGT ACTGCTGCCT GACGCTAACA CCGCCTATCT GGATCCTTTT TACGCTGATC

XylTemecul CGGACATGGT ACTGCTGCCT GACGCTAGCA CCGCTTATCT GGATCCTTTT TACGCTGATC

Burkholder CGGACATGCT GCTCATGCCG GATCCGAACG CGGCCTTCAT CGACCCGTTC TACGAAGAGT

Ralstonia CGGACATGCT GCTGATGCCG GATGCGAACA CCGCCCACAT CGACCCGTTC TACGAAGAGC

....|....| ....|....| ....|....| ....|....| ....|....| ....|....|

8645 8655 8665 8675 8685 8695

X.albiline CGACCCTGGT GCTGACCTGC GACATCCTCG ATCCGGCGAC CATGCAGAGC TATGCGCGCG

StenoK279a CGACCATCGT GATCAGCTGC GACATCCTCG ACCCGGCCAC CATGCAGCCG TATGGCCGTT

StenoR551 CGACCATCGT GATCAGCTGC GACATCCTCG ATCCGGCCAC CATGCAGCCG TATGGCCGTT

Vesicatori CGACCATCGT GCTGACCTGC GACATCCTCG ACCCGGCCAC CATGCAGAGC TATGAGCGCG

Citri CGACCATCGT GCTGACCTGC GACATCCTCG ACCCGGCCAC CATGCAGAGC TATGAGCGCG

Oryzae CGACCATCGT GCTGACCTGC GACATCCTTG ACCCGGCCAC CATGCAGAGC TATGAGCGCG

Campestris CGACCATCGT GCTGACCTGC GACATCCTCG ACCCGGCCAC CATGCAGAGC TACGAGCGCG

Xyl9a5C CAACGCTGGT CATGACATGC GACATCCTCG ATCCAGCGAC GATGCAGAGT TATGTGCGTG

XylTemecul CAACGCTGGT CATGACATGC GACATCCTTG ATCCAGCGAC GATGCAGAGT TATGTGCGTG

Burkholder CGACCCTCGT GCTGACCTGC GATGTCGTCG AGCCGGCGGA CGGCAAGGGC TACGAGCGCG

Ralstonia CGACCCTGGT CATGACCTGC GACGTGATCG AACCATCGGA CGGCAAGGGC TACGACCGCG

....|....| ....|....| ....|....| ....|....| ....|....| ....|....|

8705 8715 8725 8735 8745 8755

X.albiline ATCCGCGCGG CGTGGCCAAG CGCGCCGAGG CTTATCTGAA GTCCAGCGGC ATCGCCGATC

StenoK279a GCCCGCGCGG CATCGCCAAG CGCGCCGAGG CCTACCTGAA GTCCTCCGGC ATTGCCGAAA

StenoR551 GCCCGCGCGG CATCGCCAAG CGGGCCGAGG CCTACCTGAA GTCCTCCGGC ATCGCCGAAA

Vesicatori ACCCGCGCGG CATCGCCAAG CGCGCCGAGG CCTACCTGAA GTCCTCCGGC ACCGCAGACC

Citri ATCCGCGCGG CATCGCCAAG CGCGCCGAGG CCTATCTGAA ATCCTCCGGC ACCGCCGACC

Oryzae ATCCGCGCGG CATCGCCAAG CGCGCCGAGG CCTATCTGAA GTCGTCCGGC ACCGCCGACC

Campestris ATCCGCGCGG CATTGCAAAG CGTGCCGAGG CCTATCTGAA GTCGTCCGGC ACCGCCGATC

Xyl9a5C ATCCCCGAGG CATCGCCAAA CGTGCCGAAA TCTACTTAAA TTCTTTGGGT ATTGCTGACC

XylTemecul ATCCCCGAGG TATCGCCAAA CGTGCCGAAA TCTACTTAAA TTCTTTAGGT ATTGCTGACC

Burkholder ATCCGCGCTC GCTCGCGAAG CGCGCCGAGG CCTACCTGAA GAGCACGGGC ATCGGCGACA

Ralstonia ACCCGCGCTC GATCGCCAAG CGCGCTGAAG CCTACCTGAA GAGCACCGGC CTGGGCGACA

....|....| ....|....| ....|....| ....|....| ....|....| ....|....|

8765 8775 8785 8795 8805 8815

X.albiline AGGCGTTCTT CGGCCCGGAG CCGGAATTCT TCATCTTCGA CGGCGTGCGT TTCGGCAACG

StenoK279a CCGCGTTCTT CGGCCCGGAG CCGGAATTCT TCATCTTCGA CTCGGTCCGT TTCGCCAATG

StenoR551 CCGCGTTCTT CGGCCCGGAG CCGGAATTCT TCATCTTCGA TTCGGTCCGC TTCGCCAATG

Vesicatori AGGCGTTCTT CGGCCCGGAG CCGGAATTCT TCATCTTCGA TTCGGTGCGC TTTGCCAACG

Citri AGGCGTTCTT CGGCCCGGAG CCGGAATTCT TCATCTTCGA TTCGGTGCGC TTTGCCAACG

Oryzae AGGCGTTCTT CGGCCCGGAG CCGGAATTCT TCATCTTCGA TTCGGTGCGC TTTGCCAACG

Campestris AGGCGTTCTT TGGCCCGGAG CCGGAATTCT TCATCTTCGA TTCGGTGCGC TTCGCCAATG

Xyl9a5C AAGCATTTTT CGGTCCGGAA CCTGAATTTT TTATCTTCGA TTCAGTCCGT TTTGCTAATG

XylTemecul AAGCATTTTT CGGTCCGGAA CCTGAATTTT TTATCTTCGA TTCAGTCCGT TTTGCTAATG

Burkholder CCGCGTACTT CGGTCCGGAG CCGGAATTCT TCATTTTCGA CTCGGTGCAG TGGAACACGG

Ralstonia CCGCCTACTT CGGTCCGGAA CCCGAATTCT TCATCTTCGA CGGCGTGACC TGGAACGTCG

....|....| ....|....| ....|....| ....|....| ....|....| ....|....|

8825 8835 8845 8855 8865 8875

X.albiline ACATGGGCCA CACCTTCTTC AAGATCGATT CGGAAGAAGC GGCTTGGAGC AGCGGCAGCA

StenoK279a AAATGGGCAA CACTTTCTTC AAGGTCGATT CCGAAGAAGC CGCCTGGAAC AGCGGCGCCA

StenoR551 AAATGGGCCA CACCTTCTTC CAGGTCGATT CGGAAGAAGC GGCGTGGAAC AGCGGCGCCA

Vesicatori ACATGGGCCA CACCTTCTTC CAGGTGGGTT CGGAAGAGGC GGCCTGGAAC ACCGGCGAAA

Citri ACATGGGCCA CACCTTCTTC CAGGTGGGTT CGGAAGAAGC GGCCTGGAAC ACCGGCGAAA

Oryzae ACATGGGCCA CACCTTCTTC CAGGTGGGTT CGGAAGAAGC GGCCTGGAAT ACCGGTGAAA

Campestris ACATGGGCCA CACCTTCTTC CAGGTGGGTT CGGAAGAGGC GGCCTGGAAC ACCGGCGCCA

Xyl9a5C ATATGGGTCA TGCTTTTTTC CAAATTGCTT CCGAAGAGGC TGCCTGGAAC ACAGGCGCTG

XylTemecul ATATGGGTCA TGCTTTTTTC CAAATTGCTT CCGAAGAGGC TGCCTGGAAC ACAGGCGCTG

Burkholder ACATGTCCGG CTGCTTCGTG AAGATCAACT CGGAAGAAGC GCCGTGGTCG TCGGCGAAGG

Ralstonia ACATGCAAGG CTCCTTCGTG AAGATCCATT CCGAAGAAGC GCCGTGGTCG TCGGGCAAGG

....|....| ....|....| ....|....| ....|....| ....|....| ....|....|

8885 8895 8905 8915 8925 8935

X.albiline AGATCGAGGG TGGCAACAGC GGTTATCGTC CGGCGGTCAA GGGCGGCTAC TTCCCGGTGC

StenoK279a AGTACGACGG CGCCAACAGC GGTTACCGTC CGGGCGTGAA GGGTGGCTAT TTCCCGGTTC

StenoR551 AGTACGACGG CGCCAACAGC GGCTACCGTC CGGGCGTGAA GGGCGGTTAC TTCCCGGTGC

Vesicatori AGTACGACGG CGGCAACAGC GGTTACCGCC CGGGCGTGAA GGGCGGCTAC TTCCCGGTTC

Citri AGTACGACGG CGGCAACAGC GGTTACCGCC CGGGCGTGAA GGGCGGCTAC TTCCCGGTTC

Oryzae AGTACGACGG CGGCAATAGC GGCTACCGTC CGGGCGTGAA GGGCGGCTAC TTCCCGGTTC

Campestris AGTACGACGG CGGCAACAGC GGTTACCGTC CCGGCGTGAA GGGTGGCTAC TTCCCGGTGC

Xyl9a5C AGTTTGAGGG AGGTAACGGT GGTTACCGCC CGGCGGTAAA GGGTGGATAC TTCCCAGTTC

XylTemecul AGTTTGAGGG AGGTAACAGT GGTTACCGCC CGACGGTAAA GGGTGGATAC TTCCCAGTTC

Burkholder AATTCGAAGG CGGCAACACG GGCCACCGTC CGGGCGTGAA GGGCGGCTAC TTCCCGGTTG

Ralstonia AATTCGAGCA CGGCAACTCC GGCCACCGTC CGGGCAAGAA GGGCGGCTAC TTCCCGGTCG

....|....| ....|....| ....|....| ....|....| ....|....| ....|....|

8945 8955 8965 8975 8985 8995

X.albiline CGCCGACCGA CTCGCTGCAG GATCTGCGCG CGGAAATGTG CAAGACCCTG GAGCAGGTCG

StenoK279a CGCCGACCGA CACCCTGCAC GACCTGCGCG CCGAGATGTG CAAGACCCTG GAACAGGTCG

StenoR551 CGCCGACCGA CACCCTGCAC GACCTGCGCG CCGAGATGTG CAAGACCCTG GAACAGGTCG

Vesicatori CGCCGACCGA CAGCCTGCAC GACCTGCGCG CGGAAATGAT CAAGACGCTG GAACAGGTCG

Citri CGCCGACCGA CAGCCTGCAC GACCTGCGCG CGGAAATGAT CAAGACGCTG GAACAGGTCG

Oryzae CGCCGACCGA CACCCTGCAC GACCTGCGCG CGGAAATGAT CAAGACGCTG GAACAGGTCG

Campestris CGCCGACCGA CACCCTGCAT GACCTCCGTG CGGAAATGAT CAAGACGCTG GAGCAGGTCG

Xyl9a5C CACCGACTGA CTCGCTGCAC GATTTGCGTG CCGAGATGGT GAAGACCCTG GAGCAGGTTG

XylTemecul CACCGACTGA CTCGCTGCAC GATTTGCGTG CCGAGATGGT GAAGACCCTG GAGCAGGTTG

Burkholder CGCCGGTCGA CCAGTTCCAG GACATGCGCT CGGAAATGTG TCTGCTGCTC GAGCAGATCG

Ralstonia CCCCGATCGA CACGTTCCAG GACATCCGCT CGGAAATGTG CCTGATCCTG GAATCGCTGG

....|....| ....|....| ....|....| ....|....| ....|....| ....|....|

9005 9015 9025 9035 9045 9055

X.albiline GCATCGAGGT CGAAGTACAC CACCACGAGG TCGCCACCGC TGGCCAGTGC GAGATCGGCA

StenoK279a GCATCGAAGT GGAAGTGCAG CACCACGAAG TGGCCACCGC CGGCCAGTGC GAGATCGGCA

StenoR551 GCATCGAAGT GGAAGTGCAG CACCACGAAG TGGCCACCGC CGGCCAGTGC GAGATTGGCA

Vesicatori GCATCGAAAC CGAGGTGCAC CACCACGAAG TGGCCACCGC CGGCCAGTGC GAGATCGGCA

Citri GCATCGAAAC CGAAGTGCAC CACCACGAAG TGGCCACCGC CGGCCAGTGC GAGATCGGCA

Oryzae GCATCGAAAC CGAAGTGCAC CACCACGAAG TGGCCACCGC CGGTCAGTGC GAAATCGGCA

Campestris GCATCGAGAC CGAGGTGCAC CACCACGAGG TGGCGACCGC CGGCCAGTGC GAGATCGGCA

Xyl9a5C GTATTGAGAC CGAGGTGCAT CATCACGAAG TCGCCACTGC TGGACAATGT GAGATCGGCA

XylTemecul GCATTGAGAC CGAGGTGCAT CATCACGAAG TCGCCACTGC TGGACAATGT GAGATCGGCA

Burkholder GCATTCCGGT CGAAGTGCAC CACCACGAGG TGGCGGGCCA GGGCCAGAAC GAAATCGGCA

Ralstonia GCATTCCGGT TGAAGTGCAT CACCACGAAG TGGCGGGCCA AGGCCAGAAC GAAATCGGCA

....|....| ....|....| ....|....| ....|....| ....|....| ....|....|

9065 9075 9085 9095 9105 9115

X.albiline CCAAGTTCAA CTCGCTGGTG AAGAAGGCCG ACGAGCTGAT GACGATGAAG TACATCATCA

StenoK279a CCAAGTTCAG CACCCTGGTG CAGAAGGCCG ACGAACTGCT GCGCATGAAG TACGTGATCA

StenoR551 CCAAGTTCAG CACCCTGGTG CAGAAGGCCG ACGAACTGCT GCGCATGAAG TACGTGATCA

Vesicatori CCAAGTTCAG CTCGCTGGTG CAGAAGGCCG ACGAACTGCT GACGATGAAG TACATCATCA

Citri CCAAGTTCAG CTCGCTGGTG CAGAAGGCCG ACGAACTGCT GACGATGAAG TACATCATCA

Oryzae CCAAGTTCAG CTCGCTGGTG CAGAAGGCCG ACGAACTGCT GACGATGAAG TACATCATCA

Campestris CCAAGTTCAG CTCGCTGGTG CAAAAAGCCG ACGAACTGCT GACGATGAAG TACATCATCA

Xyl9a5C CTAAGTTCAA CTCATTGGTC AAAAAGGCTG ATCAGCTGAT GACGATGAAA TACATCCTTA

XylTemecul CTAAGTTCGA CTCATTGGTC AAAAAGGCTG ATCAGCTGAT GACGATGAAA TACATCCTTA

Burkholder CGAAGTTCTC GACGCTCGTC GAGCGCGCGG ACTGGACGCA ATGGGCGAAG TACATCATCC

Ralstonia CGAAGTTCAG CACGCTGGTG CAGCGCGCCG ACTGGACCCA GCTGCAAAAG TACGTGATCC

....|....| ....|....| ....|....| ....|....| ....|....| ....|....|

9125 9135 9145 9155 9165 9175

X.albiline AGAACGTTGC CTACCGCAAC GGCAAGACTG TGACCTTCAT GCCCAAGCCG ATCGTCGGCG

StenoK279a AGAACGTCGC GCACCGCAAC GGCAAGACCG TCACCTTCAT GCCCAAGCCG ATCGTCGGCG

StenoR551 AGAACGTCGC GCACCGCAAC GGCAAGACCG TCACCTTCAT GCCCAAGCCG ATCGTCGGCG

Vesicatori AGAACGTCGC CTACCGCAAC GGCAAGACCG CCACCTTCAT GCCCAAGCCC ATCGTGGGCG

Citri AGAACGTCGC CTACCGCAAC GGCAAGACCG CCACCTTCAT GCCCAAGCCC ATCGTGGGCG

Oryzae AGAACGTCGC CTACCGCAAC GGCAAGACCG CCACCTTCAT GCCCAAGCCC ATCGTGGGCG

Campestris AGAACGTCGC CTACCGCAAC GGCAAGACCG CCACCTTCAT GCCGAAGCCG ATCGTCGGCG

Xyl9a5C AAAACGTTGC TTACCGCAAT GGAAAGACTG TCACCTTTAT GCCTAAGCCG ATGGTTGGTG

XylTemecul AAAACGTTGC TTACCGCAAT GGGAAGACTG TCACCTTTAT GCCTAAACCG ATCGTTGGTG

Burkholder ATAACGTCGC GCACTCGTAC GGCAAGACGG CGACGTTCAT GCCGAAGCCC GTCGTCGGCG

Ralstonia AGAACGTCGC GCACACCTAC GGCAAGACCG CCACGTTCAT GCCGAAGCCG GTCGTGGGCG

....|....| ....|....| ....|....| ....|....| ....|....| ....|....|

9185 9195 9205 9215 9225 9235

X.albiline ACAACGGCTC GGGCATGCAT GTGCACCAGT CGCTGGCCAA GGGCGGCACC AACCTGTTCT

StenoK279a ACAACGGCAG CGGCATGCAC GTGCACCAGT CGCTGTCCAA GGGCGGCACC AACCTGTTCT

StenoR551 ACAACGGCAG CGGCATGCAC GTGCACCAGT CGCTGTCCAA GGGCGGCACC AACCTGTTCT

Vesicatori ACAACGGCTC GGGCATGCAC GTGCACCAGT CGCTGGCCAA GGGCGGCGTC AACCTGTTCA

Citri ACAACGGCTC GGGCATGCAC GTGCACCAGT CGCTGGCCAA GGGCGGCGTC AACCTGTTCA

Oryzae ACAACGGCTC GGGCATGCAC GTGCACCAGT CGCTGGCCAA GGGCGGCGTC AACCTGTTCA

Campestris ACAACGGCTC GGGCATGCAC GTGCACCAGT CGCTGACCAA GGGCGGCGTG AACCTGTTCT

Xyl9a5C ATAACGGTTC TGGCATGCAT GTGCATCAGT CGCTCTCTAA AGGAGGGGTC AACTTGTTCT

XylTemecul ATAACGGTTC TGGCATGCAT GTACATCAGT CGCTCTCTAA AGGAGGGGTC AACTTGTTCT

Burkholder ACAACGGCTC GGGCATGCAC GTGCACCAGT CGATCTGGAA GGACGGCCAG AACCTGTTCG

Ralstonia ACAACGGTTC GGGTATGCAC GTGCACCAGT CCGTGTGGAA GGACGGCCAG AACCTGTTCG

....|....| ....|....| ....|....| ....|....| ....|....| ....|....|

9245 9255 9265 9275 9285 9295

X.albiline CCGGCGACGG CTATGGTGGC CTGTCGCAGA TGGCGCTGTG GTACATCGGC GGCATCTTCA

StenoK279a CCGGTGACGG CTACGGCGGC CTGAGCCAGC TGGCGCTGTG GTACATCGGC GGCATCTTCA

StenoR551 CCGGCGACGG CTACGGCGGC CTGAGCCAGC TGGCGCTGTG GTACATCGGC GGCATCTTCA

Vesicatori CCGGCGACGG CTACGGCGGC CTGTCGCAGC TGGCGCTGTG GTACATCGGC GGCATCTTCA

Citri CCGGCGACGG CTACGGCGGC CTGTCGCAGC TGGCGCTGTG GTACATCGGC GGCATCTTCA

Oryzae CCGGCGACGG CTACGGCGGT TTGTCGCAGC TGGCGCTGTG GTACATCGGC GGCATCTTTA

Campestris CCGGCGACGG CTACGGCGGC CTGTCGCAGA TGGCGCTGTG GTACATCGGC GGCATCTTCA

Xyl9a5C CCGGTGACGA CCATGCTGGT TTGTCGCAGC TTGCACTGTA TTACATCGGC GGTATTTTCA

XylTemecul CCGGTGACGA CCATGCTGGT TTGTCGCAGC TGGCACTGTA TTACATCGGC GGTATTTTCA

Burkholder CGGGCAACGG CTACGCGGGC CTGTCGGAGA CGGCGCTCTT CTACATCGGC GGCATCATCA

Ralstonia CAGGCAACGG CTACGCCGGC CTGTCGGAAT TCGCGCTGTA CTACATCGGC GGCATCATCA

....|....| ....|....| ....|....| ....|....| ....|....| ....|....|

9305 9315 9325 9335 9345 9355

X.albiline AGCATGCCAA GGCGATCAAC GCCTTCACCA ACTCGGGCAC CAACAGCTAC AAGCGTCTGG

StenoK279a AGCACGCCAA GGCCATCAAT GCCTTCGCCA ACTCGGGCAC CAACAGCTAC AAGCGCCTGG

StenoR551 AGCATGCCAA GGCCATCAAT GCCTTCGCCA ACTCGGGCAC CAACAGCTAC AAGCGCCTGG

Vesicatori AGCATGCCCG CGCGATCAAC GCCTTCGCCA ACTCCGGCAC CAACAGCTAC AAGCGCCTGG

Citri AGCATGCCCG CGCGATCAAC GCCTTCGCCA ACTCCGGCAC CAACAGCTAC AAGCGTCTGG

Oryzae AGCACGCCCG CGCGATCAAC GCCTTCGCCA ACTCCGGCAC CAACAGCTAC AAGCGTCTGG

Campestris AGCACGCCCG CGCCATCAAT GCCTTCGCCA ACTCCGGTAC CAACAGCTAC AAGCGTTTGG

Xyl9a5C AGCATGCTCA TGCGATCAAC GCGTTTTCCA ATTCGGGCAC AAATAGCTAT AAGCGTTTGG

XylTemecul AGCACGCTCA TGCGATCAAC GCGTTTTCCA ATTCGGGAAC AAATAGCTAT AAGCGTTTGG

Burkholder AGCACGCGCG CGCGCTGAAC GCGATCACGA ACCCGACGAC GAACTCGTAC AAGCGTCTCG

Ralstonia AGCACGCTCG CGCGCTGAAC GCCATCACCA ACCCGGGCAC GAACTCGTAC AAGCGTCTGG

....|....| ....|....| ....|....| ....|....| ....|....| ....|....|

9365 9375 9385 9395 9405 9415

X.albiline TCCCGGGCTT CGAGGCGCCG GTGATGCTGG CCTACTCGGC GCGCAACCGC TCGGCCTCGT

StenoK279a TGCCGGGCTT CGAAGCGCCG GTGATGCTGG CCTATTCGGC CCGCAACCGT TCGGCATCGT

StenoR551 TGCCGGGCTA CGAAGCACCG GTGATGCTGG CCTACTCGGC CCGCAACCGT TCGGCTTCGT

Vesicatori TTCCGGGCTT CGAGGCGCCG GTGATGCTGG CCTATTCGGC GCGTAACCGT TCGGCCTCGT

Citri TCCCGGGCTT CGAGGCGCCG GTGATGCTGG CCTATTCGGC GCGTAACCGT TCGGCGTCGT

Oryzae TTCCGGGCTT CGAGGCACCA GTGATGCTGG CCTATTCGGC GCGTAACCGC TCGGCGTCGT

Campestris TTCCTGGTTA CGAAGCTCCG GTGATGCTGG CCTATTCGGC GCGTAACCGA TCGGCCTCGT

Xyl9a5C TTCCGGGCTT CGAAGCACCC GTGATGTTGG CTTATTCGGC ACGTAACCGC TCGGCCTCGT

XylTemecul TTCCGGGCTT CGAAGCACCC GTAATGTTGG CTTATTCGGC ACGTAACCGC TCGGCCTCGT

Burkholder TGCCGCACTT CGAAGCGCCC GTGAAGCTCG CCTACTCGGC GCGCAACCGC TCGGCGTCGA

Ralstonia TGCCGGGCTT CGAAGCACCG GTCAAGCTGG CTTACTCGGC TCGCAACCGT TCGGCTTCGA

....|....| ....|....| ....|....| ....|....| ....|....| ....|....|

9425 9435 9445 9455 9465 9475

X.albiline GCCGCATCCC GTGGGTGTCC AACCCGAAGG CGCGCCGCAT CGAGATCCGC TTCCCGGATC

StenoK279a GCCGCATTCC GTGGGTCTCC AACCCGAAGG CGCGCCGTAT CGAAATGCGC TTCCCGGACC

StenoR551 GCCGCATTCC GTGGGTGTCC AACCCGAAGG CACGCCGCAT CGAAATGCGC TTCCCGGATC

Vesicatori GCCGCATTCC GTGGGTCACC AACCCGAAGG CGCGCCGCAT CGAAATGCGC TTCCCCGATC

Citri GCCGCATTCC CTGGGTCACC AATCCGAAGG CGCGCCGCAT CGAAATGCGC TTCCCCGATC

Oryzae GCCGTATTCC GTGGGTCACC AACCCGAAGG CGCGCCGCAT CGAAATGCGC TTCCCCGATC

Campestris GCCGCATTCC GTGGGTGACC AACCCGAAGG CGCGCCGCAT CGAAATGCGC TTCCCCGATC

Xyl9a5C GCCGTATTCC ATGGGTTTCT AGCCCGAAGG CACGCCGCAT TGAAATGCGT TTCCCCGATC

XylTemecul GCCGTATTCC ATGGGTTTCT AGCCCGAAGG CACGCCGCAT TGAAATGCGT TTCCCCGATC

Burkholder TCCGCATTCC GCACGTGTCG AACCCGAAGG GCCGCCGCAT CGAAACGCGC TTCCCGGACC

Ralstonia TCCGGATCCC GTACGTTGCC AACCCGAAGG GTCGCCGCAT CGAAACGCGC TTCCCGGATC

....|....| ....|....| ....|....| ....|....| ....|....| ....|....|

9485 9495 9505 9515 9525 9535

X.albiline CGCTGCAATC CGGCTACCTG ACCTTTGCCG CGTTGATGAT GGCAGGCTTG GACGGTATCA

StenoK279a CGATCCAGTC CGGCTACCTC ACCTTCACCG CGCTGATGAT GGCCGGCCTG GACGGCATCA

StenoR551 CGATCCAGTC CGGCTACCTC ACCTTCACCG CGCTGATGAT GGCCGGCCTG GACGGCATCA

Vesicatori CGTTGCAGTC CGGCTACCTG ACCTTCACCG CGCTGATGAT GGCCGGCCTG GACGGCATCA

Citri CGTTGCAGTC CGGCTACCTG ACCTTCACCG CGCTGATGAT GGCCGGCCTG GACGGCATCA

Oryzae CGTTGCAGTC CGGCTACCTG ACCTTCACCG CGCTGATGAT GGCCGGCTTG GACGGCATCA

Campestris CGTTGCAGTC CGGCTACCTG ACCTTCACCG CGCTGATGAT GGCCGGCCTG GACGGCATCA

Xyl9a5C CATTGCAGTC TGGTTACCTC ACCTTTACTG CGCTGATGAT GGCTGGGCTG GATGGCATTA

XylTemecul CATTGCAGTC TGGTTACCTC ACCTTTACTG CGCTGATGAT GGCTGGACTG GATGGCATTA

Burkholder CGATGGCGAA CCCGTACCTG TGCTTCTCGG CGCTGATGAT GGCGGGTCTC GACGGCATCC

Ralstonia CGCTGTGCAA CCCGTACCTG GGCTTTGCCG CGCTGCTGAT GGCCGGCCTG GACGGCGTGC

....|....| ....|....| ....|....| ....|....| ....|....| ....|....|

9545 9555 9565 9575 9585 9595

X.albiline AGAACCAGAT CGATCCAGGC GCGCCCAGCG ACAAGGATCT GTACGACCTG CCGCCGGAAG

StenoK279a AGAACCAGAT CGACCCGGGC GCACCGAGCG ACAAGGACCT GTACGACCTG CCGCCGGAAG

StenoR551 AGAACCAGAT CGACCCGGGC GCACCGAGCG ACAAGGACCT GTACGACCTG CCGCCGGAAG

Vesicatori AGAACCAGAT CGACCCGGGC GCCCCGAGCG ACAAGGACCT GTACGACCTG CCGCCGGAAG

Citri AGAACCAGAT CGATCCGGGC GCACCGAGCG ACAAGGACCT GTACGACCTG CCGCCGGAAG

Oryzae AGAACCAGAT CGACCCTGGC GCACCGAGCG ACAAGGACCT GTACGACCTG CCGCCGGAAG

Campestris AGAACCAGAT CGACCCGGGC GCACCGAGCG ACAAGGATCT GTACGACCTG CCGCCGGAAG

Xyl9a5C AAAACAAGAT CCATCCAGGC CCACCCAATG ACAAAGATCT GTATCACCTG CCGCCTGAAG

XylTemecul AAAACAAGAT CCATCCAGGC CCACCCAATG ACAAAGATCT GTATCACCTG CCGCCTGAAG

Burkholder AGAACAAGAT CCATCCGGGC GAGGCCGCGG ACAAGAACCT GTACGACCTG CCGCCGGAAG

Ralstonia AGAACAAGAT CCACCCGGGC GAAGCCGCCG ACAAGAACCT GTACGACCTG CCGCCGGAAG

....|....| ....|....| ....|....| ....|....| ....|....| ....|....|

9605 9615 9625 9635 9645 9655

X.albiline AAGAGAAGAA AATCCCGCAG GTCTGCTCCA GCCTCGACCA GGCGTTGGAA GCGCTTGATG

StenoK279a AAGAGAAGCT GATCCCGCAG GTCTGCTCCT CGCTGGACCA GGCGCTGGAA GCGCTGGACA

StenoR551 AAGAGAAGCT GATCCCGCAG GTCTGCTCCT CGCTGGACCA GGCGCTGGAA GCGCTGGACA

Vesicatori AAGAGAAGCT GATCCCGCAG GTGTGCTCCA GCCTGGACCA GGCCCTGGAA GCGCTGGACA

Citri AAGAGAAGCT GATCCCGCAG GTTTGCTCCA GCCTGGACCA GGCGCTGGAA GCGCTGGACA

Oryzae AAGAGAAGCT GATTCCGCAG GTCTGCTCCA GCCTGGATCA GGCCCTGGAC GCGCTGGACA

Campestris AAGAGAAGCT GATCCCGCAG GTCTGCTCCA GCCTGGACCA GGCCCTGGAG GCGCTGGACA

Xyl9a5C AGGAAAAATT GATCCCGCAG GTTTGTTCAA GTTTGGATCA GGCTCTTGAA GCATTGGACA

XylTemecul AGGAAAAATT GATCCCGCAG GTTTGTTCAA GTTTGGATCA GGCTCTTGAA GCATTGGACA

Burkholder AGGATGCGAA GATCCCGACC GTGTGCGCCG GCCTCGACCA GGCGCTCGAA GCGCTCGACA

Ralstonia AGGACGCAAA GATCCCGACC GTGTGCTCGA GCCTGGATCA GGCCCTCGAG TACCTGGACA

....|....| ....|....| ....|....| ....|....| ....|....| ....|....|

9665 9675 9685 9695 9705 9715

X.albiline CCGACCGCGA GTTCCTCAAG GCCGGCGGCG TGTTCACCGA CGACTTCATC GATGGCTACA

StenoK279a AGGACCGCGA GTTCCTGAAG GCCGGTGGCG TGATGAGCGA TGACTTCATC GACGGCTACA

StenoR551 AGGACCGTGA GTTCCTGAAG GCCGGCGGCG TGATGAGCGA TGACTTCATC GACGGCTACA

Vesicatori AGGACCGCGA GTTCCTCAAG GCCGGCGGCG TGATGAGCGA CGACTTCATC GACGGCTATA

Citri AGGACCGCGA GTTCCTCAAG GCCGGCGGCG TGATGAGCGA CGACTTCATC GACGGCTATA

Oryzae AGGACCGCGA GTTCCTCAAG GCCGGCGGCG TGATGAGCGA CGACTTCATC GACGGCTATA

Campestris AGGATCGCGA GTTCCTCAAG GCCGGCGGCG TGATGAGCGA CGACTTCATC GACGGCTACA

Xyl9a5C AAGACCGCGA TTTCTTGAAG GCAGGGGGTG TGATGAGTGA CGATTTTATT GACGGCTATA

XylTemecul AAGACCGCGA TTTCTTGAAG GCAGGCAGTG TGATGAGTGA CGATTTTATT AACGGCTATA

Burkholder AGGACCGCGA GTTCCTGACG CGCGGCGGCG TGTTCACGGA CGCGATGATC GACGCGTACC

Ralstonia AGGATCGCGA GTTCCTGACC CGCGGCGGCG TGTTCACCAA CTCGATGATC GACGCCTACA

....|....| ....|....| ....|....| ....|....| ....|....| ....|....|

9725 9735 9745 9755 9765 9775

X.albiline TCGCGCTGAA GATGCAGGAA GTGACCAAGT TCCGTGCGGC GACGCACCCG CTGGAATATC

StenoK279a TCGCGCTGAA GATGCAGGAA GTGACCAAGT TCCGCGCGGC CACCCACCCG CTGGAGTACC

StenoR551 TCGCGCTGAA GATGCAGGAA GTGACCAAGT TCCGCGCGGC CACCCACCCG CTGGAATATC

Vesicatori TCGCGCTGAA GATGCAGGAA GTCACCAAGT TCCGTGCTGC GACCCACCCG TTGGAGTACC

Citri TCGCGCTGAA GATGCAGGAA GTCACCAAGT TCCGTGCTGC GACCCACCCG TTGGAGTACC

Oryzae TCGCGCTGAA GATGCAGGAA GTCACCAAGT TCCGTGCTGC GACCCACCCG TTGGAGTACC

Campestris TCGCATTGAA GATGCAGGAA GTCACCAAGT TCCGCGCCGC AACGCATCCG CTGGAGTACC

Xyl9a5C TTGCGCTGAA GATGCAGGAA GTGACGCAGT TCCGCGCGGC CACACATCCG CTGGAATACC

XylTemecul TTGCGCTGAA GATGCAGGAA GTGACGCAGT TCCGCGCGGC CACACATCCG CTGGAATACC

Burkholder TGGGCCTGAA GGAGCAGGAG CTCGCGAAGT TCCGCATGAC GACGCACCCG ATCGAGTTCG

Ralstonia TCGAGCTGAA GATGGAAGAA GTCACCCGCT TCCGCATGAC CACGCACCCG GTCGAGTTCG

....|....| ....|....| ....|....| ....|....| ....|....| ....|....|

9785 9795 9805 9815 9825 9835

X.albiline AGCTGTATTA CGCCAACTGA ATGGACGAGA ACAAGAAG-- ---------- --CGC-----

StenoK279a AGCTGTACTA CGCCAGCTGA ATGGACGAGA ACAAGAAG-- ---------- --CGC-----

StenoR551 AGCTGTACTA CGCCAGCTGA ATGGACGAGA ACAAGAAG-- ---------- --CGC-----

Vesicatori AGCTGTACTA CGGCAACTGA ATGGACGAGA ACAAGAAG-- ---------- --CGC-----

Citri AGCTGTACTA CGGCAACTGA ATGGACGAGA ACAAGAAG-- ---------- --CGC-----

Oryzae AGCTGTACTA CGGCAACTGA ATGGACGAGA ACAAGAAG-- ---------- --CGC-----

Campestris AGCTGTACTA CGGCAACTGA ATGGATGAGA ACAAGAAG-- ---------- --CGC-----

Xyl9a5C AGATGTACTA CGCCAACTAA ATGGATGAGA ACAAGAAA-- ---------- --CGC-----

XylTemecul AGATGTACTA CGCCAACTAA ATGGATGAGA ACAAGAAA-- ---------- --CGC-----

Burkholder AGATGTACTA CTCGCTGTAA ATGGAAGAAA GCAAGAAAGG CTCCGGGCTG ACTGCCGAAA

Ralstonia AGATGTACTA CTCGCTGTAA ATGGAAGACG GCAAGAAGGC AGCCTCGATG AGCGCAGAAA

....|....| ....|....| ....|....| ....|....| ....|....| ....|....|

9845 9855 9865 9875 9885 9895

X.albiline --------GC GCTCGCGGCC GCCCTGAGTC AGATCGAAAA GCAGTTCGGC AAGGGCTCGG

StenoK279a --------GC CCTCGCTGCA GCTCTGGGCC AGATCGAAAA GCAGTTCGGC AAGGGCTCGG

StenoR551 --------GC CCTCGCTGCA GCTCTGGGCC AGATCGAAAA GCAGTTCGGC AAGGGCTCGG

Vesicatori --------GC CCTTTCCGCC GCACTGAGCC AGATCGAAAA GCAATTCGGC AAGGGCTCGG

Citri --------GC CCTTTCCGCC GCACTGAGCC AGATCGAAAA GCAATTCGGC AAGGGCTCGG

Oryzae --------GC CCTTGCCGCC GCACTGAGCC AGATCGAAAA GCAATTCGGC AAGGGCTCGG

Campestris --------GC CCTTTCCGCC GCCCTGAGCC AGATCGAAAA GCAATTCGGC AAAGGCTCGG

Xyl9a5C --------GC CCTTTCTGTC GCTTTAAGCC AGATTGAAAA ACAGTTCGGC AAGGGGTCCG

XylTemecul --------GC CCTTTCTGTC GCTTTAAGCC AGATTGAAAA ACAGTTCGGC AAGGGGTCCG

Burkholder AGAGCAAGGC GCTCGCCGCC GCGCTCGCGC AGATCGAGAA GCAGTTCGGC AAAGGGTCGA

Ralstonia AGCAGAAGGC GCTGGCTGCC GCGCTCGCGC AGATCGAAAA GCAGTTCGGC AAGGGCTCGA

....|....| ....|....| ....|....| ....|....| ....|....| ....|....|

9905 9915 9925 9935 9945 9955

X.albiline TGATGCGGAT GGGCGATCGT GTCATCG--- AAGCCGTGGA AGTGATTCCG ACCGGTTCGC

StenoK279a TGATGCGCAT GGGCGACCGC GTGGTCG--- AACCCGTCGA AGCCATCCCG ACCGGTTCGC

StenoR551 TGATGCGCAT GGGCGACCGT GTGGTCG--- AACCCGTCGA AGCCATCCCG ACCGGTTCGC

Vesicatori TCATGCGCAT GGGCGACCGC GTCATCG--- AGGCAGTCGA AGTCATCCCG ACCGGCTCGT

Citri TCATGCGCAT GGGCGACCGC GTCATTG--- AGGCGGTCGA AGTCATCCCG ACCGGCTCGC

Oryzae TCATGCGCAT GGGCGACCGT GTCATCG--- AAGCGGTCGA AGTCATTCCG ACCGGCTCGC

Campestris TGATGCGCAT GGGCGACCGG GTCATCG--- AGGCCGTCGA GGTCATCCCG ACCGGTTCGC

Xyl9a5C TGATGCGGAT GGGCGATCGC GTGATCG--- AGGCCGTAGA GGCGATCCCG ACAGGTTCGC

XylTemecul TGATGCGGAT GGGCGATCGC GTGATCG--- AAGCCGTAGA GGCGATCCCG ACAGGTTCGC

Burkholder TCATGCGGCT CGGCGACGGC GAGGCGGTCG AGGATATCCA GGTGGTGTCC ACGGGCTCGC

Ralstonia TCATGAAGAT GGGCGACGCC GAGGTGG--- AGCCGGTCCA GGTTGTGTCC ACCGGCTCGC

....|....| ....|....| ....|....| ....|....| ....|....| ....|....|

9965 9975 9985 9995 10005 10015

X.albiline TGATGCTGGA TATCGCGCTC GGCATCGGTG GCCTGCCGAA GGGCCGCGTG GTCGAGATTT

StenoK279a TGATGCTCGA CATCGCGCTG GGCATTGGTG GTCTGCCGAA GGGCCGTGTC GTTGAGATCT

StenoR551 TGATGCTCGA CATCGCGCTC GGCATTGGTG GTCTGCCGAA GGGCCGTGTC GTTGAGATCT

Vesicatori TGATGCTGGA TATCGCCCTG GGGATCGGCG GCCTGCCGAA GGGCCGCGTG GTCGAAATCT

Citri TGATGCTGGA TATCGCCCTG GGGATCGGCG GCCTGCCGAA GGGTCGCGTG GTCGAAATCT

Oryzae TGATGCTGGA TATCGCCCTG GGGACCGGCG GCCTGCCGAA GGGGCGCGTA GTCGAAATCT

Campestris TGATGCTGGA CATCGCACTG GGGATCGGCG GCCTGCCGAA GGGCCGCGTG GTCGAGATCT

Xyl9a5C TCATGTTGGA TCTGGCCCTG GGGATCGGTG GTTTGCCAAA GGGACGTGTC GTGGAAATCT

XylTemecul TCATGTTGGA TCTGGCCCTG GGGATAGGTG GTTTGCCAAA GGGACGTGTC GTGGAAATCT

Burkholder TCGGTCTCGA TATCGCGCTC GGCGTCGGTG GCTTGCCGCG CGGCCGTGTG GTCGAAATCT

Ralstonia TGGGTCTGGA CGTCGCGCTG GGCGTCGGCG GCCTGCCGCG CGGTCGCGTG GTCGAGATCT

....|....| ....|....| ....|....| ....|....| ....|....| ....|....|

10025 10035 10045 10055 10065 10075

X.albiline ACGGTCCGGA ATCCTCGGGC AAGACCACCC TGACCCTGCA GGCCATCGCT CAGTGTCAGA

StenoK279a ACGGGCCGGA ATCCTCGGGC AAGACCACCT TGACCCTGCA GGCCATCGCC GAATGCCAGA

StenoR551 ATGGTCCGGA GTCGTCGGGC AAGACCACCT TGACCCTGCA GGCCATCGCC GAGTGCCAGA

Vesicatori ACGGCCCGGA ATCCTCCGGC AAGACCACCT TGACCCTGCA AGCCATCGCC CAGTGCCAGA

Citri ACGGCCCGGA ATCCTCCGGC AAGACCACCT TGACCCTGCA AGCCATCGCC GAGTGCCAGA

Oryzae ACGGGCCGGA ATCCTCGGGC AAGACCACCC TGACCCTGCA GGCGATTGCC CAGTGTCAGA

Campestris ACGGGCCGGA ATCCTCGGGC AAGACCACCC TCACACTGCA GGCCATTGCC GAATGCCAGA

Xyl9a5C ATGGGCCGGA ATCTTCTGGG AAGACCACAT TGACTTTGCA GGCGATCGCT CAATGCCAGA

XylTemecul ATGGGCCGGA ATCTTCTGGG AAGACCACAT TGACTTTGCA GGCGATCGCT CAATGCCAGA

Burkholder ACGGGCCGGA ATCGTCCGGC AAGACGACGC TCACGCTGCA GGTGATCGCC GAGATGCAGA

Ralstonia ACGGCCCCGA ATCGTCCGGC AAGACCACGC TGACGCTGCA GGTGGTCGCC GAGATGCAGA

....|....| ....|....| ....|....| ....|....| ....|....| ....|....|

10085 10095 10105 10115 10125 10135

X.albiline AGAACGGCGG CACCGCTGCG TTCATTGATG CCGAACATGC ACTGGACCCG GTCTACGCTG

StenoK279a AGATGGGCGG CACCGCGGCG TTCATCGACG CCGAGCACGC GCTGGACCCG ATCTACGCCG

StenoR551 AGCTGGGCGG CACCGCAGCC TTCATCGACG CCGAGCATGC GCTGGATCCG ATCTACGCCG

Vesicatori AGAACGGCGG CACCGCTGCC TTCATCGACG CCGAGCACGC GCTGGACCCG ATTTATGCGG

Citri AGAACGGCGG CACCGCTGCC TTCATCGATG CCGAGCACGC ACTGGACCCG ATTTATGCGG

Oryzae AGCTGGGCGG CACCGCCGCC TTCATCGACG CCGAGCACGC GCTGGACCCG GTCTATGCGG

Campestris AGCTGGGCGG CACCGCGGCC TTCATCGACG CCGAGCATGC GCTGGACCCG ATCTATGCCG

Xyl9a5C AGAAGGGAGG TACAGCAGCG TTCATTGATG CTGAGCATGC CTTGGATCCG ATTTATGCGG

XylTemecul AGAGGGGAGG TACAGCAGCG TTCATTGATG CTGAGCATGC CTTGGATCCG ATTTATGCGG

Burkholder AGCTCGGCGG CACGGCGGCG TTCATCGACG CGGAGCACGC GCTCGACGTC CAGTATGCGT

Ralstonia AGCTGGGCGG CACCTGCGCC TTCATCGACG CCGAGCATGC GCTGGACGTC ACCTATGCCG

....|....| ....|....| ....|....| ....|....| ....|....| ....|....|

10145 10155 10165 10175 10185 10195

X.albiline CCAAGCTGGG CGTCAATGTC GACGAATTGC TGCTGTCGCA GCCGGATACC GGCGAACAAG

StenoK279a CCAAGCTGGG CGTGAACGTG GACGACCTGC TGCTGTCGCA GCCGGATACC GGTGAGCAGG

StenoR551 CCAAGCTGGG CGTCAACGTG GACGACCTGC TGCTGTCGCA GCCGGACACC GGCGAGCAGG

Vesicatori CCAAGCTGGG CGTCAACGTC GACGACCTGC TGCTGTCGCA GCCCGATACC GGTGAGCAGG

Citri CCAAGCTGGG CGTCAATGTC GACGACCTGC TGCTGTCGCA GCCGGATACC GGTGAGCAGG

Oryzae CCAAGCTGGG TGTCAACGTC GACGATCTGC TGCTCTCGCA GCCGGATACC GGCGAGCAGG

Campestris CCAAGCTGGG CGTCAATGTC GATGACCTGC TGCTGTCGCA GCCGGATACC GGTGAGCAGG

Xyl9a5C GCAAGTTAGG CGTCAATGTT GATGATTTGT TGTTGTCTCA GCCAGATACT GGGGAGCAGG

XylTemecul GCAAGTTAGG CGTCAATGTT GATGATTTGT TGTTGTCTCA GCCAGATACT GGGGAGCAGG

Burkholder CGAAGCTCGG CGTGAACGTG CCGGAGCTGC TGATCTCGCA GCCGGACACG GGTGAGCAGG

Ralstonia ACAAGCTCGG CGTGAAGGTG CCGGACCTGC TGATCTCCCA ACCGGACACC GGTGAGCAGG

....|....| ....|....| ....|....| ....|....| ....|....| ....|....|

10205 10215 10225 10235 10245 10255

X.albiline CGCTGGAAAT CGCCGATATG CTGGTGCGCT CGGGGTCGGT GGACATCGTG GTGATTGACT

StenoK279a CGCTGGAAAT CGCCGACATG CTGGTCCGTT CGGGTTCGGT CGACATCCTG GTGATCGACT

StenoR551 CGCTGGAAAT CGCCGACATG CTGGTCCGTT CGGGCTCGGT GGACATCCTG GTGGTCGACT

Vesicatori CACTGGAAAT TGCCGACATG CTGGTGCGCT CGGGTTCGGT TGATATCGTG GTGGTCGACT

Citri CACTGGAAAT TGCCGACATG CTGGTGCGTT CGGGTTCGGT TGATATCGTG GTGGTCGACT

Oryzae CGCTGGAAAT TGCCGACATG CTGGTGCGTT CAAGCTCGGT GGACATCGTG GTGATCGACT

Campestris CGCTGGAAAT CGCCGACATG CTGGTGCGTT CGAGCTCGGT GGACATCGTG GTGATCGACT

Xyl9a5C CTCTGGAAAT CGCTGACATG CTGGTGCGCT CGGGGTCGAT CGATATCATG GTCATTGATT

XylTemecul CTCTGGAAAT CGCTGACATG CTGGTGCGCT CGGGGTCGAT CGATATCATG GTTATTGATT

Burkholder CGCTCGAAAT CGTCGACGCG CTGGTCCGCT CGGGCTCGAT CGACATGATC GTCATCGACT

Ralstonia CGCTGGAAAT CGCTGACGCG CTGGTGCGCT CGGGCTCGGT CGACCTGATC GTCATCGACT

....|....| ....|....| ....|....| ....|....| ....|....| ....|....|

10265 10275 10285 10295 10305 10315

X.albiline CGGTCGCCGC GTTGACCCCA AAGGCCGAAA TCGAGGGTGA AATGGGCGAT CAACTGCCCG

StenoK279a CGGTTGCCGC GCTGACCCCG AAGGCCGAAA TCGAAGGCGA GATGGGCGAC CAGCTGCCAG

StenoR551 CGGTTGCCGC GCTGACCCCG AAGGCCGAAA TCGAAGGCGA GATGGGCGAC CAGCTGCCGG

Vesicatori CGGTTGCCGC ACTGACGCCA AAGGCGGAAA TCGAAGGCGA GATGGGCGAC CAGTTGCCTG

Citri CGGTCGCTGC ACTGACGCCG AAGGCGGAAA TCGAAGGCGA GATGGGCGAC CAGCTGCCAG

Oryzae CGGTTGCCGC ACTGACCCCG AAGGCAGAAA TCGAAGGCGA AATGGGCGAC CAGCTGCCGG

Campestris CGGTTGCCGC GCTGACCCCA AAGGCCGAAA TCGAAGGCGA GATGGGCGAT CAGCTGCCCG

Xyl9a5C CGGTTGCGGC ACTCACGCCA AGGGCGGAGA TTGAGGGTGA GATGGGAGAT CAGTTGCCCG

XylTemecul CGGTTGCGGC ACTCACGCCA AGGGCGGAGA TTGAGGGTGA GATGGGAGAT CAGTTGCCCG

Burkholder CGGTCGCGGC GCTCGTGCCG AAGGCCGAAA TCGAAGGCGA GATGGGCGAC GCGCTGCCCG

Ralstonia CGGTGGCCGC GCTGGTGCCC AAGGCCGAAA TCGAAGGCGA GATGGGCGAC GCGCTGCCCG

....|....| ....|....| ....|....| ....|....| ....|....| ....|....|

10325 10335 10345 10355 10365 10375

X.albiline GCCTGCAGGC GCGTCTGATG AGCCAGGCGC TGCGTAAGCT CACCGGCAAC ATCAAGCGCT

StenoK279a GCCTTCAGGC CCGCCTGATG AGCCAGGCGC TGCGCAAGCT GACCGGCAAC ATCAAGCGCT

StenoR551 GTCTGCAGGC CCGCCTGATG AGCCAGGCGC TGCGCAAGCT GACCGGCAAC ATCAAGCGCT

Vesicatori GTTTGCAGGC TCGCCTGATG AGCCAGGCGC TGCGCAAGCT CACCGGCAAT ATCAAGCGCT

Citri GTTTGCAGGC TCGCTTGATG AGCCAGGCGC TGCGCAAGCT CACCGGCAAT ATCAAGCGCT

Oryzae GCCTGCAGGC TCGCCTGATG AGCCAGGCGT TGCGCAAGCT TACCGGCAAC ATCAAGCGCT

Campestris GCCTGCAGGC CCGTTTGATG AGCCAGGCGC TGCGCAAGCT CACCGGCAAC ATCAAGCGCT

Xyl9a5C GTCTTCAGGC GCGCTTGATG AGCCAGGCAC TGCGTAAATT GACCGGCAAT ATCAAGCGCT

XylTemecul GTCTTCAGGC GCGATTGATG AGCCAGGCGC TGCGTAAATT GACCGGCAAT ATCAAGCGCT

Burkholder GCCTGCAGGC CCGCCTGATG TCGCAGGCGC TGCGCAAGCT GACGGGCACG ATCAAGCGCA

Ralstonia GTCTGCAGGC CCGCCTGATG AGCCAGGCGC TGCGCAAGCT GACCGGCACC ATCAAGCGCA

....|....| ....|....| ....|....| ....|....| ....|....| ....|....|

10385 10395 10405 10415 10425 10435

X.albiline CCAACACCCT GGTCGTCTTC ATCAACCAGT TGCGCATGAA GATCGGTGTG ATGATGCCGG

StenoK279a CCAACACCCT GGTGATCTTC ATCAACCAGC TGCGCATGAA GATCGGCGTG ATGATGCCGG

StenoR551 CCAACACCCT GGTGGTCTTC ATCAACCAGC TGCGCATGAA GATCGGCGTG ATGATGCCGG

Vesicatori CCAACACGCT GGTGGTCTTC ATCAATCAGC TGCGCATGAA GATCGGCGTC ATGATGCCGG

Citri CCAACACGCT GGTGGTCTTC ATCAATCAGC TGCGCATGAA GATCGGCGTC ATGATGCCGG

Oryzae CCAACACGCT GGTGGTCTTC ATCAATCAGC TGCGCATGAA GATCGGCGTC ATGATGCCGG

Campestris CCAACACGTT GGTGGTCTTC ATCAACCAGC TGCGCATGAA GATCGGCGTG ATGATGCCGG

Xyl9a5C CTAATACGCT GGTGATCTTT ATCAACCAGT TGCGTATGAA AATTGGGGTC ATGATGCCGG

XylTemecul CTAATACGCT GGTGATTTTT ATCAACCAGT TGCGTATGAA AATTGGGATC ATGATGCCGG

Burkholder CGAACTGCCT CGTGATCTTC ATCAACCAGA TCCGGATGAA GATCGGCGT- --GATGTTCG

Ralstonia CCAACTGCCT GGTGATCTTC ATCAACCAGA TCCGCATGAA GATCGGCGT- --GATGTTCG

....|....| ....|....| ....|....| ....|....| ....|....| ....|....|

10445 10455 10465 10475 10485 10495

X.albiline GGCAGAGCCC GGAAACCACC ACCGGCGGTA ACGCACTGAA GTTTTACGCA TCGGTGCGGT

StenoK279a GCCAGAGCCC GGAAACCACC ACGGGTGGCA ACGCGCTGAA GTTCTACGCC TCGGTCCGCC

StenoR551 GCCAGAGCCC GGAAGTGACC ACCGGCGGCA ACGCGTTGAA GTTCTATGCC TCGGTGCGCC

Vesicatori GCCAGAGCCC GGAAGTGACC ACCGGCGGCA ACGCGCTGAA GTTCTACGCC TCGGTGCGTC

Citri GCCAGAGCCC GGAAGTGACC ACCGGCGGCA ACGCGCTGAA GTTCTACGCC TCGGTGCGTC

Oryzae GCCAGAGCCC GGAAGTGACC ACCGGCGGCA ATGCCTTGAA GTTCTACGCC TCGGTGCGTC

Campestris GCCAGAGCCC GGAAGTGACC ACCGGCGGCA ATGCGCTGAA GTTCTACGCC TCGGTCCGCC

Xyl9a5C GTCAAAGCCC TGAAACTACG ACAGGGGGTA ATGCGCTGAA GTTCTATGCT TCAGTGCGTT

XylTemecul GTCAAAGCCC TGAAACTACG ACAGGGGGTA ATGCGCTGAA GTTCTATGCT TCTGTGCGTT

Burkholder GC---AACCC GGAAACCACG ACGGGCGGCA ACGCGCTGAA GTTCTACTCG TCAGTGCGTC

Ralstonia GC---TCGCC GGAAACCACC ACGGGCGGCA ATGCGCTCAA GTTTTACGCT TCGGTGCGTC

....|....| ....|....| ....|....| ....|....| ....|....| ....|....|

10505 10515 10525 10535 10545 10555

X.albiline TGGATATCCG CCGCATCGGC GCGATCAAGA AGGGCGATGA AATCATCGGC AACCAGACCA

StenoK279a TGGACATCCG CCGTATCGGC GCGATCAAGA AGGGTGACGA GATCATCGGT AACCAGACCA

StenoR551 TGGATATCCG CCGTATCGGC GCGATCAAGA AGGGCGACGA GATCATCGGC AACCAGACCA

Vesicatori TGGATATCCG CCGTATCGGT GCGATCAAGA AGGGCGACGA GATCATCGGC AACCAGACCA

Citri TGGATATCCG CCGTATCGGT GCGATCAAGA AGGGCGACGA GATCATCGGC AACCAGACCA

Oryzae TGGACATCCG TCGTATCGGC GCAATCAAGA AGGGCGACGA GATCATCGGC AACCAGACCA

Campestris TGGACATCCG CCGCATCGGT GCGATCAAGA AGGGTGACGA GATCATCGGC AACCAGACCA

Xyl9a5C TGGATATTCG CCGTATTGGC GCGATCAAGA AGGGTGACGA AATTATCGGG AATCAAACCA

XylTemecul TGGATATTCG CCGTATTGGC GCGATCAAGA AAGGTGACGA AATTATCGGG AATCAAACCA

Burkholder TCGACATTCG CCGGATCGGC TCGATCAAGA AGAACGACGA GGTGATCGGC AACGAAACGC

Ralstonia TGGACATCCG CCGCATCGGC TCGATCAAGA AGGGCGATGA GGTGGTCGGC AACGAGACCA

....|....| ....|....| ....|....| ....|....| ....|....| ....|....|

10565 10575 10585 10595 10605 10615

X.albiline AAATCAAGGT GGTCAAGAAC AAGTTGGCTC CCCCGTTCAA ACAGGTCGTC ACCGAGATCC

StenoK279a AGATCAAGGT CGTCAAGAAC AAGCTGGCAC CTCCGTTCAA GCAGGTCATC ACCGAGATCC

StenoR551 AGATCAAGGT CGTCAAGAAC AAGCTGGCGC CGCCGTTCAA GCAGGTCATC ACCGAGATCC

Vesicatori AGATCAAGGT GGTCAAGAAC AAGCTGGCGC CTCCGTTCAA GCAGGTCGTG ACCGAAATCC

Citri AGATCAAGGT GGTCAAGAAC AAGCTGGCGC CTCCGTTCAA GCAGGTCGTG ACCGAAATCC

Oryzae AGATCAAGGT GGTCAAGAAC AAGTTGGCGC CTCCGTTCAA GCAGGTCGTG ACCGAAATCC

Campestris AGATCAAGGT GGTCAAGAAC AAGCTGGCGC CTCCGTTCAA GCAGGTCATC ACCGAGATCC

Xyl9a5C AAATCAAGGT TGTTAAAAAC AAGTTGGCGC CTCCCTTCAA ACAAGTCGTG ACTGAGATTC

XylTemecul AAATCAAGGT TGTTAAAAAC AAGTTGGCGC CTCCCTTCAA ACAAGTCGTG ACTGAGATTC

Burkholder GCGTGAAGGT CGTCAAGAAC AAGGTGTCGC CGCCGTTCCG CGAAGCGATC TTCGACATCC

Ralstonia AGGTCAAGGT CGTCAAGAAC AAGGTGGCGC CGCCGTTCCG CGAGGCGATC TTCGACATCC

....|....| ....|....| ....|....| ....|....| ....|....| ....|....|

10625 10635 10645 10655 10665 10675

X.albiline TCTACGGCGA AGGCATCAGT CGCGAGGGCG AACTGATCGA CATGGGCGTG GAGGCCAAGC

StenoK279a TGTATGGCGA AGGCATCAGC CGTGAAGGCG AGCTGATCGA CATGGGTGTG GATGCCAAGC

StenoR551 TGTACGGCGA AGGCATCAGC CGCGAAGGCG AACTGATCGA CATGGGCGTG GATGCCAAGC

Vesicatori TGTATGGCGA AGGCATCAGC CGCGAGGGCG AATTGATCGA CATGGGCGTG GAAGCCAAGC

Citri TGTATGGCGA AGGCATCAGT CGCGAGGGCG AATTGATCGA CATGGGCGTG GAAGCCAAGC

Oryzae TGTACGGCGA AGGCATCAGC CGCGAAGGCG AATTGATCGA TATGGGCGTG GAAGCCAAGC

Campestris TGTACGGCGA AGGCATCAGC CGCGAGGGTG AGCTGATCGA TATGGGCGTG GAAGCCAAGC

Xyl9a5C TCTATGGTGA AGGCATCAGT CGTGAAGGGG AATTGATTGA GATGGGCGTG GAGGCCAAGT

XylTemecul TCTATGGTGA AGGCATCAGT CGTGAAGGGG AATTGATTGA AATGGGCGTG GAGGCCAAGT

Burkholder TGTACGGCGA AGGCATTTCG CGCCAGGGCG AGATCATCGA TCTCGGCGTG CAGGCGAAGA

Ralstonia TCTACGGTGC GGGCGTGTCG CGCGAAGGCG AGATCATCGA TCTGGGCGTG GAAGCCAAGG

....|....| ....|....| ....|....| ....|....| ....|....| ....|....|

10685 10695 10705 10715 10725 10735

X.albiline TGGTTGAAAA GGCCGGTGCC TGGTATAGCT ACGGCAGCGA GCGCATCGGC CAGGGCAAGG

StenoK279a TGGTCGAGAA GGCTGGCGCC TGGTACAGCT ACGGTGAAGA ACGCATTGGC CAGGGCAAGG

StenoR551 TGGTCGAGAA GGCCGGCGCC TGGTACAGCT ACGGCGAAGA GCGTATCGGC CAGGGCAAGG

Vesicatori TGGTCGACAA GGCCGGCGCC TGGTACAGCT ACGGCGATGA GCGCATCGGG CAGGGCAAGG

Citri TGGTCGACAA GGCCGGCGCC TGGTACAGCT ATGGCGATGA GCGCATCGGG CAGGGCAAGG

Oryzae TGGTTGAAAA GGCCGGCGCC TGGTACAGCT ACGGCGATGA GCGCATCGGG CAGGGCAAGG

Campestris TGGTCGACAA GGCAGGTGCC TGGTACAGCT ACGGCGACGA GCGCATCGGC CAGGGCAAGG

Xyl9a5C TGGTTGAGAA AGCCGGCGCT TGGTATAGCT ACGGTGGTGA GCGGATCGGG CAAGGAAAAG

XylTemecul TGGTTGAGAA AGCCGGCGCT TGGTATAGCT ATGGTGGTGA GCGGATCGGG CAAGGAAAAG

Burkholder TCGTCGACAA GGCGGGCGCG TGGTACAGCT ATAGCGGCGA GAAGATCGGC CAGGGCAAGG

Ralstonia TGGTCGAGAA ATCCGGCGCC TGGTACAGCT ACAACGGCGA GCGCATCGGT CAGGGCCGTG

....|....| ....|....| ....|....| ....|....| ....|....| ....|....|

10745 10755 10765 10775 10785 10795

X.albiline ACAACGCGCG CGGCTACCTG CGCGACAACC CTCAGGTTGC CGCCAAGTTG GAAAGCGAAC

StenoK279a ACAACGCCCG CGGCTACCTG CGCGACAACC CGACTGTCGC CGCCAAGCTC GAGGCCGAGC

StenoR551 ACAATGCCCG TGGCTACCTG CGCGACAACC CGACCGTCGC CGCCAAGCTC GAAGCCGAGC

Vesicatori ACAACGCGCG GACCTACCTG CGCGACAACC CGCAGGTTGC GACCCGGCTG GAAGCCGAGT

Citri ACAACGCGCG AACCTACCTG CGCGACAACT CGCAGGTTGC GACCCGGCTG GAAGCGGAGC

Oryzae ACAATGCGCG GACTTACCTG CGCGACAACC CGCAGGTTGC GGTCCGGCTG GAAGCCGAAC

Campestris ACAACGCCCG TGGGTATCTG CGCGACAACC CGCAGGTGGC GATCAAGCTC GAAGCCGAGC

Xyl9a5C ATAATGCGCG CGGCTATCTA CGTGAAAACC CGCATCTTGC GGCCAAGCTT GAGGCTGATT

XylTemecul ATAATGCGCG TGGCTATCTA CGCGAAAACC CGCATTTTGC GGCCAAGCTT GAGGCTGATT

Burkholder ACAACGCGCG CGAATTCCTG CGCGAAAATC CGGAAATCGC GCGCGAGATC GAGAATCGCA

Ralstonia ACAACTGCCG CGAATTCCTG CGCGAAAACG CCGAACTGGC CCGCGAGATC GAAAACAAGG

....|....| ....|....| ....|....| ....|....| ....|....| ....|....|

10805 10815 10825 10835 10845 10855

X.albiline TGCGCGAGAA GTTCCAGCCC ACGGAAATCG CGCCCAGCCC AGCC---GAT GCCGAAGACC

StenoK279a TGCGCGAGAA ATTCCAGCCG GCCGAAGCGG CCCGCGAGGA AGGC---GAC GACGAAGGCG

StenoR551 TGCGCGAGAA GTTCCAGCCG TCTGAAGCTG CCCGCGAAGA AGGC---GAT GACGAAGGCG

Vesicatori TGCGTGAGAA GTTCCAGCCT GCCGAAGCAC CGCGCGAGGC CGGC---GAC GACGAAGACA

Citri TGCGTGAGAA GTTCCAACCT GCCGAAGCAC CGCGTGAAGC CGGC---GAC GACGAAGACA

Oryzae TGCGCGAGAA GTTCCAGCCT GCCGAAGCGC CGCGCGAAGC CGGC---GAC GACGAAGAGA

Campestris TGCGCGAGAA GTTCCAGCCC GCCGAGGCGC CGCGTGAAGC CGGC---GAG ACCGAGAGCG

Xyl9a5C TGCGTGAGAA ATTTGAGCCG ACCGAGCTTT CTCGAGAAGA GGGC---GAT GAAGATACGC

XylTemecul TGCGTGAGAA ATTTGAGCCG ACCGAGCTTT CTCGAGAAGA GGGC---GAT GAAGATACGC

Burkholder TTCGCGAGTC GCTCGGAGTC GCCGCGATGC CGCAGGGCGC CGGTTCCGAG GCCGAGATCA

Ralstonia TCCGCGAACA CCTGGG---- --CGTGACGC CGATGGGCGC CGTCACGCTG GCCGAGGAAG

....|....| ....|..

10865 10875

X.albiline TCGAGGCTTA A------

StenoK279a AAGACGAATA A------

StenoR551 ACGACGAATA A------

Vesicatori AGGAATAA-- -------

Citri AGGAGTAA-- -------

Oryzae AGGAATAA-- -------

Campestris AGTGA----- -------

Xyl9a5C TCGAAGATAC AATGTAA

XylTemecul TCGAAGATGC AATGTAA

Burkholder TGGACGAAGA GGAGTAA

Ralstonia TCGAGGAAGA TTGA---
